# “Topological Significance” Analysis of Gene Expression and Proteomic Profiles from Prostate Cancer Cells Reveals Key Mechanisms of Androgen Response

**DOI:** 10.1371/journal.pone.0010936

**Published:** 2010-06-03

**Authors:** Adaikkalam Vellaichamy, Zoltán Dezső, Lellean JeBailey, Arul M. Chinnaiyan, Arun Sreekumar, Alexey I. Nesvizhskii, Gilbert S. Omenn, Andrej Bugrim

**Affiliations:** 1 Departments of Pathology, Internal Medicine, Human Genetics, School of Medicine, University of Michigan, Ann Arbor, Michigan, United States of America; 2 GeneGo, Inc., St. Joseph, Michigan, United States of America; Duke-NUS Graduate Medical School, Singapore

## Abstract

**Background:**

The problem of prostate cancer progression to androgen independence has been extensively studied. Several studies systematically analyzed gene expression profiles in the context of biological networks and pathways, uncovering novel aspects of prostate cancer. Despite significant research efforts, the mechanisms underlying tumor progression are poorly understood. We applied a novel approach to reconstruct system-wide molecular events following stimulation of LNCaP prostate cancer cells with synthetic androgen and to identify potential mechanisms of androgen-independent progression of prostate cancer.

**Methodology/Principal Findings:**

We have performed concurrent measurements of gene expression and protein levels following the treatment using microarrays and iTRAQ proteomics. Sets of up-regulated genes and proteins were analyzed using our novel concept of “topological significance”. This method combines high-throughput molecular data with the global network of protein interactions to identify nodes which occupy significant network positions with respect to differentially expressed genes or proteins. Our analysis identified the network of growth factor regulation of cell cycle as the main response module for androgen treatment in LNCap cells. We show that the majority of signaling nodes in this network occupy significant positions with respect to the observed gene expression and proteomic profiles elicited by androgen stimulus. Our results further indicate that growth factor signaling probably represents a “second phase” response, not directly dependent on the initial androgen stimulus.

**Conclusions/Significance:**

We conclude that in prostate cancer cells the proliferative signals are likely to be transmitted from multiple growth factor receptors by a multitude of signaling pathways converging on several key regulators of cell proliferation such as c-Myc, Cyclin D and CREB1. Moreover, these pathways are not isolated but constitute an interconnected network module containing many alternative routes from inputs to outputs. If the whole network is involved, a precisely formulated combination therapy may be required to fight the tumor growth effectively.

## Introduction

Prostate cancer is one of the most commonly diagnosed cancers and the second leading cause of cancer-related death in North American men [1]. While androgen withdrawal therapy is often effective initially, most cases progress to the much more aggressive androgen-independent phenotype. Despite significant research efforts, the mechanisms underlying tumor progression are poorly understood. Roles for several signaling pathways have been established, but not a systemic picture. For example, IGF signaling has been implicated in the progression from androgen-dependent to androgen-independent states [2], but also has been shown to suppress AR trans-activation via FoxO1 and thus have inhibitory effects on the growth of prostate cancer cells [3], EGF was reported to mimic effects of androgen on the gene expression and independently stimulate growth of androgen-dependent prostate cancer cells [4]. Other studies have produced evidence of interplay between androgen signaling and TGF-beta [5],[6], FGF [7],[8] and VEGF [9].

Most of the research cited above has been hypothesis-driven rather than data-driven. Hypothesis formulation is susceptible to bias due to investigators' preferences and current research trends about what is perceived as “interesting”. A complementary data-driven approach using high-throughput molecular profiling and advanced data analysis algorithms could enhance understanding of the many cellular processes that underlie progression of prostate cancer to the androgen-independent stage and could pave the way to new therapies and to achieve greater efficacy from better directed use of existing therapies.

Genome-wide expression profiling haven been widely applied to complex diseases, including prostate cancer [4],[10],[11],[12],[13],[14]. Several recent studies also systematically analyzed gene expression profiles in the context of biological networks and pathways, uncovering novel aspects of prostate cancer [15],[16],[17]. Despite this progress, truly systemic analysis which would take into account both gene expression and proteomic data from the same sample remains an elusive goal. A critical challenge is to perform robust integrated analysis of the datasets produced by so different molecular platforms. This is a hard informatics problem because microarray and proteomics data could not, in most cases, be directly compared to each other. For example, studies in yeast have shown that correlation between levels of mRNA and corresponding proteins were insufficient to make reliable predictions about protein levels from gene expression data [18]. A recent study of prostate cancer specimens showed concordance between proteomic and genomic data ranging from 46% to 68% based on the “absent/present” calls; however, correlations were low when actual levels of expression were compared [19]. As shown in a recent work [20], much more extensive quantitative protein characterization leads to significant improvement in correlation between levels of protein and gene expression. Still, there are multiple intrinsic sources of the discordance, including mRNA degradation, alternative splicing, translational regulation, post-translational modifications, and protein degradation [21]. These cannot be overcome by technology improvements alone and have to be addressed by new analytical approaches to data integration. Earlier efforts in this area utilized pre-defined sets of genes (pathways, Gene Ontology categories) to look for concordance between proteomic and genomic data on this level [22],[23].

Recently we have developed a new computational methodology which may help to advance integrated analysis of multiple types of data one step further [24]. Our approach combines disease- or condition-specific, high-throughput molecular data with the global network of protein interactions to identify nodes which occupy significant network positions with respect to differentially expressed genes or proteins in the presented molecular datasets. Even when there is significant noise and discordance in the data itself, predictions of the algorithm are likely to converge on a common set of signaling proteins in the pathways responsible for changes in the expression of target genes and proteins. Often the activity of such signaling proteins is modified by subtle post-translational modifications, binding to second messengers, or recruitment to a particular sub-cellular locale. These events are not explicitly reflected in corresponding molecular profiles; thus, they remain “hidden” from the standard molecular assays. Our methodology is able to find many such “hidden” proteins by identifying sets of their likely downstream targets and assessing enrichment of such sets by differentially expressed genes or proteins. We call this procedure “topological scoring” (refer to “Methods” section for more details).

In our earlier work this method was tested on a set of microarray gene expression data from psoriatic patients where it was able to correctly identify many key regulatory proteins whose relation to the disease is confirmed by independent studies [24]. In the present study we have applied the topological scoring approach to investigate the response of LNCap prostate cancer cells to treatment with synthetic androgen (R1881), as a well-studied model system for prostate cancer progression. We took a data-driven approach, without having any preconceived hypothesis regarding cellular processes activated by androgen in these cells. We have collected and analyzed both gene expression and proteomic data in order to cross-validate predictions based on different types of data and evaluate the utility of this approach to integrative data analysis.

## Results

### Genes and proteins affected by androgen treatment identified by microarray and protein mass-spectrometry

In order to interrogate the role of androgen in prostate cancer, the androgen-responsive prostate cancer cell line LNCaP was treated with synthetic androgen R1881 (see “Methods” section for details). LNCaP cells treated with androgen showed increased cell proliferation whereas the control cells stopped growing in the androgen depleted medium. Using statistical analysis of gene expression data we have identified 347 and 257 genes that were up- and down-regulated, respectively, in treated vs. untreated cells (FDR≤1%) (Table S1). The up-regulated genes included known androgen-induced genes such as Kallikrein 3 (*KLK3*; a.k.a. *PSA*), FK506 binding protein 5 (FKBP5), N-myc downstream regulated 1 (NDRG1) and fatty acid synthase (FASN). Using iTRAQ 2DLC-MS/MS-based proteomic profiling of androgen-treated vs. untreated LNCap cells, we have identified 70 and 39 proteins that were elevated or down-regulated, respectively, in treated cells compared to untreated cells (Table S1) (Details of the mass spectrometry and statistical analyses are described in [25]). The androgen-regulated protein data set included gene products for the known up-regulated genes mentioned above, as well as several other proteins previously known and unknown to be regulated by androgen. Sets of up-regulated genes and proteins have 13 common members which is ∼17% of the smaller set. For down-regulated genes and proteins the level of concordance is ∼8%.

### Topologically significant nodes in the global signaling network

In order to investigate putative signaling mechanisms which activate gene and protein expression after androgen stimulation, we have applied our recently developed technique of topological significance analysis [24]. We submitted the lists of up-regulated genes and proteins to the on-line version of our topological scoring tool (http://topology.genego.com/zcgi/topology_scoring.cgi) to identify key regulatory proteins whose activity in treated cells might have accounted for changes in gene and protein levels. Gene expression and proteomic data were submitted to the scoring procedure separately, resulting in two sets of topologically significant regulatory proteins. Each node in the global network of protein interactions was assigned topological scores (topological p-values) with respect to each set of molecular data. To control the false discovery rate (FDR) the significance level filter was applied. Using FDR≤5% we identified 962 topologically significant proteins from gene expression data and 577 topologically significant proteins from proteomic data (Table S2). Interestingly, the two sets of topologically significant proteins contain 301 common elements (or 52% of the smaller set).This result is in stark contrast with only 17% overlap between lists of up-regulated genes and up-regulated proteins.

### Growth factor signaling network is highly implicated in androgen response

**Figure 1:**
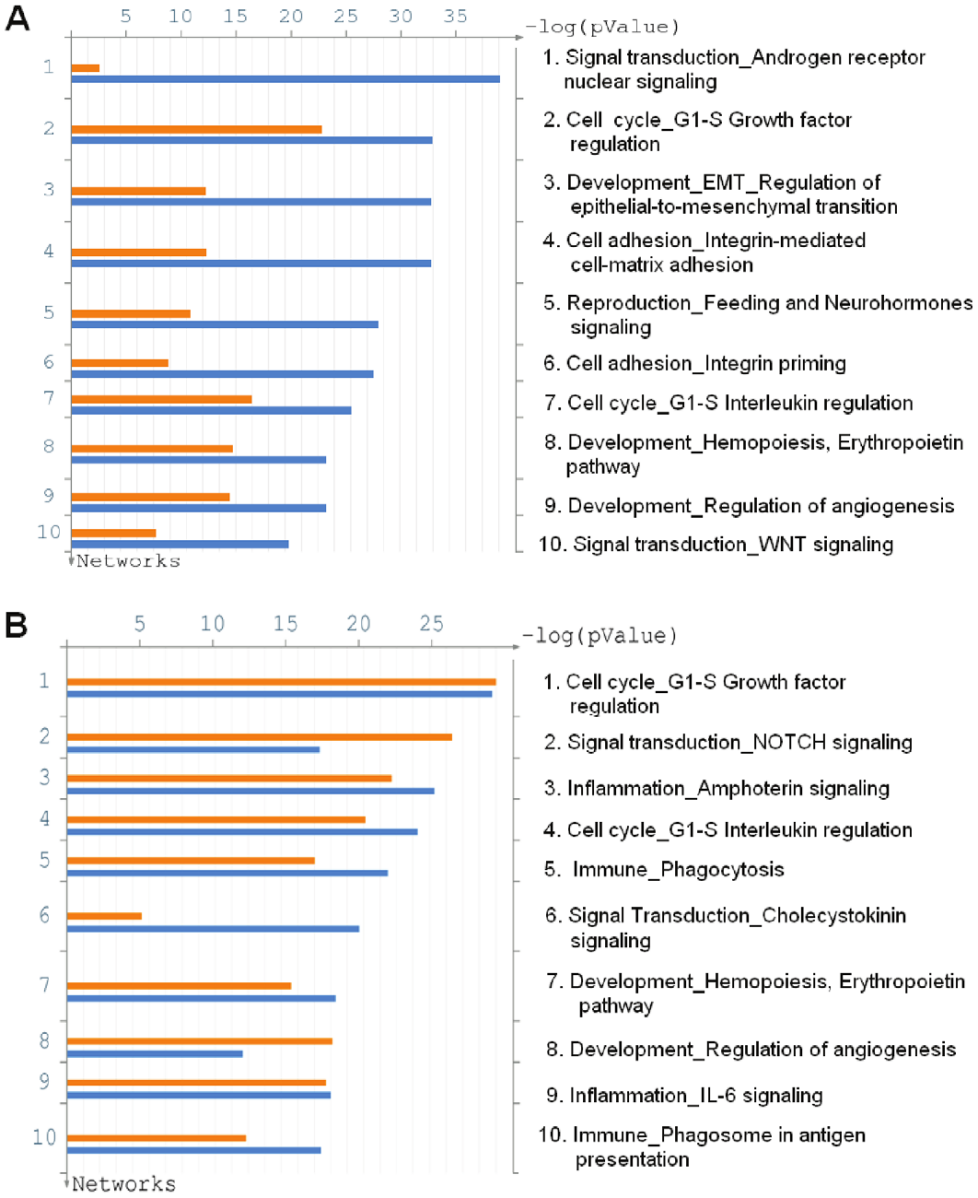
Functional analysis of topologically significant proteins. (A) Enrichment of GeneGo process networks by topologically significant proteins identified using all up-regulated genes and proteins. (B) Enrichment of GeneGo process networks by topologically significant proteins identified using truncated sets of data (excluding genes and proteins directly regulated by androgen receptor). Orange bars–enrichment by significant proteins identified using proteomics data set. Blue bars–enrichment by significant proteins identified using gene expression data.

**Figure 2:**
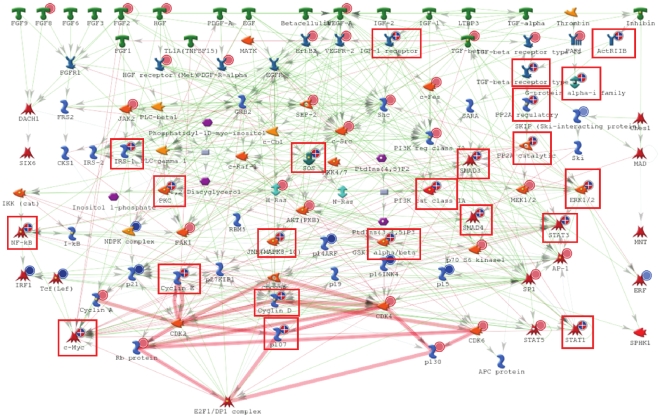
Growth factor regulation of the G1-S transition in cell cycle. Red dots indicate proteins identified as topologically significant using the gene expression profile. Blue dots indicate proteins identified as topologically significant using the proteomics profile. Red boxes–proteins identified as topologically significant from both sets of data.

For functional analysis, both sets of the topologically significant proteins were loaded into the MetaCore™ software package (GeneGo, Inc.) where we calculated enrichment in the ontology of functional processes as defined by “GeneGo process networks”. We used all proteins in the MetaCore network (“default” setting) as the reference list for calculating enrichment p-values. As would be expected, the top-scoring process is “Androgen receptor nuclear signaling” (Figure 1a). Surprisingly, however, this process is highly enriched only in proteins whose topological scores are derived from the gene expression profile; 82 of 126 nodes in this process network are deemed significant with respect to over-expressed genes. In contrast, only 19 nodes are deemed significant with respect to up-regulated proteins from the iTRAQ dataset. The next highly enriched process network is “Growth factor regulation of cell cycle”. Unlike androgen signaling, this network is highly enriched in proteins that are topologically significant for both gene expression and proteomic data. Of 186 nodes in this network, 95 are highly scored with respect to over-expressed genes while 63 are highly scored with respect to iTRAQ-identified proteins up-regulated after androgen treatment. In combination, 49 nodes are confirmed as topologically significant from both sets of molecular data. Close examination of this process reveals that topologically significant proteins are present on all levels of signaling hierarchy, including several Growth factors (EGF, FGF, VEGF-A), receptors (IGFR, EGFR, ActRIIB, VEGFR-2), signaling kinases (AKT, GSK3, PI3K, JNK, ERK1/2, PKC), transcription factors (c-Myc, IRF1, Tcf(Lef), SMAD3, SMAD4, STAT1, STAT3) and, finally, cyclin kinases (Cyclin D, Cyclin E) that directly regulate cell cycle (Figure 2). Importantly, topological significance of many of these proteins was confirmed for both datasets. For comparison we have also performed pathway enrichment analysis of the original sets of up-regulated genes and proteins. Interestingly, the majority of identified pathway maps for both gene expression and proteomics sets are related to metabolic processes, most of them to fatty acid metabolism (Table S3). Additionally, several signaling pathways are revealed by this analysis, notably Growth factor signaling via MAPK and PIK3, regulation of lipid metabolism and one pathway map related to cell cycle. However, none of the signaling pathways is very highly ranked and overall significance of the enrichment is low compared to the results obtained for proteins identified by topological scoring. Enrichment of GeneGo networks by up-regulated proteins does reveal Androgen signaling network, but it is also at the bottom of the list (p = 0.007). Except for the insulin signaling there seem to be no consistency between networks enriched in up-regulated genes and up-regulated proteins. Overall it appears that functional analysis of differentially expressed genes and proteins tends to identify core target pathways, such as metabolism, while the anaylysis of topologically significant proteins reveals key signaling processed activated in androgen-stimulated cells.

In order to investigate whether significant differences in sizes of the sets used in our analysis could have affected the results we randomly sampled the pool of genes and proteins and added them to the differentially expressed sets. This step was followed by enrichment analysis of the extended sets. However, the results show that no new maps or networks become significant for larger sets and moreover, significance of previously identified maps and networks steadily declines as more random genes are added. (See Table S4).

### Delineating androgen-dependent and androgen-independent activity

The results presented above suggest that a majority of proteins in the signaling network connecting multiple growth factors to regulation of cell cycle may become active after androgen stimulation. The resulting activation of cell proliferation could become a key contributing mechanism for the switch to androgen independence in prostate cancer. To further verify this conjecture we need to investigate whether or not this result depends on the direct activity of androgen receptor. Thus, our next step was to delineate signaling effects that are independent of direct activation of androgen receptor.

First, we used MetaCore™ to identify which of the over-expressed genes and up-regulated proteins are direct targets of transcriptional regulation by androgen receptor. To this end we built the “nearest neighbors” network around androgen receptor with the interaction filter in MetaCore set to allow only “transcriptional regulation” type of links. Lists of up-regulated genes and proteins were mapped onto the resulting network. Using this network we further selected nodes that are both: “downstream” of androgen receptor and have experimental data associated with them. We found 45 direct targets of androgen receptor among over-expressed genes and 9 targets among the set of up-regulated proteins. These molecules were excluded from the original lists and truncated sets were re-analyzed with the topological significance tool with subsequent functional analysis of topologically scored nodes in MetaCore™. We identified 565 significant proteins on the basis of iTRAQ data and 668 significant proteins on the basis of gene expression dataset (with FDR<5%, Table S5). One observation immediately noticeable from the examination of the enrichment diagram is the absence of the androgen signaling network (Figure 1b). This absence confirms that many proteins in the androgen pathway received high topological scores on the strength of over-expression of a large number of direct targets of androgen receptor. Once these targets are eliminated from consideration, scores for proteins in the androgen-regulated pathway dropped below significance level. In contrast, high enrichment for the network of growth factor regulation of cell cycle remained virtually intact. While number of nodes on this network scored on the basis of microarray data decreased from 95 to 78, the number of nodes scored based on iTRAQ data increased from 63 to 71. The overlap between the two sets of significant proteins also increased to 54 (or 76% of the smaller set). This finding supports the suggestion that activity of this pathway is independent of direct androgen action and may represent important mechanisms for the switch to androgen-independent proliferation in prostate cancer.

### Top-ranked regulatory proteins and their pathways

Next we examined the top-ranked molecules in the sets of topologically scored proteins. Our goal was to determine specific transcription factors which drive gene expression response after androgen treatment and identify regulatory cascades that activate them. There are several transcription factors that can regulate expression of significant numbers of “targets” among over-expressed genes or up-regulated proteins or both (Table 1). For example c-Myc has 25 targets among 70 up-regulated proteins identified by iTRAQ and 63 targets among 347 over-expressed genes identified by microarray analysis. c-Myc is ranked #1 in topological scoring based on iTRAQ data and #11 in the scoring based on gene expression (still in the top 2%). Other transcription factors that received high topological scores with respect to both datasets are SREBP1 and YY1, which are important regulators of enzymes involved in lipid and fatty acids metabolism. In contrast, CREB1 and ATF-4 are the top-scoring transcriptional regulators with respect to microarray data but they do not receive any score based on the iTRAQ data. The reason for such discrepancy is lack of significant number of CREB1 and ATF-4 targets among up-regulated proteins identified by mass-spectrometry (Table 1). This may indicate activity of some posttranscriptional processes blocking synthesis or inducing degradation of these proteins at the time of sampling. While transcription factors often receive high topological scoring due to the significant number of their direct targets in the experimental datasets, the upstream signaling molecules are scored based on the enrichment of sets of their “remote targets”–genes and proteins a few steps downstream on signaling pathways.

**Table 1:** Scoring of transcriptional regulators with significant number of direct targets among up-regulated genes and proteins.

Transcription factor	iTRAQ data set		Affymetrix dataset	
	# of direct targets in dataset	Scoring percentile	# of direct targets in dataset	Scoring percentile
**c-Myc**	**25**	**99.9**	**63**	**98.6**
ATF4	0	Un-scored	10	99.6
CREB1	2	Un-scored	37	99.8
P53	5	Un-scored	37	Un-scored
**SREBP1**	**8**	**78.0**	**14**	**99.3**
SP1	15	Un-scored	64	Un-scored
Ikaros	4	93.0	2	Un-scored
**YY1**	**6**	**95.8**	**7**	**90.0**

**Figure 3:**
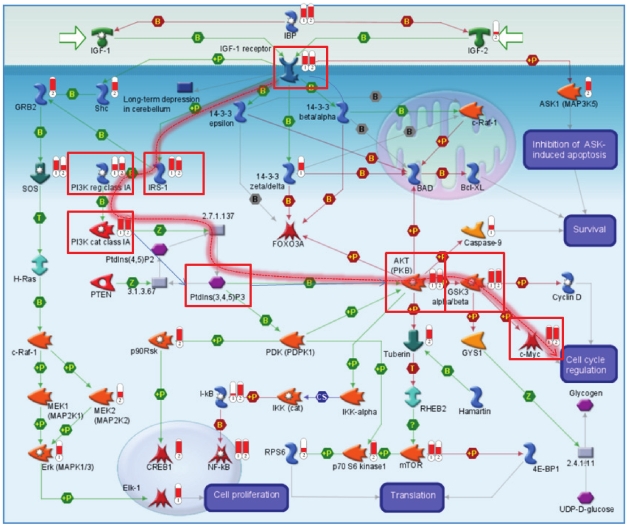
Map for IGF signaling showing topologically significant genes identified from using “truncated” sets of Affymetrix and iTRAQ data. Red level in the “thermometers” represents relative rank (percentile) of a protein in the corresponding list of topologically significant proteins. The number identifies the dataset from which significance was calculated: 1-iTRAQ, 2-Affymetrix. Red boxes and highlighted path illustrate signaling cascade with strongest support from both sets.

Examination of individual signaling cascades leading to the top transcriptional regulators reveals that PI3K signaling is supported by consistently high topological scores derived from both proteomics and microarray datasets. Figure 3 shows this cascade in the context of IGF signaling. The PI3K cascade is highlighted by the red line, while all of its elements that achieve high topological scores with respect to both sets are marked by red boxes. Such consistent scoring suggests the central role of this pathway in regulating events that follow androgen treatment. Most likely, its role in this system is inhibition of GSK3 kinase and its ability to phosphorylate c-Myc and cyclin D (Fig. 3). Normally such phosphorylation would target these molecules for proteolysis, thus limiting cell proliferation. In this situation, however, c-Myc appears to be persistently activated judging by the high number of its direct targets present in both sets. One likely reason for the persistent activity of PI3K signaling is homozygous mutation of PTEN in LNCaP cells leading to the lack of its expression in this system [26]. This effect may be exacerbated by the combination of high level of Kallikrein 3, over-expression of IGF receptor, and under-expression of IGF-binding proteins (IBPs). Kallikrein 3 (also known as PSA) is highly up-regulated in prostate cancer and is consistently over-expressed on both mRNA and protein levels in our experimental data. It was previously shown that PSA has proteolytic potential with respect to IGF-binding proteins [27],[28]. Moreover, it was suggested that this might be a mechanism by which bioavailability of IGF is increased, contributing to the growth of prostate cancer cells [29],[30].

In our analysis we have obtained several additional pieces of evidence to support this hypothesis. First, IGF-binding proteins received high topological scores based on both microarray and iTRAQ data. This result confirms that they are highly relevant to observed changes in gene and protein expression following androgen treatment of LNCap cells. Second, upon androgen treatment we found that expression levels of at least one of the IGF-binding proteins (IBP3) and of IGF receptor shift in opposite directions. IBP3 is 30% under-expressed in treated cells, while IGF-receptor is 46% over-expressed. Down-regulation of IBP3 on the genomic level in addition to proteolytic activity of PSA would contribute to lower concentration of IBP3 protein and increased availability of IGF. The resulting higher level of IGF is matched by over-expression of its receptor, leading to high activity of downstream pathways.

## Discussion

### Growth factor network as the main response module to androgen stimulation in LNCap cells

Our topological analysis identified the network of growth factor regulation of cell cycle as the main response module for androgen treatment in LNCap cells. As described in the introduction, different aspects of growth factor signaling have been extensively investigated in the context of the prostate cancer switch to androgen-independent mode. Our results support these earlier observations from a complementary systems-level, data-driven perspective. Instead of focusing on activity of individual proteins, we show that the majority of signaling nodes in the network connecting multiple growth factors to key regulators of cell cycle occupy significant positions with respect to the observed gene expression and proteomic profiles elicited by androgen stimulus. This network contains multiple conventional “pathways” transmitting signals from growth factor receptors. These include signaling via MAP kinases, PI3K pathway and signaling via SMADs and cross-talk among these systems. Thus it is reasonable to conclude that in prostate cancer cells the proliferative signals are transmitted from growth factor receptors by a multitude of signaling pathways converging on several key regulators of cell proliferation such as c-Myc, Cyclin D and CREB1. Moreover, these pathways are not isolated but constitute an interconnected network module containing many alternative routes from inputs to outputs.

Our results further indicate that growth factor signaling probably represents a “second phase” cell response to androgen stimulus. When all direct targets of androgen receptor are removed from consideration, most proteins in the growth factor network are still highly scored with respect to the remaining sets of over-expressed genes and proteins. This response might be mediated by combined effects of high levels of PSA and growth factor receptors and low levels of growth factor inhibitors, such as IGF-binding proteins (IBPs) (Fig. 3). Proteolytic action of PSA may further contribute to the lowering of IBPs levels. At the same time, PSA expression can be sustained independently of androgen receptor by CREB1 and some other transcription factors [31]. When these factors are activated via growth factor signaling pathways, a positive feedback loop arises that can sustain high levels of PSA and cell proliferation even in the absence of activated androgen receptor. We have noted that CREB1 is ranked #1 in topological scoring of gene expression data, implying that it is highly active in this system.

Although further experimental work, such as siRNA studies is needed to confirm these inferences, if proved correct they may lead us to reconsider our approach to finding targeted therapies for prostate cancer. Biological networks are robust in a sense that there are many alternative ways to transmit a molecular signal from one point to another. Given high mutation rates of genes in cancer cells, it is likely that, even if we block a certain cascade with a targeted drug, there will be at least a sub-population of cells in a tumor which could circumvent such a block by using an alternative signaling route. If the whole network is involved, a precisely formulated combination therapy will be required to fight the tumor growth effectively. Moreover, such combination therapies might have to be specific for a small subpopulation of patients or even individual patients given patient-specific properties of oncogenic networks.

### Dynamic nature of cellular responses and integration of data generated by different technologies

In this study, concurrent measurements of gene expression and protein levels following the treatment with synthetic androgen were performed, and hundreds of genes and dozens of proteins whose levels increased following the stimulus were indentified. However, there is only modest overlap (about 17%) observed between the sets of up-regulated genes and proteins. While initially this sounds surprising, this result should be expected. Cells are complex dynamic systems in which processes occur on multiple time scales. When we assay a biological sample we are taking a static snapshot of this dynamic behavior. For example levels of mRNA may increase after 20–60 min following the treatment but the protein synthesis could be further delayed, and statistically significant change in protein concentrations will take much longer to develop and have smaller ratios. By the time proteins are synthesized some mRNA could be degraded, leaving no trace of gene over-expression. Thus, when studying microarray or proteomic data, we are dealing with fragmented traces of activity that are left behind by transient dynamic processes on different levels of cellular machinery. Even in the experiments where samples are assayed at several different time-points we are still looking at a small collection of individual snapshots rather than the full picture of cellular dynamics.

Here we used the concept of topological significance to reconstruct upstream pathways that might have resulted in these traces of dynamic activity which we detected as observable molecular profiles. The results indicate that this approach was successful in predicting key regulatory proteins and pathways such as androgen signaling, growth factor signaling and regulation of cell cycle that mediate responses of LNCap cells to treatment with synthetic androgen (R1881). Most importantly, we have discovered that the degree of overlap between sets of regulatory proteins predicted from gene expression and proteomic data is much higher than the overlap between the experimental sets themselves (52% vs. 17%). Moreover, for the growth factor regulation of the cell cycle which appears to be a key process in this system, the overlap reaches 76%. This provides a good indication that predictions converge on the same set of regulatory proteins despite significant paucity in the experimental data. Taken together, these observations show that our approach could be instrumental in translating high-throughput datasets generated by vastly different technologies into consistent predictions of activity of underlying signaling pathways and key regulatory proteins.

## Methods

### Cell culture and androgen treatment

Detailed procedures of cell culture and androgen treatment are described in the recent PLoS publication [25]. Briefly, LNCaP (ATCC number: CRL-1740™) cells were grown in RPMI 1640 medium (Invitrogen, Carlsbad, CA) supplemented with 10% fetal bovine serum under 5% CO2 and 90% humidity. At 70% confluence, the cells were subjected to androgen deprivation in phenol red-free RPMI 1640 medium supplemented with charcoal-stripped fetal bovine serum. After two days, androgen (R1881) was added at 1 nm final concentration for 48 hrs. The control cells were treated with corresponding amount of ethanol used to dissolve androgen.

### RNA Isolation and Microarray Analysis

Total RNA isolation was performed using TRIZOL reagent as per the manufacturer's instructions (Invitrogen) [25]. Control and androgen treated cells were washed with PBS (phosphate buffered saline) and scraped in TRIZOL reagent. 250 µl of chloroform was added to 1 ml of sample and mixed by inversion. The sample was centrifuged at 13,000 rpm for 15 min at 4°C. 500 µl of isopropyl alcohol was added to supernatant and centrifuged at 13,000 rpm for 15 min at 4°C. The resulting pellet was washed with 70% ethanol, and centrifuged at 13,000 rpm for 10 minutes at 4°C. The RNA pellet was dried and purified using RNeasy mini kit (QIAGEN Inc., Valencia, CA). The amount and integrity of purified RNA was checked by NanoDrop ND-1000 Spectrophotometer (NanoDrop Technologies, Wilmington, DE) and Agilent Bioanalyzer (Agilent Technologies). RNAs isolated from three biological replicates were used for microarray analysis performed on Affymetrix U133 Plus 2.0 arrays (Affymetrix, Inc., Santa Clara, CA). Complementary DNA synthesis, cRNA synthesis, hybridization, washing, and scanning were done following the manufacturer's protocols (Affymetrix, Inc.). Expression values were calculated after normalization of the data based on previously published procedure [32]. Statistical Q test was done to calculate false discovery rate (FDR), a FDR≤1% was applied to identify androgen-regulated genes. The data is publicly available at GEO public repository website (accession number is GSE17044). All data is MIAME compliant as detailed on the MGED Society website.

### Proteomic analysis

Details of the iTRAQ- mass spectrometry and data analysis are described in detail in [25]. Briefly, a double duplex iTRAQ experiment was performed using the four-plex iTRAQ reagent (iTRAQ® Reagents Multiplex Kit, Applied Biosystems, Foster City, CA). Proteins from duplicate sets of androgen-treated and control cells were isolated using urea-thiourea containing buffer and equal amounts of proteins were used for labeling by iTRAQ reagents as per the manufacturer instructions (Applied Biosystems). After labeling, all four samples were combined, and subjected to two-dimensional fractionation by strong cation exchange (SCX) and reverse phase liquid chromatography (RP-LC). The RP-LC fractions were directly plated onto MALDI plates online by infusing with the MALDI matrix alpha-cyano-4-hydroxycinnamic acid. Tandem mass spectrometric data were acquired using 4800 Proteomics Analyzer -TOF/TOFTM (Applied Biosystems) linked to 4000 Series Explorer software (v. 3.0). MS/MS spectra were extracted from the raw data in Mascot Generic File format and converted to mzXML using IP Framework (www.proteomecommons.org) for SEQUEST search against Human IPI database version 3.24 appended with an equal number of decoy sequences. In total, 8580 SEQUEST search results (all from singly-charged spectra) were obtained and further processed using PeptideProphet and ProteinProphet, leading to the identification of 3686 peptides mapping to 904 proteins. Of these, 3550 peptides were quantified with post-data normalization, which mapped to 875 proteins. The estimated FDR using target-decoy strategy was below 0.5%. After a median centered normalization of peak areas of peptides, relative protein expression (treated vs. control) ratios were determined for each protein. A threshold ratio for defining differentially expressed proteins was set based on control replicate sample; proteins with iTRAQ ratios of > = 1.2 and < = 0.83 were considered up-regulated and down-regulated, respectively.

### Algorithm for topological scoring of regulatory proteins

**Figure 4:**
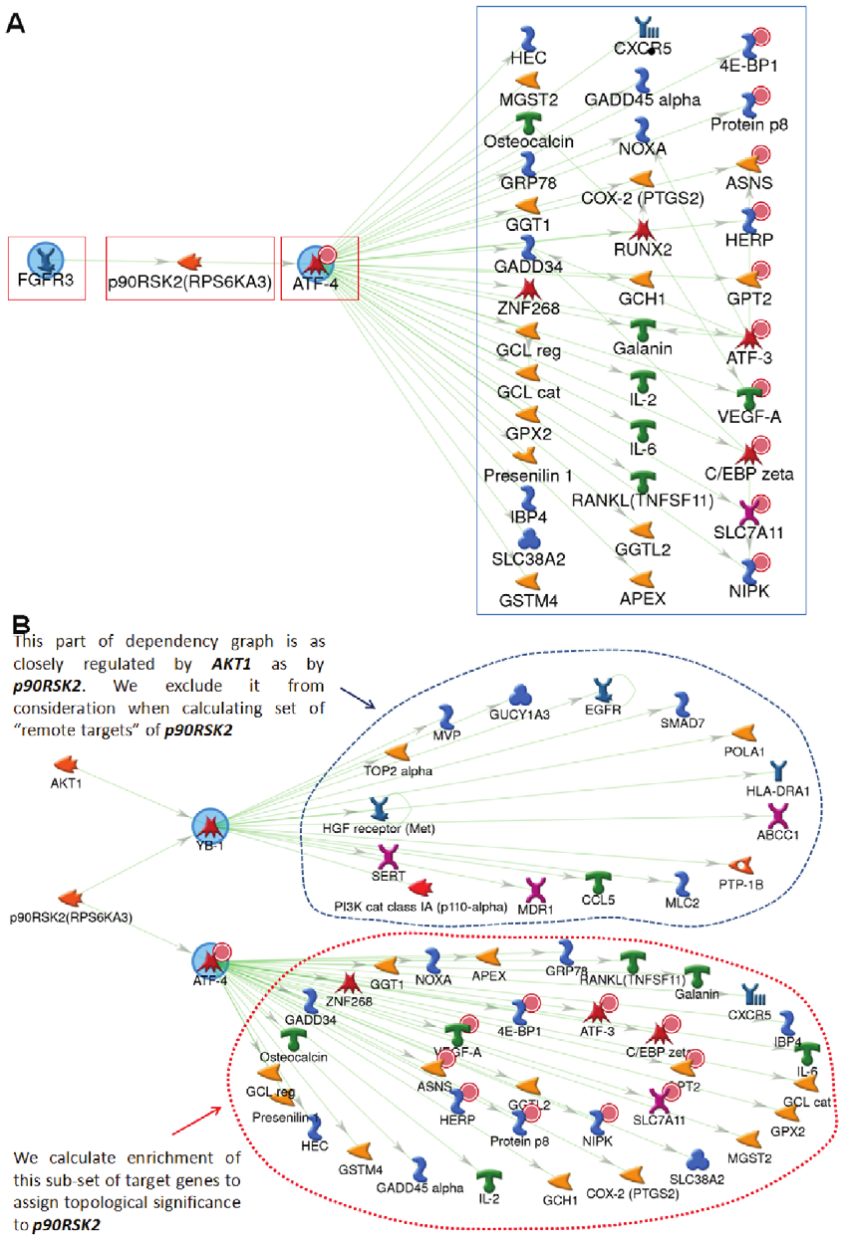
“Hidden” regulatory proteins in signaling pathways. (A) FGFR3 and p90RSK2 in this simple network are not affected on the gene expression level, therefore remaining “hidden” from a microarray assay. We identify sets of remote targets associated with each of the signaling molecules and assess their enrichment with differentially expressed genes. Remote targets of FGFR3 and p90RSK2 are proteins within the blue box. They could be many steps downstream of signaling proteins. *Topological significance* is assigned to regulatory nodes based on the enrichment of associated sets of target genes. Red boxes indicate topologically significant nodes in this network. (B) Topological scoring of regulatory nodes in complex networks with competitive regulation. (See text for details).

The identification of key regulatory proteins was performed using freely available online tool provided by GeneGo Inc. (http://topology.genego.com/zcgi/topology_scoring.cgi). The algorithm starts with a set of differentially expressed genes or proteins derived from biological samples. Typically these genes or proteins are identified by the standard statistical analysis of data produced by one of the available experimental high-throughput techniques. We map such sets of genes or proteins onto a global database of protein-protein interactions. The online tool used in this study utilizes GeneGo's MetaBase™ knowledgebase containing approximately 300,000 protein-protein and protein-small molecule interactions manually extracted from the literature by expert annotators. The simplified concept of topological scoring is illustrated in Fig. 4a. This figure shows a signaling cascade leading from FGFR3 receptor to a handful of target genes downstream of transcription factor ATF-4. Gene expression data from the present study are mapped onto this network. Up-regulated genes are indicated by red circles associated with gene symbols. While ATF-4 itself is over-expressed, neither the receptor nor the kinase p90RSK2 which mediates signaling are up-regulated on genomic level. To identify whether or not signaling proteins occupy topologically significant positions with respect to up-regulated genes, we consider network dependency graphs for each of them. Tracing these graphs downstream to target genes allows us to associate a subset of downstream targets with each of the signaling molecules. On Fig. 4a the target genes in the blue box on the right can be associated with FGFR3 and p90RSK2 as their “remote targets”, while for ATF-4 they are direct targets. The final step is to calculate enrichment of target gene-sets (remote or direct) associated with each regulatory node with experimental data and determine the statistical significance of such enrichment. A set of targets enriched in differentially expressed genes would imply activity of corresponding regulator(s). In our example, 10 out of 36 target genes are over-expressed, rendering FGFR3, p90RSK2 and ATF-4 topologically significant. To summarize, the general concept is to use topological properties of the global protein interaction network to identify non-local regulation patterns and associate a set of putative targets with each signaling molecule of interest, then assess expression profiles of these targets and infer activity of corresponding regulators. Given the role of network topology in this assessment, we have named this procedure “topological significance scoring”.

One caveat regarding the example above is that in real protein interaction networks the picture is more complicated than simple tree-like structures like the one shown on Fig. 4a. Most likely one has to deal with a highly interconnected web of interactions where many competitive cascades can regulate the same target. Additionally, dependency graphs for many important signaling proteins are huge, containing thousands of potential targets. For example, the full dependency graph of p90RSK2 contains 49 potential “remote targets” many of which can also be regulated by competitive pathways. Our algorithm overcomes these issues by considering overlaps of dependency graphs originating from different regulatory nodes, thus taking into account competitive regulation of target genes. The idea is illustrated in Fig. 4b which shows an extended fragment of dependency graph of p90RSK2. In addition to target genes regulated via ATF-4 this network contains signaling via transcription factor YB-1. None of the additional target genes is up-regulated in our dataset. This significantly reduces the overall enrichment of the whole set of remote targets of p90RSK2. The solution is to find a subset of targets within the dependency graph of p90RSK2 that are fairly “specific” to this kinase, so that the pattern of their expression bears more relevance to its activation status. To this end we consider alternative regulators to which some of the downstream nodes could be attributed. In this example we identify node in this graph are at least as close or closer to the competitive regulator AKT1 as they are to p90RSK2. The proximity can be calculated either using simple network distance metrics (number of steps) or taking into account additional information such as trust levels of interactions and knowledge on well established “canonical” pathways. The nodes that are at least as close to AKT1 are excluded from consideration and enrichment is calculated for the remaining part of the dependency graph (At this point the problem is reduced to the analysis of gene set enrichment and any of the existing methods can be applied [33],[34],[35]. In our technique this procedure is repeated for multiple competitive regulators. We select the best score to characterize protein of interest (p90RSK2 in this example).

## Supporting Information

Table S1 The list of up- and down regulated genes and proteins. We have performed concurrent measurements of gene expression and protein levels following the treatment of LNCaP prostate cancer cells with synthetic androgen. Using statistical analysis of gene expression data we have identified 347 and 257 genes that were up- and down-regulated, respectively, in treated vs. untreated cells (FDR<1%). Using iTRAQ 2DLC-MS/MS-based proteomic profiling of androgen-treated vs. untreated LNCap cells, we have identified 70 and 39 proteins that were elevated or down-regulated, respectively, in treated cells compared to untreated cells.
(0.23 MB PDF)

Table S2 List of topologically significant genes and proteins determined from gene expression and proteomics data. The list of up-regulated genes and proteins were submitted to the scoring procedure separately, resulting in two sets of topologically significant regulatory proteins. We identified 962 topologically significant proteins from gene expression data and 577 topologically significant proteins from proteomic data (FDR<5%).
(0.52 MB PDF)

Table S3 Enrichment analysis of up-regulated genes and proteins. The list of pathway maps and GeneGo process networks significantly enriched in up-regulated genes and proteins. An FDR threshold of 0.05 was used for maps and 0.2 for networks.
(0.07 MB PDF)

Table S4 Enrichment analysis of randomly extended up-regulated genes and proteins. In order to investigate whether significant differences in sizes of the sets used in our analysis could have affected the results we randomly sampled the pool of genes and proteins and added them to the differentially expressed sets.
(0.07 MB PDF)

Table S5 List of topologically significant genes and proteins determined from truncated sets of up-regulated genes and proteins. We removed the direct targets of androgen receptor from the list of up-regulated genes and proteins. Direct targets were determined as those proteins to which androgen receptor has a direct “transcription regulation” type of interaction. The resulting truncated sets were re-analyzed with the topological significance tool and 565 significant proteins and 668 significant genes identified (FDR<5%).
(0.43 MB PDF)
