# Supplementary material for: “Topological Significance” Analysis of Gene Expression and Proteomic Profiles from Prostate Cancer Cells Reveals Key Mechanisms of Androgen Response
Source: PLoS One. 2010 Jun 3;5(6):e10936. doi: 10.1371/journal.pone.0010936 (PMC2880599; doi:10.1371/journal.pone.0010936)
Supplement: Table S1 — The list of up- and down regulated genes and proteins. We have performed concurrent measurements of gene expression and protein levels following the treatment of LNCaP prostate cancer cells with synthetic androgen. Using statistical analysis of gene expression data we have identified 347 and 257 genes that were up- and down-regulated, respectively, in treated vs. untreated cells (FDR<1%). Using iTRAQ 2DLC-MS/MS-based proteomic profiling of androgen-treated vs. untreated LNCap cells, we have identified 70 and 39 proteins that were elevated or down-regulated, respectively, in treated cells compared to untreated cells. (0.23 MB PDF) [file pone.0010936.s001.pdf]

Up-regulated genes in treated vs. untreated cells

Symbol	Entrez Id	Description
SCN3	10456	HCLS1 associated protein X-1
ZBTB10	65986	zinc finger and BTB domain containing 10
PTHRP, HHM, I	5744	parathyroid hormone-like protein
FLVCRL14q	55640	feline leukemia virus subgroup C cellular receptor family, member 2
hDIP	1831	TSC-22 related protein
LDLR	3949	low density lipoprotein receptor
PPFIBP2	8495	PTPRF interacting protein, binding protein 2 (liprin beta 2)
dJ142L7.2, FLJ	51175	epsilon-tubulin
WFDC4	6590	secretory leukocyte protease inhibitor (antileukoproteinase)
MGC132003	2746	glutamate dehydrogenase 1
VIMP	55829	selenoprotein S
SAS	54187	sialic acid synthase
MGC4504	79094	ChaC, cation transport regulator-like 1 (E. coli)
OAT	4942	ornithine aminotransferase (gyrate atrophy)
DAPL1	92196	death associated protein-like 1
LTF	4057	neutrophil lactoferrin
RP4-678I3	23566	calcium-mobilizing lysophosphatidic acid receptor LP-A3
FLJ22660	55839	centromere protein N
SLC25A37	51312	mitochondrial solute carrier protein
NCU-G1	112770	kidney predominant protein NCU-G1
UCC1	54749	ependymin related protein 1 (zebrafish)
LOC221710	221710	LOC221710
KIAA1834	63916	engulfment and cell motility 2
SBBI03	10193	neuregulin receptor degradation protein-1
MGC150532	116285	medium-chain acyl-CoA synthetase
RALDH3	220	retinaldehyde dehydrogenase 3
hPIP1	55003	PAK/PLC-interacting protein 1
TROP2, GA733	4070	tumor-associated calcium signal transducer 2
bA108L7.1	81621	Kazal-type serine peptidase inhibitor domain 1
KLHL29	114818	kelch-like 29 (Drosophila)
PRO1855, FLJ2	55379	leucine rich repeat containing 59
STQTL11	1021	cell division protein kinase 6
ACCA	31	acetyl-Coenzyme A carboxylase alpha
PSCA	8000	prostate stem cell antigen
MGC102869	284001	coiled-coil domain containing 57
LOC81691	81691	exonuclease NEF-sp
ZNF145	7704	zinc finger protein 145
dJ417M14.2	4117	serine/threonine protein kinase MAK
dJ955L16.1	222546	regulatory factor X domain containing 1
hR-PTPu	5797	protein tyrosine phosphatase, receptor type, mu polypeptide
KDELR3	11015	KDEL (Lys-Asp-Glu-Leu)
FLJ97621	9590	kinase scaffold protein gravin
TAHCCP2	25953	paroxysmal nonkinesiogenic dyskinesia
SLC7A1	6541	solute carrier family 7 (cationic amino acid transporter, y+ system)
P8	26471	p8 protein

p31	5714	26S proteasome regulatory subunit p31
LRIG1	26018	leucine-rich repeats and immunoglobulin-like domains 1
LOC653698	653698	LOC653698
RHR	153	beta-1-adrenergic receptor
TRAPG	6747	SSR gamma
RNS4	6038	ribonuclease, RNase A family, 4
Ssc2	54898	elongation of very long chain fatty acids
C3orf25	90288	chromosome 3 open reading frame 25
DUSP27	92235	dual specificity phosphatase 27 (putative)
MANK	56172	progressive ankylosis protein
seladin-1	1718	putative protein product of Nbla03646
beta4Gal-T1	2683	UDP-Gal:betaGlcNAc beta 1,4-galactosyltransferase, polypeptide 1
KIAA1338	440275	eukaryotic translation initiation factor 2 alpha kinase 4
UNQ847	493869	EPLA847
hCTR1, CTR1	1317	solute carrier family 31 (copper transporters), member 1
xCT	23657	cystine/glutamate transporter
SLC16A6	9120	solute carrier family 16, member 6
MIWC	361	mercurial-insensitive water channel
ST4, CRYBG1	202	suppression of tumorigenicity 4 (malignant melanoma)
SF20	56005	stromal cell-derived growth factor
bA375E1.3	25803	SAM pointed domain containing ets transcription factor
TMEM45A	55076	transmembrane protein 45A
MCCC2	64087	methylcrotonoyl-Coenzyme A carboxylase 2 (beta)
Ang2	285	Tie2-ligand
SpS	6611	spermidine aminopropyltransferase
TU12	3992	fatty acid desaturase 1
PED	8682	homolog of mouse MAT-1 oncogene
TIS7	3475	nerve growth factor-inducible protein PC4
ELL2	22936	ELL-related RNA polymerase II, elongation factor
ERO1L	30001	ERO1 (S. cerevisiae)-like
VLDLRCH	7436	very low density lipoprotein receptor
ps-PLA1	51365	phosphatidylserine-specific phospholipase A1alpha
SPCS4C	90701	SEC11-like 3
BETA2AR	154	catecholamine receptor
FLJ25390	221481	chromosome 6 open reading frame 81
RAM	5873	RAB27A, member RAS oncogene family
TMCO3	55002	transmembrane and coiled-coil domains 3
NMDMC	10797	methylene tetrahydrofolate dehydrogenase 2
TMEPAI	56937	transmembrane prostate androgen-induced protein
PIG13	81563	chromosome 1 open reading frame 21
IG/EBP-1	1054	CCAAT/enhancer binding protein gamma
SQLE	6713	squalene epoxidase
STK39	27347	Ste20-like protein kinase
TMEM27	57393	transmembrane protein 27
KRT18	3875	cytokeratin 18
SAH	6296	SA hypertension-associated homolog
GTC90	10466	component of oligomeric golgi complex 5

GALE	2582	galactowaldenase
SAP1	2005	SRF accessory protein 1
NO3	4681	neuroblastoma, suppression of tumorigenicity 1
TDD5	10397	differentiation-related gene 1 protein
STATI2, SSI2, S	8835	suppressor of cytokine signaling-2
MGC13170	84798	multidrug resistance-related protein
CLDN8	9073	claudin 8
Rcal, PIG20, FL	5954	proliferation-inducing gene 20
MGC74725	54676	GTP binding protein 2
TS11	440	asparagine synthetase
HSD10	161835	fibrous sheath interacting protein 1
S100P	6286	migration-inducing gene 9
LOC144481	144481	LOC144481
c-MAF	4094	Avian musculoaponeurotic fibrosarcoma (MAF) protooncogene
MGC43380	4211	Meis1, myeloid ecotropic viral integration site 1 homolog
MGC105112	4015	protein-lysine 6-oxidase
TM4SF1	4071	tumor-associated antigen L6
SMAD1	2617	Charcot-Marie-Tooth neuropathy, neuronal type, D
PFKFB2	5208	PFKFB, cardiac
PART1	25859	PART1
SSAT-1	6303	spermidine/spermine N1-acetyltransferase
PCNXL2	80003	pecanex-like 2
ATF3	467	activating transcription factor 3
ISG20	3669	interferon stimulated exonuclease gene 20kDa
IRS2	8660	insulin receptor substrate 2
ZAG	563	alpha-2-glycoprotein 1, zinc
WWTR1	25937	transcriptional co-activator with PDZ-binding motif (TAZ)
MGC132014	8821	inositol polyphosphate-4-phosphatase, type II, 105kDa
PGDH1	3248	15-hydroxyprostaglandin dehydrogenase
KLF5	688	transcription factor BTEB2
NNMT	4837	nicotinamide N-methyltransferase
UGT2B4	7363	UDP-glucuronyltransferase, family 2, beta-4
NKX3A	4824	NK3 homeobox 1
hFZ8	8325	frizzled 8
SLC41A1	254428	solute carrier family 41 member 1
HERC5	51191	cyclin-E binding protein 1
RNF188	79872	Casitas B-lineage lymphoma-like
WDR37	22884	WD repeat domain 37
B2M	567	beta chain of MHC class I molecules
bHLHe40	8553	differentially expressed in chondrocytes 1
Dip2, KIAA018	23181	DIP2 disco-interacting protein 2 homolog A (Drosophila)
PPAPDC1B	84513	diacylglycerol pyrophosphate like 1
TMCC3	57458	transmembrane and coiled-coil domains 3
p64H1	25932	chloride intracellular channel 4
RAB39B	116442	RAB39B, member RAS oncogene family
KIAA1001	22901	Arylsulfatase G
bHLHa15	168620	muscle, intestine and stomach expression 1

slow-type	4604	skeletal muscle C-protein
RALT	54206	mitogen-inducible gene 6 protein
hTC-1	56892	thyroid cancer-1
TBRG1	84897	nuclear interactor of ARF and MDM2
MGC111507	353322	low density lipoprotein receptor-related protein binding protein
PSAT1	29968	endometrial progesterone-induced protein
mSLO1	3778	bA205K10.1 (potassium large conductance calcium-activaed channel)
SOUL	23593	placental protein 23
MGC104991	3570	interleukin 6 receptor alpha subunit
Ska1	220134	spindle and KT (kinetochore) associated 1
KDELR2	11014	ERD-2-like protein
TCF5	1051	transcription factor 5
BTG1	694	B-cell translocation protein 1
SLC26A3	1811	solute carrier family 26, member 3
dJ1103G7.3, TI	57761	p65-interacting inhibitor of NF-kappaB
hcp-6	23310	non-SMC condensin II complex, subunit D3
TLH	5352	lysyl hydroxylase 2
RP11-402G3.2	203197	chromosome 9 open reading frame 91
MLD, Des-1, DI	8560	dihydroceramide desaturase
MGC70414	1622	diazepam binding inhibitor, splice form 1c
MGC4154	1649	growth arrest- and DNA damage-inducible
SLC43A1	8501	solute carrier family 43, member 1
FLJ11773	60673	hypothetical protein LOC60673
NIBAN	116496	niban protein
TMP, CL-20	2012	epithelial membrane protein 1
MGC54182	51303	FK506 binding protein 11, 19 kDa
SRPRB	58477	signal recognition particle receptor, beta subunit
SLC4A5	57835	sodium bicarbonate transporter 4
SPAL2	57568	SPA-1-like 2
PPM1E	22843	partner of PIX 1
nicein-150kDa,	3909	epiligrin alpha 3 subunit
hSK2	3781	potassium intermediate
KIAA0293, CDF	23316	cut-like 2
KIAA1254	9236	cell cycle progression 8 protein
STK37	23178	PAS-serine/threonine kinase
LOC401623	401623	LOC401623
MGC15366	3880	keratin, type I, 40-kd
YTS, YRS, tyrRS	8565	tyrosine tRNA ligase 1, cytoplasmic
YMR292W	51026	golgi transport 1 homolog B
TNFAIP9	79689	STEAP family member 4
P4501B1	1545	cytochrome P450, family 1, subfamily B, polypeptide 1
SLC3A2	6520	antigen defined by monoclonal antibody 4F2, heavy chain
TXREB	468	activating transcription factor 4
TC	1604	decay accelerating factor for complement
NICAL	64780	microtubule associated monoxygenase
MRP4, EST170	10257	ATP-binding cassette, sub-family C, member 4
NBPF15	284565	hypothetical protein LOC284565

dJ292B18.2	25902	methylenetetrahydrofolate dehydrogenase
U19	55840	ELL-associated factor 2
VPF	7422	vascular permeability factor
PICD	3417	oxalosuccinate decarboxylase
ODC1	4953	ornithine decarboxylase 1
STK17B	9262	serine/threonine kinase 17b
TAP1	23541	tocopherol-associated protein
hG28K	58480	ras homolog gene family, member U
NYD-sp9	9848	microfibrillar-associated protein 3-like
MGC1405	3638	INSIG-1 membrane protein
WIPI49	55062	WIPI-1 alpha
TMEM79	84283	transmembrane protein 79
MGC163279	2081	endoplasmic reticulum to nucleus signaling 1
MGC15563	84962	ajuba
rap1GAPII	5909	RAP1, GTPase activating protein 1
U-MAF	23764	transcription factor MAFF
GPT2	84706	glutamic pyruvate transaminase (alanine aminotransferase) 2
DC13	56942	hypothetical protein LOC56942
SERS	6301	seryl-tRNA synthetase
SEC24D	9871	SEC24 (S. cerevisiae) related gene family, member D
PLML	5349	FXYD domain-containing ion transport regulator 3
TMPRSS2	7113	epitheliasin
FLJ23563	79993	elongation of very long chain fatty acids-like 7
YVTM2421	64778	factor for adipocyte differentiation 104
SCD1	6319	acyl-CoA desaturase
NRXN4	26047	homolog of Drosophila neurexin IV
TXNL1CL	57095	chromosome 1 open reading frame 128
RNF69	5192	peroxisome biogenesis factor 10
RTP801, FLJ20!	54541	RTP801
TNS3	64759	tumor endothelial marker 6
MGC18216	3480	insulin-like growth factor 1 receptor
DLX1	1745	distal-less homeobox 1
STYI	55808	GalNAc alpha-2, 6-sialyltransferase I
MGC72127	22998	LIM and calponin homology domains 1
bHLHc5	55897	mesoderm posterior 1 homolog (mouse)
FLJ22590	27034	acyl-coenzyme A dehydrogenase 8
WASH3P	374666	WAS protein family homolog 3 pseudogene
MCT	79648	microcephaly, primary autosomal recessive 1
claudin-1	1366	claudin 7
NF-IL6-beta	1052	CCAAT/enhancer binding protein delta
DNASE2B	58511	endonuclease DLAD
PHPS1-2	11057	abhydrolase domain containing 2
SLC2A3	6515	GLUCOSE TRANSPORTER TYPE 3, BRAIN
MGC87297	3915	formerly LAMB2
hVPLA(2)	5322	phosphatidylcholine 2-acylhydrolase
Vesl-2	9455	cupidin
PRO2194	2181	fatty-acid-Coenzyme A ligase, long-chain 3

EDEM3	80267	ER degradation-enhancing -mannosidase-like protein 3
MLT1	10892	MALT associated translocation
MGC43127	9935	transcription factor MAFB
WWC1	23286	WW, C2 and coiled-coil domain containing 1
SDK1	221935	sidekick 1
NSMAF	8439	neutral sphingomyelinase (N-SMase) activation associated factor
FLJ25053	147463	ankyrin repeat domain 29
MGC33365	205428	hypothetical protein LOC205428
SUP	9709	homocysteine-inducible endoplasmic reticulum
ADH3	126	alcohol dehydrogenase 3 (class I), gamma polypeptide
UAP1	6675	sperm associated antigen 2
SLC16A1	6566	solute carrier family 16, member 1
ZNF450	9841	zinc finger protein 450
BTN3A3	10384	butyrophilin, subfamily 3, member A3
GNMT	27232	glycine N-methyltransferase
PDIR, FLJ30401	10954	for protein disulfide isomerase-related
NEDL2	57520	HECT, C2 and WW domain containing E3 ubiquitin protein ligase 2
SP8	221833	Sp8 transcription factor
PSP94	4477	seminal plasma beta-inhibin
SARG, FLJ3650	79098	specifically androgen-regulated protein
hK2	3817	kallikrein 2, prostatic
TNFAIP8	25816	tumor necrosis factor, alpha-induced protein 8
TACC2	10579	transforming, acidic coiled-coil containing protein 2
RAXLX	91464	intestine-specific homeobox
ORM2	5005	alpha-1-acid glycoprotein, type 2
hSgt2p	8496	protein-tyrosine phosphatase receptor-type f
DKFZp686M20	55973	B-cell receptor-associated protein 29
RACE	23600	alpha-methylacyl-CoA racemase
WS2D	6591	slug homolog, zinc finger protein
MGC9471	1491	homoserine dehydratase
TRPP8	79054	short form of the TRPM8 cationic channel
EST422562, AE	340273	P-glycoprotein ABCB5
GLUDP1	2747	glutamate dehydrogenase pseudogene 1
PTGER4	5734	prostaglandin E receptor 4, subtype EP4
MGC35403	254427	chromosome 10 open reading frame 47
hD52	7163	tumor protein D52
SLC35F2	54733	solute carrier family 35, member F2
c14_5270	122525	hypothetical protein LOC122525
bA526D8.1	23299	bicaudal D homolog 2
TYP	1846	serine/threonine specific protein phosphatase
WARS	7453	tryptophan tRNA ligase 1, cytoplasmic
MGC104194	54431	J-domain-containing protein disulfide isomerase-like protein
ZNF595	152687	zinc finger protein 595
MGC51750	476	Na+/K+ ATPase 1
SCRG1	11341	scrapie responsive protein 1
P450SCC	1583	steroid 20-22-lyase
PHAS-I	1978	phosphorylated heat- and acid-stable protein regulated by insulin 1

KLF4	9314	endothelial Kruppel-like zinc finger protein
FLJ35024	401491	FLJ35024
KIAA1851	29925	mannose-1-phosphate guanylyltransferase
Ptg-10	2289	rotamase
TTC39A	22996	tetratricopeptide repeat domain 39A
OACT2	129642	lysophospholipid acyltransferase
SWS	3977	CD118 antigen
PSPH	5723	L-3-phosphoserine phosphatase
SLC6A9	6536	solute carrier family 6 member 9
SDPII	11252	protein kinase C and casein kinase substrate in neurons 2
KRT8	3856	keratin, type II cytoskeletal 8
CLGN	1047	calmegin
SORD1	6652	sorbitol dehydrogenase
Q10, AGO2	27161	eukaryotic translation initiation factor 2C, 2
MGC111402	55784	multiple C2-domains with two transmembrane regions 2
UGT2B28	54490	UDP glycosyltransferase 2 family, polypeptide B28
LOC285463	285463	LOC285463
XAG-2, HAG-2,	10551	anterior gradient homolog 2 (Xenopus laevis)
testican-1	6695	testican-1
PTGFB	9518	PTGF-beta
ARF1	375	ADP-ribosylation factor 1
KIAA0746	23231	KIAA0746
MGC102891	400451	hypothetical protein LOC400451
Yo	1039	paraneoplastic cerebellar degeneration-associated antigen
MSTP049	4189	endoplasmic reticulum DnaJ homolog 4
IQGAP2	10788	IQ motif containing GTPase activating protein 2
nPKC-eta	5583	protein kinase C, eta
KIAA0032	8916	hect domain and RLD 3
RHOGAP3	1124	beta chimerin
SLC1A4	6509	solute carrier family 1, member 4
l1Rk3, l(1)-3Rk	79083	exophilin-3
PNLIP	5406	triacylglycerol acylhydrolase
bA101E13.1	51022	bA101E13.1 (GRX2 glutaredoxin (thioltransferase) 2)
STCRP	8614	STC-related protein
Ubc6p	51465	non-canonical ubquitin conjugating enzyme 1
TSC22D1	8848	transforming growth factor beta-stimulated protein TSC-22
PGM3	5238	phosphoglucomutase 3
KIAA0575	9687	GREB1
SHMT2	6472	serine hydroxymethyltransferase 2 (mitochondrial)
TCRGC2	445347	T-cell receptor gamma-chain constant region
STCH	6782	microsomal stress 70 protein ATPase core
MGC1947	128239	IQ motif containing GTPase activating protein 3
PEPCK2	5106	mitochondrial phosphoenolpyruvate carboxykinase 2
SLC45A3	85414	prostate cancer-associated gene 6
PTPN10	1843	dual specificity phosphatase 1
FLJ31715	152048	FLJ31715
dJ483K16.1	60481	homolog of yeast long chain polyunsaturated fatty acid

RAB3B	5865	Brain antigen RAB3B
hJAL	148327	cAMP responsive element binding protein 3-like 4
KIAA1822L	79802	KIAA1822-like
SLC2A10	81031	solute carrier family 2 member 10
MLL-MAML2	84441	mastermind-like 2
hK3	354	P-30 antigen
PRX-4	10549	thioredoxin peroxidase (antioxidant enzyme)
MGC88092	23052	endonuclease domain containing 1
WHIM	7852	leukocyte-derived seven-transmembrane-domain receptor
MGC125753	25874	brain protein 44
PGC	5225	Preprogastricsin
C9orf152	401546	chromosome 9 open reading frame 152
HEP27	10202	dehydrogenase/reductase member 2
CTBS	1486	chitobiase, di-N-acetyl-
beta4GalNAcT	55790	chondroitin beta1,4 N-acetylgalactosaminyltransferase
MGC31935	2342	farnesyltransferase, CAAX box, beta
PSD-93	1740	channel-associated protein of synapses, 110kDa
YLR066W	60559	signal peptidase complex subunit 3
SLC1A5	6510	neutral amino acid transporter B
EXTR2	2135	exostosin-like 2

Down-regulated genes in treated vs. untreated cells

Symbol	Entrez Id	Description
hCG_1815491	643911	hCG_1815491
TLL1	7092	tolloid-like 1
PTHRP, HHM, PLP, P	5744	parathyroid hormone-like protein
ADAMTS6	11174	a disintegrin and metalloproteinase with thrombospondin motifs 6
C21orf81	114035	chromosome 21 open reading frame 81
BARD1	580	BRCA1-associated RING domain gene 1
TRPS1	7227	zinc finger transcription factor TRPS1
SMA5	11042	SMA5
PAP	55	acid phosphatase, prostate
RIMS1	22999	regulating synaptic membrane exocytosis 1
HIST1H4B	8366	H4 histone family, member I
MGC9481	3790	potassium voltage-gated channel delayed-rectifier protein S3
hRCN3	11123	regulator of calcineurin 3
TUSP, KIAA1397	56995	tubby super-family protein
U2AF65	11338	U2 snRNP auxiliary factor large subunit
WLS	79971	putative NFkB activating protein 373
DKFZp761D112	84257	chromosome 8 open reading frame 57
PTPLA	9200	cementum attachment protein
hIAN2	474344	immune associated nucleotide 2
HOXC9	3225	homeo box C9
PRAC	84366	prostate, rectum and colon
RP11-35N6.1	54886	plasticity related gene 3
TSG7, SEN4, ETS7q,	7982	suppression of tumorigenicity 7
GRPP	2641	glucagon
hEndo	79694	mandaselin
SNK	10769	serum-inducible kinase
smMLCK	4638	myosin light chain kinase
NCAG1, FLJ11477	92126	chromosome 18 open reading frame 4
NFKBIZ	64332	nuclear factor of kappa light polypeptide gene enhancer in B-cells
neuroserpin	5274	serpin peptidase inhibitor, clade I (neuroserpin), member 1
HOXC13	3229	homeo box C13
SVIP	258010	SVIP
RDC1, GPR159	57007	G protein-coupled receptor
hCERK, FLJ23239, dA	64781	ceramide kinase
SEMA6D	80031	semaphorin 6D
psiNAIP	4671	psi neuronal apoptosis inhibitory protein
TSPAN7	7102	transmembrane 4 superfamily 2b
HOXC6	3223	homeobox C6
FLJ34534	1281	collagen, fetal
PRDM3	2122	oncogene EVI1
Orf3, GASZ, ALP1	136991	ankyrin repeat, SAM and basic leucine zipper domain containing 1
UGT2B17	7367	UDP glycosyltransferase 2 family, polypeptide B17
DKFZp781I035	1612	death-associated protein kinase 1
MAST4	375449	microtubule associated serine/threonine kinase family member 4
TGM3	7053	transglutaminase 3

efg	4602	c-myb13A_CDS
ZCWCC3	23515	MORC family CW-type zinc finger 3
STMY2	4319	matrix metallopeptidase 10 (stromelysin 2)
ZSIG13	11098	protease, serine, 23
Sp110	5376	growth arrest-specific 3
RDH10	157506	retinol dehydrogenase 10 (all-trans)
dJ1158H2.1	80258	EF-hand domain (C-terminal) containing 2
NFH	4744	neurofilament, heavy polypeptide 200kDa
ZBBX	79740	zinc finger, B-box domain containing
TEM8, FLJ21776, FLJ	84168	anthrax toxin receptor 1
sPLA2	5320	synovial phospholipase-A2
DKFZp761I1510	160851	diacylglycerol kinase, eta
nm23-H7	29922	non-metastatic cells 7
RARHOGAP	57569	RA and RhoGAP domain containing protein
MATN2	4147	matrilin 2
bA22L21.1	29078	hormone-regulated proliferation-associated 20 kDa protein
PLCB4	5332	phospholipase C beta 4
TINUR, NOT, RNR1, I	4929	transcriptionally inducible nuclear receptor related 1
MGC87039	440926	H3 histone, family 3A pseudogene
TYRO4	2042	ephrin receptor EphA3
FLJ25291	90355	hypothetical protein LOC90355
PLXNA2	5362	transmembrane protein OCT
LOC440087	440087	hypothetical protein LOC440087
NAB1	4664	NGFI-A binding protein 1
RZRA, ROR1, ROR2, I	6095	retinoic acid receptor-related orphan receptor alpha
NOV	4856	nephroblastoma overexpressed gene
PSGR2	143503	olfactory receptor OR11-15
MAGI1	154043	membrane associated guanylate kinase
VLGR1b	84059	G protein-coupled receptor 98
RBPMS	11030	RNA binding protein with multiple splicing
NAIP1B	728519	NAIP1B
RANBP3L	202151	RAN binding protein 3-like
TMEM144	55314	transmembrane protein 144
MGC1798	481	Na, K-ATPase beta-1 polypeptide
LRIG3	121227	leucine-rich repeats and immunoglobulin-like domains 3
TMEM34	55751	transmembrane protein 34
RFX3	5991	regulatory factor X, 3
LOC728160	728160	hypothetical LOC728160
SLC35F3	148641	solute carrier family 35, member F3
ZNF703	80139	zinc finger protein 703
MGC142015	3912	laminin, beta 1
CBLN2	147381	cerebellin 2
VEGI192A	9966	tumor necrosis factor (ligand) superfamily, member 15
RSS	2887	growth factor receptor-bound protein 10
FST	10468	follistatin
ZNFXY	4281	tripartite motif protein TRIM18
MGC131893	23242	cordon-bleu homolog

NZF1	23040	neural zinc finger transcription factor 1
FLJ37576	114899	complement-c1q tumor necrosis factor-related protein 3
PCDHY	27328	protocadherin X
TMEM23	259230	sphingomyelin synthase 1
SYND4, amphiglycar	6385	ryudocan amphiglycan
FLJ45803	399948	chromosome 11 open reading frame 92
TRIB1	10221	G-protein-coupled receptor induced protein
TSPAN8	7103	tetraspanin 8
ADDL	120	adducin-like protein 70
IRXB2	10265	iroquois homeobox 5
PPDIV	2737	GLI-Kruppel family member GLI3
VEPTP	5787	protein tyrosine phosphatase, receptor type, B
NLRR-1	57633	leucine rich repeat neuronal 1
NDUFB4	4710	NADH dehydrogenase (ubiquinone) 1 beta subcomplex
ZNF294	26046	zinc finger protein 294
DDC	1644	dopa decarboxylase (aromatic L-amino acid decarboxylase)
MT2A	4502	metallothionein 2A
KIAA1466	57612	KIAA1466
Slo3	157855	potassium channel, subfamily U, member 1
MRP2	57626	Mayven-related protein 2
ATPIS	23250	phospholipid-translocating ATPase
MGC61909	2591	GalNAc transferase 3
RCBTB2	1102	chromosome condensation 1-like
PRO1341	213	cell growth inhibiting protein 42
MCC1	4163	mutated in colorectal cancers
MGC99678	2852	IL8-related receptor
MGC15664	84517	actin related protein M1
MGC20446	220002	cytochrome b, ascorbate dependent 3
LOC644914	644914	similar to H3 histone, family 3B
UGT8	7368	UDP glycosyltransferase 8
LOC647595	647595	LOC647595
bA386N14.2	7403	ubiquitously transcribed tetratricopeptide repeat, X chromosome
SI	6476	Oligosaccharide alpha-1,6-glucosidase
MGC105131	10144	family with sequence similarity 13, member A1
LOC120376	120376	hypothetical protein LOC120376
RIP-1	11103	HIV-1 rev binding protein 2
RP11-378J18.7	64853	axin interactor, dorsalization associated
RTN1	6252	reticulon 1
MGC130023	90161	heparan sulfate 6-O-sulfotransferase 2
bA307O14.2	93663	Rho GTPase activating protein 18
SLC44A1	23446	CDW92 antigen
KLHDC8A	55220	kelch domain containing 8A
MCTP1	79772	multiple C2-domains with two transmembrane regions 1
PMS2CL	441194	PMS2 C-terminal like pseudogene
ZNF680	340252	zinc finger protein 680
TLIMP	57561	thioredoxin-binding protein-2-like inducible membrane
NTS1	4922	neurotensin

SEST1, PA26	27244	sestrin 1
O/E-2	253738	early B-cell factor 3
LOC728431	728431	hypothetical LOC728431
IRX3	79191	iroquois homeobox protein 3
PLCeta1	23007	phospholipase C-like 3
TCF1ALPHA	51176	lymphoid enhancer binding factor-1
NCAM21, MGC5100	4685	neural cell adhesion molecule 2
MGC15604	131034	copine IV
PTPRR	5801	protein-tyrosine phosphatase NC-PTPCOM1
TRPM2	1191	testosterone-repressed prostate message 2
RP11-255E17.1	9411	Rho GTPase activating protein 29
HOXA13	3209	homeo box 1J
PRKD1	5587	protein kinase D1
PSP24B	81491	brain expressed G-protein-coupled receptor PSP24 beta
STXBP5L	9515	tomosyn-like
VTA1	51534	chromosome 6 open reading frame 55
fibroglycan, SYND2	6383	heparan sulfate proteoglycan core protein
ZIC2	7546	zinc finger protein of the cerebellum 2
FLJ38295	388815	hypothetical protein LOC388815
MGC3032	65998	MGC3032
HVH3	1847	dual specificity phosphatase 5
VIGR	57211	developmentally regulated G-protein-coupled receptor
LOC728555	728555	LOC728555
PTGER3	5733	prostaglandin receptor (PGE-2)
STK3	6788	serine/threonine kinase 3 (Ste20, yeast homolog)
E1	590	butyrylcholinesterase
GUSBP1	153561	glucuronidase, beta pseudogene 1
RFTN1	23180	proliferation-inducing protein 10
FADS8	123099	sphingolipid delta 4 desaturase/C-4 hydroxylase
KIAA1890	64478	CUB and Sushi multiple domains 1
SLC12A2	6558	solute carrier family 12
DEGA	347902	amphoterin induced gene 2
VR20	60437	cadherin-like protein VR20
MGC29633	3495	immunoglobulin heavy constant delta
ANKRD20A2	441430	ankyrin repeat domain 20 family, member A2
ROC2	6014	Ric-like, expressed in neurons
LOC643187	643187	LOC643187
NUDT4	11163	diphosphoinositol polyphosphate phosphohydrolase type 2
OTTHUMG0000006(	728747	ankyrin repeat domain 20 family, member A4
SLITRK3	22865	slit and trk like gene 3
NUDT4P1	440672	nudix (nucleoside diphosphate linked moiety X)
LOC727770	727770	LOC727770
DKFZp434A171	84210	ankyrin repeat domain 20A
PCDHY	83259	protocadherin Y
LOC727762	727762	similar to NADH:ubiquinone oxidoreductase B15 subunit
SPAG9	9043	sperm surface protein
OPRK1	4986	opioid receptor, kappa 1

b55C20.2	11039	SMA4
mGCP	2346	prostate specific membrane antigen variant F
Kir3.1, GIRK1, KGA	3760	G protein-activated inward rectifier potassium channel 1
dJ718P11.1, FLJ1111	55304	serine palmitoyltransferase, long chain base subunit 3
MGC104684	1487	C-terminal binding protein 1
UG0636c06	84698	calcyphosphine 2
hdhc9	1769	dynein, axonemal, heavy chain 8
PSCTK3	660	BMX non-receptor tyrosine kinase
KAO	26	kidney amine oxidase
PMSL2	5395	postmeiotic segregation increased (S. cerevisiae) 2
SSBP2	23635	single-stranded DNA binding protein 2
OSR2	116039	odd-skipped related 2 (Drosophila)
FLJ38155, DKFZp434	51313	hypothetical protein LOC51313
TDAG51, DT1P1B11,	22822	proline-histidine rich protein
ARG1	383	arginase, type I
LOC286367	286367	LOC286367
VIA	57556	sema domain, transmembrane domain (TM), and cytoplasmic domain
LOH11CR1D	2230	adrenodoxin
SYTL2	54843	exophilin-4
hWNT5A	7474	wingless-type MMTV integration site family, member 5A
bA12D24.3.2	6013	prorelaxin
SPG31	65055	receptor expression enhancing protein 1
DKFZp547I1415	90141	CG11165-like
p55-GAMMA	8503	phosphoinositide-3-kinase, regulatory subunit
MGC10430	3557	intracellular IL-1 receptor antagonist type II
UNRIP, pt-wd, MAW	11171	unr-interacting protein
IGFBP3	3486	binding protein 53
ANKRD20A3	441425	ankyrin repeat domain 20 family, member A3
SOBP	55084	sine oculis binding protein homolog (Drosophila)
MIR1	83856	fibronectin type III and SPRY domain containing 1-like
TIMP2	7077	tissue inhibitor of metalloproteinases 2
MGC105115	1290	type V preprocollagen alpha 2 chain
TUBA3, B-ALPHA-1,	7846	tubulin, alpha 3
FIGNL1	63979	fidgetin-like 1
LOC149478	149478	hypothetical protein LOC149478
MGC34732, FLJ4211	220047	coiled-coil domain containing 83
NETO1	81832	neuropilin- and tolloid-like protein 1
RP11-401M16.1	55450	calcium/calmodulin-dependent protein kinase II inhibitor 1
pro-NRG3	10718	neuregulin-3-like polypeptide
hRCN2	10231	Down syndrome critical region gene 1-like 1
ANXA9	8416	annexin XXXI
MGC87783	3020	H3 histone, family 3A
TGD	19	ATP binding cassette transporter 1
PRKACN2	5570	PKI-beta
maba1, EIG121	57535	hypothetical protein LOC57535
GRB14	2888	growth factor receptor-bound protein 14
ST8SIA4	7903	sialyltransferase 8 (alpha-2, 8-polysialytransferase) D

MAN9	4121	Man9-mannosidase
SLC25A18	83733	mitochondrial glutamate carrier 2
LOC653436	653436	LOC653436
dJ495O3.1	84969	chromosome 20 open reading frame 100
LOC283666	283666	LOC283666
DKFZP434N1511	54522	ankyrin repeat domain 16
bA12D24.1.2	6019	prorelaxin H2
LAP2	3320	heat shock protein 90kDa alpha (cytosolic), class A member 1
RGAG2	10687	onconeuronal antigen MA2
MGC126075	1024	CDK8 protein kinase
UGT2B8	7366	UDP-glucuronosyltransferase UGT2B15
NR3C1	2908	glucocorticoid receptor
GLCLC, GLCL	2729	gamma-glutamylcysteine synthetase
RAI2	10742	retinoic acid induced 2
RPS15P6	391845	ribosomal protein S15 pseudogene 6
TAFA2	338811	family with sequence similarity 19
NAPEPLD	222236	N-acyl-phosphatidylethanolamine-hydrolyzing phospholipase D
SRCL, CL-P1, SCARA4	81035	scavenger receptor class A, member 4
bA219P18.1	55151	transmembrane protein 38B
NDP	4693	norrin
TASSC	25934	nipsnap homolog 3A
SCGB1D2	10647	lipophilin B (uteroglobin family member), prostatein-like
LOC644339	644339	LOC644339
mlklak	51776	leucine zipper- and sterile alpha motif-containing kinase

Androgen up-regulated proteins from iTRAQ study

**Symbol**	**Entrez_ID**	**Description**
STAU1	6780	Isoform Long of Double-stranded RNA-binding protein
EIF3M	10480	Eukaryotic translation initiation factor 3, subunit M
UAP1	6675	Isoform AGX2 of UDP-N-acetylhexosamine pyrophosphorylase
ENDOD1	23052	Endonuclease domain-containing 1 protein precursor
SHMT1	6470	Serine hydroxymethyltransferase, cytosolic
MAP2K2	5605	Dual specificity mitogen-activated protein kinase kinase 2
HEBP2	23593	Isoform 2 of Heme-binding protein 2
PYGB	5834	Glycogen phosphorylase, brain form
SSRP1	6749	FACT complex subunit SSRP1
RPS10	6204	Ribosomal protein S10
PABPC1	26986	Isoform 1 of Polyadenylate-binding protein 1
ACBD3	64746	Golgi resident protein GCP60
DBI	1622	Diazepam binding inhibitor
LCP1	3936	Plastin-2
KLK3	354	Prostate-specific antigen precursor
RPS3	6188	40S ribosomal protein S3
PSMD3	5709	26S proteasome non-ATPase regulatory subunit 3
AHCY	191	Adenosylhomocysteinase
PEA15	8682	Astrocytic phosphoprotein PEA-15
PTGES3	10728	Prostaglandin E synthase 3
SEC23B	10483	Protein transport protein Sec23B
NAP1L4	4676	Nucleosome assembly protein 1-like 4
FDFT1	2222	Squalene synthetase
ACLY	47	ATP-citrate synthase
UBE2L3	7332	Ubiquitin-conjugating enzyme E2 L3
NDRG1	10397	N-myc downstream regulated gene 1
CAPNS1	826	Calpain small subunit 1
CDC2	983	Cell division cycle 2
FASN	2194	Fatty acid synthase
AARS	16	Alanyl-tRNA synthetase, cytoplasmic
RPL7	6129	60S ribosomal protein L7
AHSA1	10598	Activator of 90 kDa heat shock protein ATPase homolog 1
DIAPH1	1729	Protein diaphanous homolog 1
ACSL3	2181	Long-chain-fatty-acid--CoA ligase 3
CSDA	8531	Cold shock domain protein A
FARSLA	2193	Phenylalanyl-tRNA synthetase alpha chain
RPL10L	140801	60S ribosomal protein L10-like
SND1	27044	Staphylococcal nuclease domain-containing protein 1
GPS1	2873	G protein pathway suppressor 1 isoform 2
RPS26	6231	Ribosomal protein S26
PSMD12	5718	26S proteasome non-ATPase regulatory subunit 12
EEF2	1938	Elongation factor 2
RPL38	6169	60S ribosomal protein L38
SORD	6652	Sorbitol dehydrogenase
GTPBP9	29789	Isoform 2 of Putative GTP-binding protein 9

TXN	7295	Thioredoxin
RPS8	6202	40S ribosomal protein S8
FKBP5	2289	FK506-binding protein 5
PRDX6	9588	Peroxiredoxin-6
YWHAG	7532	14-3-3 protein gamma
RPS16	6217	40S ribosomal protein S16
ACAT2	39	Acetyl-CoA acetyltransferase, cytosolic
SRM	6723	Spermidine synthase
WARS	7453	Tryptophanyl-tRNA synthetase, cytoplasmic
COPA	1314	Coatomer subunit alpha
SIL1	64374	Nucleotide exchange factor SIL1 precursor
NAT10	55226	N-acetyltransferase 10
SEC31A	22872	SEC31 homolog A isoform 4
NARS	4677	Asparaginyl-tRNA synthetase, cytoplasmic
TARS	6897	Threonyl-tRNA synthetase
C18orf32	497661	Chromosome 18 open reading frame 32
LOC38984[illegible]	389842	Similar to Ran-specific GTPase-activating protein
SERBP1	26135	Plasminogen activator inhibitor 1 RNA-binding protein
NOL5A	10528	Nucleolar protein Nop56
SERPINB6	5269	Serpin peptidase inhibitor, clade B, member 6
GARS	2617	Glycyl-tRNA synthetase
PGM2	55276	Phosphoglucomutase-2
ASNS	440	Asparagine synthetase
TXNL5	84817	Thioredoxin-like protein 5
LOC64481[illegible]	644816	Similar to Heat shock protein HSP 90-beta

Androgen down-regulated proteins from iTRAQ study

Symbol	Entrez_ID	Description
HLA-C	3107	HLA class I histocompatibility antigen
SNRPA	6626	U1 small nuclear ribonucleoprotein A
PDCD6IP	10015	PDCD6IP protein
TIMM8A	1678	Mitochondrial import inner membrane translocase
NCAM2	4685	Neural cell adhesion molecule 2 precursor
DPP7	29952	Dipeptidyl-peptidase 2 precursor
AK3	50808	GTP:AMP phosphotransferase mitochondrial
CTSD	1509	Cathepsin D precursor
FOLH1	2346	Isoform PSMA-1 of Glutamate carboxypeptidase 2
FLOT1	10211	Flotillin-1
DDB1	1642	DNA damage-binding protein 1
MME	4311	Neprilysin
NDUFA8	4702	NADH dehydrogenase [ubiquinone]
WDR57	9410	WD repeat protein 57
C11orf58	10944	Small acidic protein
FXR2	9513	Fragile X mental retardation syndrome-related protein 2
RNASET2	8635	Isoform 1 of Ribonuclease T2 precursor
SF3A3	10946	Splicing factor 3A subunit 3
MIA3	375056	similar to melanoma inhibitory activity 3 isoform 1
MAP1B	4131	Microtubule-associated protein 1B
TRA2A	29896	Isoform Long of Transformer-2 protein homolog
ATP1B1	481	Isoform 1 of Sodium\potassium-transporting
PTMS	5763	Parathymosin
ALDH2	217	Aldehyde dehydrogenase, mitochondrial precursor
HDGF	3068	Hepatoma-derived growth factor
HNRPL	3191	heterogeneous nuclear ribonucleoprotein L isoform a
SUCLG1	8802	succinate-CoA ligase, GDP-forming, alpha subunit
SNRPE	6635	Small nuclear ribonucleoprotein E
TM7SF2	7108	Isoform 1 of Delta(14)-sterol reductase
ATP5J	522	ATP synthase coupling factor 6, mitochondrial precursor
SFRS1	6426	splicing factor, arginine\serine-rich 1
SFPQ	6421	Isoform Long of Splicing factor
LGMN	5641	Legumain precursor
CTNND1	1500	Isoform 1AB of Catenin delta-1
PAFAH1B2	5049	Platelet-activating factor acetylhydrolase IB subunit beta
NHP2L1	4809	NHP2-like protein 1
NONO	4841	Non-POU domain-containing octamer-binding protein
FUS	2521	Isoform Short of RNA-binding protein FUS
DNAJB11	51726	DnaJ homolog subfamily B member 11 precursor
