# Supplementary material for: “Topological Significance” Analysis of Gene Expression and Proteomic Profiles from Prostate Cancer Cells Reveals Key Mechanisms of Androgen Response
Source: PLoS One. 2010 Jun 3;5(6):e10936. doi: 10.1371/journal.pone.0010936 (PMC2880599; doi:10.1371/journal.pone.0010936)
Supplement: Table S2 — List of topologically significant genes and proteins determined from gene expression and proteomics data. The list of up-regulated genes and proteins were submitted to the scoring procedure separately, resulting in two sets of topologically significant regulatory proteins. We identified 962 topologically significant proteins from gene expression data and 577 topologically significant proteins from proteomic data (FDR<5%). (0.52 MB PDF) [file pone.0010936.s002.pdf]

Topologically significant genes determined from gene expression data

Symbol	Entrez_ID	Description	p value	percentile
AAG11	2274	aging-associated gene 11	2.24E-19	99.89605
CLS	6197	ribosomal protein S6 kinase, 90kDa, polypeptide 3	1.51E-18	99.84407
CTNNB	1499	catenin (cadherin-associated protein), beta 1, 88kDa	5.45E-17	99.74012
CCND2	894	cyclin D2	3.74E-16	99.63617
FLJ21396	23132	RAD54-like 2	5.26E-16	99.53222
AIS	367	androgen receptor	1.36E-15	99.42827
DAB2	1601	mitogen-responsive phosphoprotein	1.55E-15	99.32432
ABP-280	2316	filamin A, alpha	3.89E-15	99.22037
ACH	2261	fibroblast growth factor receptor 3	1.2E-14	99.11642
RAD9	5883	RAD9 homolog	1.31E-14	98.9605
FLJ40346	144715	RAD9 homolog B	1.31E-14	98.9605
CMT2F	3315	heat shock 27kDa protein 1	1.49E-14	98.80457
FLJ25949	23081	jumonji domain containing 2C	3.62E-14	98.64865
AADC	1644	dopa decarboxylase (aromatic L-amino acid decarboxylase)	3.62E-14	98.64865
DKFZp313L	2934	gelsolin (amyloidosis, Finnish type)	3.62E-14	98.49272
AP-1	3725	v-jun avian sarcoma virus 17 oncogene homolog	8.58E-14	98.38877
ARH	26119	LDL receptor adaptor protein	9.98E-14	98.23285
CHC	1213	clathrin, heavy chain (Hc)	9.98E-14	98.23285
CD49D	3676	integrin alpha 4	1.19E-13	98.02495
CD29	3688	integrin beta 1	1.19E-13	98.02495
DBK	5585	serine-threonine kinase N	1.24E-13	97.86902
DPC4	4089	mothers against decapentaplegic homolog 4	1.48E-13	97.76507
PAB1	26986	poly(A) binding protein, cytoplasmic 1	1.49E-13	97.66112
HBO1	11143	MYST histone acetyltransferase 2	1.58E-13	97.5052
DSAP1	9733	squamous cell carcinoma antigen recognized by T cells 3	1.58E-13	97.5052
COASTER	26036	coactivator for steroid receptors	1.89E-13	97.34927
ARA24	5901	ras-related nuclear protein	2.01E-13	97.19335
ITGB7	3695	integrin, beta 7	2.01E-13	97.19335
ITGA8	8516	integrin, alpha 8	2.17E-13	96.8815
HsT18964	22801	integrin, alpha 11	2.17E-13	96.8815
ITGA10	8515	integrin, alpha 10	2.17E-13	96.8815
CD49C	3675	very late activation protein 3 receptor, alpha-3 subunit	2.17E-13	96.8815
CCNE	898	cyclin Es	2.39E-13	96.20582
RBAK	57786	RB-associated KRAB zinc finger	2.39E-13	96.20582
POD1	6943	transcription factor 21	2.39E-13	96.20582
TSG10	7251	tumor susceptibility gene 101	2.39E-13	96.20582
HKMT1069	79084	methylosome protein 50	2.39E-13	96.20582
BAF57	6605	mammalian chromatin remodeling complex BRG1	2.39E-13	96.20582
CTC75	2547	ATP-dependent DNA helicase II, 70 kDa subunit	2.39E-13	96.20582
ANT-1	24148	U5 snRNP-associated 102 kDa protein	2.39E-13	96.20582
PAR4	5074	WT1-interacting protein	2.39E-13	96.20582
ACK	10188	tyrosine kinase, non-receptor, 2	2.57E-13	95.68607
IFI16	3428	interferon-gamma induced protein IFI 16	3.33E-13	95.58212
DOD	5300	protein (peptidyl-prolyl cis/trans isomerase)	3.59E-13	95.47817
AAG4	1191	testosterone-repressed prostate message 2	4.31E-13	95.37422

HSP75	10131	heat shock protein 75	4.41E-13	95.11435
D6S182	3326	heat shock 90kDa protein 1, beta	4.41E-13	95.11435
ECGP	7184	heat shock protein 90kDa beta, member 1	4.41E-13	95.11435
FLJ31884	3320	heat shock protein 90kDa alpha (cytosolic)	4.41E-13	95.11435
FLJ31165	57610	RAN binding protein 10	5.53E-13	94.85447
GSK3B	2932	glycogen synthase kinase 3 beta	6.05E-13	94.75052
HsMAD1	8379	tumor protein p53 inducible protein 9	6.95E-13	94.64657
ARA160	7110	TATA element modulatory factor 1	7.32E-13	94.49064
FLJ39089	7520	Ku86 autoantigen related protein 1	7.32E-13	94.49064
GGA1	26088	gamma-adaptin related protein 1	9.55E-13	94.33472
HIP1	3092	huntingtin interacting protein 1	9.8E-13	94.23077
G3PD	2597	glyceraldehyde 3-phosphate dehydrogenase	1.04E-12	94.12682
ALPHA-RLC	3680	integrin, alpha 4-like	1.23E-12	94.02287
BGP	634	biliary glycoprotein adhesion molecule	1.39E-12	93.91892
CALR	811	Sicca syndrome antigen A (autoantigen Ro; calreticulin)	1.46E-12	93.65904
C1Q-C	714	complement component 1, q subcomponent, C chain	1.46E-12	93.65904
C1QB	713	complement component 1, q subcomponent	1.46E-12	93.65904
C1QA	712	complement component C1q, A chain	1.46E-12	93.65904
ELAV1	1994	Hu antigen R	1.67E-12	93.39917
MER	10461	STK kinase	1.67E-12	93.29522
CASP1	834	caspase 1	1.67E-12	93.19127
DKFZP586J	51564	histone deacetylase 7A	1.74E-12	93.08732
KIAA0093	4734	neural precursor cell expressed	1.86E-12	92.98337
AMPK	5563	AMP-activated protein kinase alpha 2 catalytic subunit	2.01E-12	92.82744
AMPK	5562	AMPK alpha 1	2.01E-12	92.82744
INPP5F	4952	oculocerebrorenal syndrome of Lowe	2.51E-12	92.67152
CSBP1	1432	stress-activated protein kinase 2A	2.56E-12	92.56757
BRCA2	675	breast cancer susceptibility protein BRCA2	2.59E-12	92.46362
CAV	857	caveolin 1 caveolae protein, 22kD	2.9E-12	92.35967
CCND3	896	G1/S-specific cyclin D3	3E-12	92.25572
APOB	338	apolipoprotein B48	3.16E-12	92.15177
FLJ25220	3679	integrin alpha 7	3.68E-12	92.04782
SENP1	29843	SUMO1/sentrin specific protease 1	4.32E-12	91.94387
ADPRT	142	poly (ADP-ribose) polymerase family, member 1	5.98E-12	91.83992
CALM2	805	LP7057 protein	6.3E-12	91.63202
CALM1	801	calmodulin 1 (phosphorylase kinase, delta)	6.3E-12	91.63202
CALM3	808	calmodulin 3 (phosphorylase kinase, delta)	6.3E-12	91.63202
B-MYB	4605	v-myb avian myeloblastosis viral oncogene homolog-like 2	6.7E-12	91.42412
CHC1	1104	regulator of chromosome condensation 1	6.99E-12	91.32017
APRF	6774	DNA-binding protein APRF	1.08E-11	91.21622
DJ-1	11315	protein DJ-1	1.43E-11	91.11227
DKFZp686H	3065	histone deacetylase 1	1.44E-11	91.00832
AOF2	23028	lysine (K)-specific demethylase 1	1.44E-11	90.90437
DKFZp781I	4914	neurotrophic tyrosine kinase, receptor, type 1	1.64E-11	90.80042
ARA55	7041	transforming growth factor beta 1 induced transcript 1	1.85E-11	90.69647
COLEC1	4153	mannose-binding protein C	1.85E-11	90.59252
AZAMP	566	cationic antimicrobial protein 37	2.41E-11	90.48857

AMCBX1	8517	incontinentia pigmenti	2.72E-11	90.38462
HIRS-1	3667	insulin receptor substrate 1	2.82E-11	90.28067
DKFZp686C	5796	protein tyrosine phosphatase, receptor type, K	3E-11	90.17672
BFGF	2247	prostatropin	3.02E-11	90.07277
CD340	2064	v-erb-b2 avian erythroblastic leukemia viral oncogene	3.51E-11	89.96881
AP-1	2353	cellular oncogene c-fos	3.88E-11	89.86486
FINB	6239	zinc finger motif enhancer binding protein 1	4.17E-11	89.76091
CDC37	11140	cell division cycle 37 protein	4.47E-11	89.65696
FLJ23903	4641	nuclear myosin I	4.65E-11	89.55301
CDC2	983	cell cycle controller CDC2	4.8E-11	89.44906
BGN	633	biglycan proteoglycan	5.41E-11	89.34511
ACLS	2737	GLI-Kruppel family member GLI3	6.32E-11	89.24116
MSP23	5052	peroxiredoxin 1	6.43E-11	89.13721
AF6q21	2309	forkhead, Drosophila	6.83E-11	89.03326
FOXA1	3169	hepatocyte nuclear factor 3, alpha	8.46E-11	88.92931
DDR2	4921	cell migration-inducing protein 20	8.64E-11	88.77339
ASV	6714	tyrosine kinase pp60c-src	8.64E-11	88.77339
ALPS2B	841	cysteine protease	9.82E-11	88.61746
BAG1	573	BCL2-associated athanogene	1E-10	88.51351
Cmyb	4602	c-myb13A_CDS	1.01E-10	88.40956
INSRR	3645	IR-related receptor	1.11E-10	88.30561
FLJ20408	3280	transcription factor HES-1	1.16E-10	88.20166
STAT5	6777	transcription factor STAT5B	1.16E-10	88.09771
ARNIP	25898	androgen-receptor N-terminal-interacting protein	1.2E-10	87.99376
DKFZp761F	221037	jumonji domain containing 1C	1.38E-10	87.88981
FKBP5	2289	rotamase	1.4E-10	87.78586
MYC	4609	myc proto-oncogene protein	1.8E-10	87.68191
CSBP	3190	transformation upregulated nuclear protein	3.09E-10	87.57796
CANP	823	calpain 1, large subunit	3.26E-10	87.47401
OSRC	5925	retinoblastoma-1	3.4E-10	87.37006
BSP1	4086	MAD, mothers against decapentaplegic homolog 1	3.79E-10	87.26611
BR	3673	platelet antigen Br	4.15E-10	87.16216
BAF155	6599	chromatin remodeling complex BAF155 subunit	4.18E-10	87.05821
AKT3	10000	v-akt murine thymoma viral oncogene homolog 3	4.24E-10	86.85031
AKT	207	protein kinase B	4.24E-10	86.85031
AKT2	208	v-akt murine thymoma viral oncogene homolog 2	4.24E-10	86.85031
ARH6	388	oncogene RHO H6	4.32E-10	86.64241
SRY	6736	testis-determining factor	4.61E-10	86.53846
C4orf1	10463	chromosome 4 open reading frame 1	4.69E-10	86.43451
CREB	1385	cAMP responsive element binding protein 1	4.82E-10	86.33056
FLJ26459	5934	retinoblastoma-like 2 (p130)	5.75E-10	86.22661
C/EBP-alph	1050	CCAAT/enhancer binding protein alpha	5.86E-10	86.12266
AC068139.	6435	surfactant, pulmonary-associated protein A1B	6.13E-10	86.01871
FH	3949	low density lipoprotein receptor	7.47E-10	85.91476
CD49e	3678	integrin, alpha 5 (fibronectin receptor, alpha polypeptide)	7.96E-10	85.81081
ASK1	4217	MAPK/ERK kinase kinase 5	8.3E-10	85.70686
HPTPZ	5803	protein tyrosine phosphatase, receptor-type	1.04E-09	85.60291

A2MR	4035	type V tgf-beta receptor	1.19E-09	85.49896
AWD	4830	NDP kinase A	1.45E-09	85.39501
ARH12	387	oncogene RHO H12	1.46E-09	85.29106
ARA70	8031	nuclear receptor coactivator 4	1.5E-09	85.18711
DIAPH3	81624	diaphanous homolog 3	1.51E-09	85.08316
MAP3K7	6885	transforming growth factor-beta-activated kinase 1	1.55E-09	84.97921
FLJ13161	26524	serine/threonine kinase KPM	1.58E-09	84.87526
MAX	4149	MAX protein	1.85E-09	84.77131
CR	6997	teratocarcinoma-derived growth factor 1	1.94E-09	84.66736
DKFZp686A	3146	high-mobility group (nonhistone chromosomal) protein 1	1.98E-09	84.51143
HMG2	3148	high-mobility group box 2	1.98E-09	84.51143
CKBBP2	90480	papillomavirus L2 interacting nuclear protein 1	2.04E-09	84.35551
FLJ16239	10013	histone deacetylase 6	2.38E-09	84.25156
DKFZp434K	7442	transient receptor potential vanilloid 1b	2.43E-09	84.14761
KIAA0988	6904	tubulin-specific chaperone d	2.49E-09	84.04366
BID	637	BID isoform Si6	2.65E-09	83.93971
ON	6678	secreted protein, acidic, cysteine-rich	2.72E-09	83.83576
DLG4	1742	synapse-associated protein 90	2.98E-09	83.73181
HMX3	27043	proline and glutamic acid rich nuclear protein	3.18E-09	83.62786
RAB6B	51560	small GTP-binding protein	3.25E-09	83.47193
RAB6	5870	RAB6, member RAS oncogene family	3.25E-09	83.47193
BCL1	595	G1/S-specific cyclin D1	3.35E-09	83.31601
DJBP	64800	EF-hand calcium binding domain 6	3.47E-09	83.21206
E2F-1	1869	retinoblastoma-associated protein 1	3.98E-09	83.10811
ART-27	8409	ubiquitously-expressed transcript	4.17E-09	83.00416
DDX5	1655	DEAD (Asp-Glu-Ala-Asp) box polypeptide 5	4.37E-09	82.90021
BHLHb31	23462	hairy-related transcription factor 1	4.83E-09	82.79626
FKHL13	2302	forkhead box J1	4.89E-09	82.69231
FLJ26504	6464	SHC transforming protein 1	5.05E-09	82.58836
PLK	5347	polo like kinase	5.65E-09	82.48441
CD49a	3672	very late activation protein 1	5.75E-09	82.38046
SNAPAP	23557	SNARE associated protein snapin	6.17E-09	82.27651
MGC10268	9063	protein inhibitor of activated STAT X	6.58E-09	82.17256
CAF	8850	CREBBP-associated factor	7.03E-09	82.06861
BETA-TRCP	8945	beta-TrCP1	7.36E-09	81.96466
ATF4	468	activating transcription factor 4	8.76E-09	81.86071
HIF-1alpha	3091	member of PAS superfamily 1	8.78E-09	81.75676
RAC3	5881	ras-related C3 botulinum toxin substrate 3	8.83E-09	81.65281
CMTDI1	1785	cytoskeletal protein	9.19E-09	81.54886
ADRB2	154	catecholamine receptor	9.99E-09	81.44491
TOP2	7153	DNA topoisomerase II, alpha isozyme	1.04E-08	81.34096
BCL2	596	B-cell CLL/lymphoma 2	1.05E-08	81.23701
DAPK3	1613	ZIP kinase isoform	1.13E-08	81.13306
MGC786	5518	protein phosphatase 2 (formerly 2A), alpha isoform	1.13E-08	81.02911
DKFZp686C	4790	nuclear factor kappa-B, subunit 1	1.25E-08	80.92516
CNK	10256	connector enhancer of kinase suppressor of Ras 1	1.26E-08	80.82121
HPE4	7050	transforming growth factor-beta-induced factor	1.27E-08	80.71726

CRS2	4488	msh homeobox 2	1.48E-08	80.61331
GNA-11	2767	guanine nucleotide-binding protein, Gq class, GNA11	1.53E-08	80.45738
G-ALPHA-q	2776	guanine nucleotide binding protein (G protein)	1.53E-08	80.45738
GPIPLD	2822	GPI-specific phospholipase D	1.76E-08	80.30146
GLI2	2736	zinc finger protein GLI2	1.86E-08	80.19751
CHNG1	7253	thyroid stimulating hormone receptor	1.89E-08	80.09356
GLI	2735	glioma-associated oncogene homolog (zinc finger protein)	2.02E-08	79.9896
BSAP	5079	B-cell lineage specific activator	2.15E-08	79.88565
NR2C1	7181	orphan nuclear receptor TR2	2.33E-08	79.7817
ARR3	407	cone arrestin	2.36E-08	79.67775
D12S1644	6778	signal transducer and activator of transcription 6	2.38E-08	79.5738
DELTA1	28514	delta-like 1	2.58E-08	79.46985
BZS	5728	MMAC1 phosphatase and tensin homolog	2.71E-08	79.3659
C-Rel	5966	v-rel reticuloendotheliosis viral oncogene homolog	2.8E-08	79.26195
PAK5	56924	p21-activated protein kinase 6	2.92E-08	79.158
CD61	3690	integrin, beta 3 (platelet glycoprotein IIIa, antigen CD61)	3.12E-08	79.05405
HSSOX6	55553	SRY-box containing gene 6	3.13E-08	78.9501
ACTRIB	91	serine(threonine) protein kinase receptor R2	3.24E-08	78.84615
MAP3K11	4296	mitogen-activated protein kinase kinase kinase 11	3.48E-08	78.7422
CDC25B	994	cell division cycle 25 homolog B	3.75E-08	78.63825
AT225	1958	zinc finger protein 225	3.83E-08	78.5343
PRO1280	51684	suppressor of fused homolog (Drosophila)	4.05E-08	78.43035
MGC13177	5970	v-rel avian reticuloendotheliosis viral oncogene homolog A	4.1E-08	78.3264
MAGI-3	260425	membrane-associated guanylate kinase-related 3	4.24E-08	78.22245
PI5	5268	serpin peptidase inhibitor, clade B (ovalbumin), member 5	4.28E-08	78.1185
ACVRLK3	657	bone morphogenetic protein receptor, type IA	4.33E-08	78.01455
ANFH	1280	collagen II, alpha-1 polypeptide	4.59E-08	77.9106
FAST-1	8928	Human homolog of Xenopus forkhead activin	4.7E-08	77.80665
MMP-X1	4323	membrane-type matrix metalloproteinase 1	5.28E-08	77.7027
Bp50	958	CD40 type II isoform	5.45E-08	77.59875
DKFZp781D	7018	transferrin	5.46E-08	77.44283
SREBF1	6720	sterol regulatory element binding transcription factor 1	5.46E-08	77.44283
N-syndecar	9672	syndecan proteoglycan 3	5.52E-08	77.2869
PKC-MU	5587	protein kinase D1	5.54E-08	77.18295
CLTA	1211	clathrin, light polypeptide A	5.62E-08	77.02703
CLTB	1212	clathrin, light polypeptide	5.62E-08	77.02703
CD28	940	CD28 antigen (Tp44)	6.24E-08	76.8711
AAT5	7046	transforming growth factor beta receptor I	6.6E-08	76.76715
BCAR1	9564	Cas scaffolding protein family member 1	6.87E-08	76.6632
MGC:3310	7189	TNF receptor-associated factor 6	6.95E-08	76.55925
FOG	161882	friend of GATA-1	7.21E-08	76.4553
ASCR	5621	prion protein	7.47E-08	76.35135
C-Kit	3815	mast/stem cell growth factor receptor	7.58E-08	76.2474
CRCS3	4092	SMAD family member 7	7.82E-08	76.14345
CD44	960	chondroitin sulfate proteoglycan 8	8.87E-08	76.0395
BRCA1	672	breast and ovarian cancer susceptibility protein 1	1.04E-07	75.93555
MGC12632	4593	skeletal muscle receptor tyrosine kinase	1.04E-07	75.77963

KIAA0816	4038	low-density lipoprotein receptor-related protein 4	1.04E-07	75.77963
BNSP	6696	SPP1/CALPHA1 fusion	1.06E-07	75.6237
CIS1	8651	STAT induced SH3 protein 1	1.07E-07	75.51975
AXL	558	AXL transforming sequence/gene	1.08E-07	75.4158
PS6K	6198	serine/threonine kinase 14 alpha	1.14E-07	75.31185
CD126	3570	interleukin 6 receptor alpha subunit	1.15E-07	75.15593
CD130	3572	CD130 antigen	1.15E-07	75.15593
BLIMP1	639	beta-interferon gene positive-regulatory domain I binding	1.29E-07	75
FADD	8772	Fas-associating death domain-containing protein	1.3E-07	74.89605
ADABP	1803	dipeptidylpeptidase IV	1.34E-07	74.7921
FLJ10671	8295	350/400 kDa PCAF-associated factor	1.36E-07	74.68815
ACTRI	90	activin A receptor, type II-like kinase 2	1.41E-07	74.5842
Arc-1	999	cell-CAM 120/80	1.45E-07	74.48025
ARNTL	406	basic-helix-loop-helix-PAS orphan MOP3	1.48E-07	74.3763
EBP1	5036	cell cycle protein p38-2G4 homolog	1.51E-07	74.27235
GRIN2A	2903	NMDA receptor subtype 2A	1.53E-07	74.1684
CALN	5530	protein phosphatase 3, catalytic subunit, alpha isoform	1.56E-07	73.9605
CALNA3	5533	protein phosphatase 3 (formerly 2B), catalytic subunit	1.56E-07	73.9605
CALNA2	5532	calcineurin A beta	1.56E-07	73.9605
MGC13823	4838	nodal, mouse, homolog	1.56E-07	73.7526
HDM2, HDI	4193	p53-binding protein MDM2	1.58E-07	73.64865
CAS3	10278	Cas scaffolding protein family member 3	1.63E-07	73.49272
IMD2	7454	Wiskott-Aldrich syndrome protein	1.63E-07	73.49272
HDLCQ11	4023	lipoprotein lipase	1.77E-07	73.3368
MGC10283	5566	protein kinase A catalytic subunit	1.77E-07	73.1289
KAPG	5568	PKA C-gamma	1.77E-07	73.1289
DKFZp781I	5567	PKA C-beta	1.77E-07	73.1289
PMEPA1	56937	transmembrane prostate androgen-induced protein	1.91E-07	72.921
CD41	3674	integrin alpha 2b	1.97E-07	72.81705
CSK	1445	c-src tyrosine kinase	1.99E-07	72.7131
CD51	3685	integrin alpha-V	2E-07	72.60915
FLJ00380	4779	transcription factor 11 (basic leucine zipper type)	2.06E-07	72.5052
CRSP1	5469	vitamin D receptor-interacting protein complex component	2.06E-07	72.40125
BCL10	8915	CARD-like apoptotic protein	2.06E-07	72.2973
ESG	7088	enhancer of split groucho 1	2.13E-07	72.19335
ESA1	10524	cPLA2 interacting protein	2.26E-07	72.0894
FSHR	2492	follicle stimulating hormone receptor	2.28E-07	71.98545
MGC11098	6622	non A4 component of amyloid	2.29E-07	71.8815
PUR-ALPHA	5813	purine-rich single-stranded DNA-binding protein alpha	2.45E-07	71.77755
FLJ10538	10765	retinoblastoma-binding protein 2, homolog 1A	2.46E-07	71.6736
CIG	2335	fibronectin 1	2.46E-07	71.56965
HU-2	6196	ribosomal protein S6 kinase alpha 2	2.6E-07	71.4657
ADAM15	8751	metargidin	2.69E-07	71.36175
FHL3	2275	LIM-only protein FHL3	2.71E-07	71.20582
HU-1	6195	dJ590P13.1 (ribosomal protein S6 kinase, 90kD)	2.71E-07	71.20582
ZNF189	7743	zinc finger protein 189	2.71E-07	71.0499
DKFZp781F	5325	PLAG-like 1	2.81E-07	70.94595

GUD	7490	Wilms tumor 1	3.04E-07	70.842
ANX2	302	lipocortin II	3.08E-07	70.73805
AATF	26574	apoptosis antagonizing transcription factor	3.13E-07	70.6341
CP107	5933	retinoblastoma-like 1 (p107)	3.13E-07	70.53015
MGF	6776	signal transducer and activator of transcription 5A	3.17E-07	70.4262
CDHF12	5979	ret proto-oncogene (multiple endocrine neoplasia)	3.48E-07	70.32225
CDK6	1021	cell division protein kinase 6	3.49E-07	70.2183
COL3A1	1281	collagen, fetal	3.58E-07	70.11435
CD222	3482	cation-independent mannose-6 phosphate receptor	3.67E-07	70.0104
GFRP1	3164	hormone receptor	3.77E-07	69.90644
CD140A	5156	platelet-derived growth factor receptor, alpha polypeptide	3.82E-07	69.75052
CD140B	5159	platelet-derived growth factor receptor beta	3.82E-07	69.75052
MAD2	4601	Max-related transcription factor	3.87E-07	69.59459
GCN5	2648	GCN5 general control of amino-acid synthesis 5-like 2	4.28E-07	69.49064
POMP100	6421	polypyrimidine tract-binding protein-associated splicing	4.35E-07	69.38669
CADTK	2185	proline-rich tyrosine kinase 2	4.43E-07	69.28274
CD90	7070	Thy-1 membrane glycoprotein precursor variant 2	4.47E-07	69.17879
GTF2D	6908	TATA box binding protein	4.5E-07	69.07484
KIAA1047	9611	nuclear receptor co-repressor 1	4.57E-07	68.97089
DRIP5	54386	telomeric repeat binding factor 2, interacting protein	4.66E-07	68.86694
CD221	3480	insulin-like growth factor 1 receptor	4.86E-07	68.76299
CD209	30835	C-type lectin domain family 4, member L	5.03E-07	68.65904
BRN1	5455	POU domain class 3, transcription factor 3	5.05E-07	68.55509
SP1	6667	Sp1 transcription factor	5.09E-07	68.45114
ANF	8820	homeobox, ES cell expressed 1	5.22E-07	68.34719
CBP35	3958	galectin-3 internal	5.51E-07	68.24324
ERK	5594	protein tyrosine kinase ERK2	5.91E-07	68.13929
AHC	190	nuclear receptor subfamily 0, group B, member 1	6.24E-07	68.03534
CCL3	6348	small inducible cytokine A3 (homologous to mouse Mip-1a)	6.41E-07	67.87942
CC-CKR-5	1234	chemokine receptor CCR5	6.41E-07	67.87942
D11S636	7536	splicing factor 1	6.46E-07	67.72349
CORD2	1406	cone-rod homeobox	6.55E-07	67.61954
DKFZp434K	25942	transcriptional regulator, SIN3A	6.64E-07	67.51559
MGC41878	5579	protein kinase C, beta 1	6.75E-07	67.41164
FLJ40509	3551	inhibitor of kappa light polypeptide gene enhancer in B-cell	6.98E-07	67.30769
HSF1	3297	heat shock transcription factor 1	7.42E-07	67.20374
CD217	23765	interleukin 17 receptor A	7.47E-07	67.04782
FLJ95963	84818	interleukin 17 receptor-like	7.47E-07	67.04782
FLJ42306	5494	protein phosphatase 1A	7.56E-07	66.89189
CARD4	10392	caspase recruitment domain family, member 4	7.76E-07	66.78794
DAPK	1612	death-associated protein kinase 1	8.16E-07	66.63202
DKFZp686E	4131	microtubule-associated protein 1B	8.16E-07	66.63202
DKFZp686C	2775	guanine nucleotide binding protein (G protein)	8.27E-07	66.21622
GNAZ	2781	guanine nucleotide binding protein (G protein)	8.27E-07	66.21622
87U6	2773	guanine nucleotide binding protein (G protein)	8.27E-07	66.21622
GNAI1	2770	Gi1 protein alpha subunit	8.27E-07	66.21622
GIP	2771	guanine nucleotide binding protein (G protein)	8.27E-07	66.21622

AHO	2778	guanine nucleotide binding protein (G protein)	8.27E-07	66.21622
SGK	6446	serum/glucocorticoid regulated kinase 1	8.29E-07	65.85239
CCNB2	9133	cyclin B2	8.35E-07	65.69647
CDC25	995	m-phase inducer phosphatase 3	8.35E-07	65.69647
GNB2L1	10399	proliferation-inducing gene 21	8.54E-07	65.54054
CD124	3566	interleukin-4 receptor alpha chain	8.54E-07	65.38462
CD132	3561	common cytokine receptor gamma chain	8.54E-07	65.38462
1-May	5580	protein kinase C, delta	8.58E-07	65.22869
BAPX2	4824	NK3 homeobox 1	8.95E-07	65.12474
B23	4869	nucleophosmin 1	9.36E-07	65.02079
CCNB	891	G2/mitotic-specific cyclin B1	9.43E-07	64.91684
CD66D	1084	nonspecific cross-reacting antigen	9.94E-07	64.81289
HEP-NOS	4843	nitric oxide synthase, macrophage	1.06E-06	64.70894
GIG8	3398	cell growth-inhibiting gene 8	1.08E-06	64.60499
MAP2K1	5604	protein kinase, mitogen-activated, kinase 1	1.08E-06	64.50104
AUTS9	4233	scatter factor receptor	1.09E-06	64.39709
FYN	2534	src-like kinase	1.15E-06	64.29314
BPTP3	5781	protein tyrosine phosphatase, non-receptor type 11	1.15E-06	64.18919
MGC2237	5465	peroxisome proliferator-activated receptor alpha	1.29E-06	64.08524
CLN10	1509	cathepsin D	1.3E-06	63.98129
FLJ16691	5829	paxillin	1.32E-06	63.87734
HCK	3055	hemopoietic cell kinase	1.35E-06	63.77339
ACTR	8202	CBP-interacting protein	1.36E-06	63.66944
CM-AVM	5921	GTPase activating protein	1.38E-06	63.56549
CDK8	1024	CDK8 protein kinase	1.45E-06	63.46154
MGC12610	4314	matrix metalloproteinase 3	1.46E-06	63.35759
BCL2L3	4170	induced myeloid leukemia cell differentiation protein	1.48E-06	63.25364
CTF	4782	CCAAT-binding transcription factor	1.51E-06	63.14969
CEP52	7311	ubiquitin-52 amino acid fusion protein	1.51E-06	62.99376
FLJ25987	7314	polyubiquitin B	1.51E-06	62.99376
CAMK4	814	CAM kinase IV	1.54E-06	62.83784
AGMX1	695	dominant-negative kinase-deficient Brutons tyrosine	1.64E-06	62.73389
HSPG	6383	heparan sulfate proteoglycan core protein	1.68E-06	62.62994
CDC14A	8556	CDC14 homolog A	1.69E-06	62.52599
MAD7	6945	transcription factor-like 4	1.93E-06	62.42204
I-REL	5971	v-rel avian reticuloendotheliosis viral oncogene homolog B	1.94E-06	62.31809
BMP7	655	osteogenic protein 1	2.03E-06	62.21414
BMP2	650	bone morphogenetic protein 2	2.07E-06	62.05821
BMP6	654	bone morphogenetic protein 6	2.07E-06	62.05821
CRTC1	23373	transducer of regulated cAMP response element-binding	2.11E-06	61.90229
NR0B2	8431	short heterodimer partner	2.21E-06	61.79834
CCS-3	1915	translation elongation factor 1 alpha 1-like 14	2.22E-06	61.69439
CDK4	1019	cyclin-dependent kinase 4	2.35E-06	61.59044
EMC19	6500	transcription elongation factor B (SIII), polypeptide 1-like	2.4E-06	61.48649
NRIP1	8204	receptor interacting protein 140	2.44E-06	61.38254
MGC12651	5588	protein kinase C, theta	2.45E-06	61.27859
AP-1	3726	activator protein 1	2.48E-06	61.17464

CAML1	3897	neural cell adhesion molecule L1	2.58E-06	61.07069
ACT2	6351	lymphocyte-activation gene 1	2.68E-06	60.96674
ACP1	52	red cell acid phosphatase 1	2.79E-06	60.86279
C-2k	1025	cell division protein kinase 9	2.8E-06	60.75884
AMPH2	274	bridging integrator 1	2.88E-06	60.65489
FLJ22252	64321	SRY (sex determining region Y)-box 17	2.89E-06	60.55094
FLJ25655	5885	nuclear matrix protein 1	2.93E-06	60.44699
FLT4	2324	fms-related tyrosine kinase 4	2.93E-06	60.34304
SIR2L1	23411	silent mating type information regulation 2 homolog	3.03E-06	60.23909
DKFZp686C	8848	transforming growth factor beta-stimulated protein TSC-22	3.23E-06	60.13514
AAG6	5578	aging-associated gene 6	3.28E-06	60.03119
RES4-26	6047	small nuclear RING finger protein	3.54E-06	59.92723
KPD	5078	paired box gene 4	3.55E-06	59.82328
BMP5	653	bone morphogenetic protein 5	3.61E-06	59.71933
APBA1	320	amyloid beta A4 precursor protein-binding, family A	3.91E-06	59.61538
CLG4B	4318	gelatinase B	3.96E-06	59.51143
CCR1	1230	chemokine (C-C motif) receptor 1	4.09E-06	59.40748
PP2Ac	5515	protein phosphatase 2, catalytic subunit, alpha isoform	4.1E-06	59.30353
CUL3	8452	cullin 3	4.16E-06	59.19958
ALK1	6590	secretory leukocyte protease inhibitor	4.16E-06	59.09563
IPOA1	3838	karyopherin alpha 2	4.17E-06	58.99168
BUB1B	701	budding uninhibited by benzimidazoles 1 (yeast homolog)	4.27E-06	58.88773
PI4K-BETA	5298	type III phosphatidylinositol 4-kinase beta	4.29E-06	58.78378
MGC21659	7069	spot 14 protein	4.33E-06	58.67983
IMB1	3837	importin beta-1 subunit	4.55E-06	58.57588
AHR	196	aromatic hydrocarbon receptor	4.57E-06	58.47193
DKFZp686E	6772	signal transducer and activator of transcription-1	4.58E-06	58.36798
KIAA0339	9739	SET domain containing 1A	4.61E-06	58.26403
GRB-IR	2887	growth factor receptor-bound protein 10	4.64E-06	58.16008
CAM2	816	proline rich calmodulin-dependent protein kinase	4.77E-06	57.90021
CAMK2A	815	CaM kinase II alpha subunit	4.77E-06	57.90021
CAMK	818	calcium/calmodulin-dependent protein kinase	4.77E-06	57.90021
CAMK2D	817	calcium/calmodulin-dependent protein kinase II delta	4.77E-06	57.90021
CRM1	7514	exportin 1 (CRM1, yeast, homolog)	4.86E-06	57.64033
FLJ14651	10401	protein inhibitor of activated STAT, 3	5.1E-06	57.53638
CIN85	30011	SH3-domain kinase binding protein 1	5.19E-06	57.43243
CASP7	840	caspase 7, apoptosis-related cysteine peptidase	5.21E-06	57.32848
AAD10	8085	trinucleotide repeat containing 21	5.41E-06	57.22453
FLJ36605	7431	vimentin	5.45E-06	57.12058
RRAS	6237	Oncogene RRAS	5.65E-06	57.01663
ARA54	9604	androgen receptor associated protein 54	5.69E-06	56.86071
1200006M	55827	androgen receptor complex-associated protein	5.69E-06	56.86071
EPK2	23683	protein kinase C, nu	5.91E-06	56.70478
BACH1	83990	BRCA1-interacting protein 1	6.02E-06	56.54886
MAZR	23598	zinc finger protein 278	6.02E-06	56.54886
MGC26418	5173	rimorphin	6.04E-06	56.39293
GH	2688	pituitary growth hormone	6.13E-06	56.28898

APLP	333	amyloid-like protein 1	6.18E-06	56.18503
CASP3	836	caspase 3, apoptosis-related cysteine protease	6.34E-06	56.08108
ARNO	9266	cytohesin 2	6.47E-06	55.97713
JAK2	3717	janus kinase 2	6.53E-06	55.87318
CNR2	56142	KIAA0345-like 8	6.62E-06	55.76923
DKFZp586N	4088	mad homolog JV15-2	6.67E-06	55.66528
ELA2	1991	polymorphonuclear elastase	6.74E-06	55.56133
CNR1	56144	ortholog of mouse CNR1	6.74E-06	55.45738
DELTA	7528	YY1 transcription factor	6.82E-06	55.35343
STAT4	6775	signal transducer and activator of transcription 4	6.87E-06	55.24948
IKBKE	9641	inhibitor of kappa light polypeptide gene enhancer in B-cell	7.17E-06	55.14553
DTR	1839	heparin-binding epidermal growth factor	7.4E-06	55.04158
TREB5	7494	box binding protein 1	7.73E-06	54.93763
CSEN	30818	calsenilin, presenilin-binding protein, EF hand transcription factor	7.89E-06	54.83368
F-SRC-1	8648	nuclear receptor coactivator 1	8.04E-06	54.72973
GATA3	2625	GATA binding protein 3	8.05E-06	54.62578
MGC13000	5058	p21/Cdc42/Rac1-activated kinase 1 (STE20 homolog, yeast)	8.12E-06	54.52183
A1A	5265	serine proteinase inhibitor, clade A, member 1	8.22E-06	54.41788
NR1B1	5914	nucleophosmin-retinoic acid receptor alpha fusion protein	8.4E-06	54.31393
EBP50	9368	solute carrier family 9 (sodium/hydrogen exchanger)	8.47E-06	54.20998
MYF3	4654	myogenic differentiation 1	8.8E-06	54.05405
OPMD	8106	poly(A) binding protein, nuclear 1	8.8E-06	54.05405
ALIEN	9318	thyroid receptor interacting protein 15	9.14E-06	53.89813
CF2R	2149	thrombin receptor	9.23E-06	53.79418
PLCG2	5336	phospholipase C gamma 2	9.32E-06	53.63825
APKD2	5311	polycystic kidney disease 2 (autosomal dominant)	9.32E-06	53.63825
ARF1	375	ADP-ribosylation factor 1	9.59E-06	53.48233
MGC3672	5500	protein phosphatase 1, catalytic subunit, delta isoform	9.86E-06	53.37838
ABL	25	proto-oncogene tyrosine-protein kinase ABL1	1.01E-05	53.27443
G13	10672	guanine nucleotide binding protein (G protein), alpha 13	1.01E-05	53.1185
GNA12	2768	guanine nucleotide binding protein (G protein) alpha 12	1.01E-05	53.1185
CDK5	1020	protein kinase CDK5 splicing	1.02E-05	52.96258
CRKL	1399	v-crk avian sarcoma virus CT10 oncogene homolog-like	1.03E-05	52.85863
CLD1	9076	claudin 1	1.04E-05	52.75468
FLJ44809	2475	FKBP12-rapamycin complex-associated protein 1	1.07E-05	52.65073
CACNB3	784	calcium channel, voltage-dependent, beta 3 subunit	1.08E-05	52.54678
GABATHG	6529	solute carrier family 6 (neurotransmitter transporter, GABA)	1.08E-05	52.33888
HPC-1	6804	syntaxin 1A (brain)	1.08E-05	52.33888
FLJ12615	64398	MAGUK p55 subfamily member 5	1.08E-05	52.33888
ACTA	58	alpha skeletal muscle actin	1.09E-05	51.97505
ACT	72	alpha-actin 3	1.09E-05	51.97505
ACTC	70	cardiac muscle alpha actin 1	1.09E-05	51.97505
AAT6	59	actin, alpha 2, smooth muscle, aorta	1.09E-05	51.97505
V75	7448	complement S-protein	1.1E-05	51.71518
ARNO3	9265	general receptor of phosphoinositides 1	1.14E-05	51.61123
DKFZp667C	10482	nuclear RNA export factor 1	1.16E-05	51.50728
APPBP2	10513	amyloid beta precursor protein-binding protein 2	1.16E-05	51.40333

ABI-1	10006	nap1 binding protein	1.18E-05	51.29938
BMK1	5598	mitogen-activated protein kinase 7	1.18E-05	51.19543
BMP2B	652	bone morphogenetic protein 4	1.2E-05	51.09148
CARD3	8767	CARD-containing interleukin-1 beta-converting enzyme	1.21E-05	50.98753
IRF4	3662	interferon regulatory factor 4	1.23E-05	50.88358
ARMD3	10516	fibulin 5	1.25E-05	50.77963
MGC43321	5896	recombination activating protein 1	1.28E-05	50.6237
RAG-2	5897	recombination activating gene 2	1.28E-05	50.6237
BCCIP	56647	cdk inhibitor p21 binding protein	1.28E-05	50.46778
AAA	351	amyloid beta A4 protein	1.28E-05	50.36383
BCGF-1	3565	B cell growth factor 1	1.3E-05	50.25988
BING2	1616	death-associated protein 6	1.33E-05	50.15593
FADK	5747	focal adhesion kinase 1	1.33E-05	50.05198
PC	5624	protein C (inactivator of coagulation factors Va and VIIIa)	1.37E-05	49.94802
PAX2	5076	paired box protein 2	1.38E-05	49.84407
FBN	2200	fibrillin 1	1.39E-05	49.74012
G1P2	9636	ubiquitin cross-reactive protein	1.4E-05	49.63617
FLJ94509	2672	zinc finger protein 163	1.43E-05	49.53222
BDB	4920	neurotrophic tyrosine kinase receptor-related 2	1.5E-05	49.42827
FLJ17670	7535	zeta-chain associated protein kinase 70kDa	1.55E-05	49.32432
CEBP	64506	cytoplasmic polyadenylation element binding protein 1	1.58E-05	49.22037
C-FMS	1436	macrophage colony stimulating factor I receptor	1.59E-05	49.11642
FLJ26658	3693	integrin, beta 5	1.61E-05	49.01247
CNTFR	1271	CNTFR alpha	1.67E-05	48.85655
CD118	3977	CD118 antigen	1.67E-05	48.85655
CBX	10951	heterochromatin protein p25 beta	1.7E-05	48.70062
MAF	4094	Avian musculoaponeurotic fibrosarcoma (MAF)	1.71E-05	48.59667
GRIP1	10499	glucocorticoid receptor-interacting protein-1	1.71E-05	48.49272
KLK-L6	43847	kallikrein-like protein 6	1.71E-05	48.38877
C358B7.1	7329	ubiquitin-conjugating enzyme E2I	1.72E-05	48.28482
ARP1	7026	transcription factor COUP 2	1.73E-05	48.18087
KIAA0403	4988	mu opiate receptor	1.74E-05	48.07692
MGC11154	5879	migration-inducing gene 5	1.74E-05	47.97297
MGC87674	10011	steroid receptor RNA activator 1 (complexes with NCOA1)	1.77E-05	47.86902
DKFZp686N	2099	estrogen receptor 1	1.78E-05	47.76507
C1orf12	54583	egl nine homolog 1	1.8E-05	47.66112
HsT17432	4091	MAD, mothers against decapentaplegic homolog 6	1.85E-05	47.55717
ARF6	382	ADP-ribosylation factor 6	1.85E-05	47.40125
ARFGEP10(	9922	IQ motif and Sec7 domain 1	1.85E-05	47.40125
BM600	3909	epiligrin alpha 3 subunit	1.91E-05	47.24532
C-BAS/HAS	3265	c-ras-Ki-2 activated oncogene	1.93E-05	47.14137
DKFZp781F	80314	enhancer of polycomb 1	1.93E-05	47.03742
BF1	2290	oncogene QIN	1.94E-05	46.93347
ACT	71	actin-like protein	2.27E-05	46.77755
ACTB	60	PS1TP5-binding protein 1	2.27E-05	46.77755
ASC	29108	apoptosis-associated speck-like protein containing a CARD	2.29E-05	46.62162
APCA	773	brain calcium channel 1	2.34E-05	46.51767

AF-6	4301	myeloid/lymphoid or mixed-lineage leukemia	2.35E-05	46.41372
KIAA1303	57521	regulatory associated protein of mTOR	2.35E-05	46.30977
ATP1A1	476	Na+/K+ ATPase 1	2.36E-05	46.20582
ACVRL1	94	activin A receptor, type II-like kinase 1	2.38E-05	46.10187
DFNA1	1729	diaphanous 1	2.46E-05	45.99792
CDX-3	1045	caudal type homeo box transcription factor 2	2.56E-05	45.89397
MPO	4353	myeloperoxidase	2.66E-05	45.73805
ALB	213	cell growth inhibiting protein 42	2.66E-05	45.73805
ADAM12	8038	a disintegrin and metalloproteinase domain 12	2.68E-05	45.58212
C2orf5	150684	MURR1	2.7E-05	45.47817
HRSP12	10247	heat-responsive protein 12	2.8E-05	45.37422
EHK3	2045	Eph homology kinase-3	2.81E-05	45.27027
COL1A2	1278	collagen I, alpha-2 polypeptide	2.83E-05	45.11435
COL1A1	1277	alpha 1 type I collagen	2.83E-05	45.11435
ALK	238	anaplastic lymphoma kinase Ki-1	2.89E-05	44.95842
CYT4	27128	cytohesin-4	2.94E-05	44.85447
FLJ11460	6844	synaptobrevin 2	2.96E-05	44.69854
FLJ23079	6616	synaptosomal-associated protein 25	2.96E-05	44.69854
ARVD	7043	transforming growth factor, beta 3	3.06E-05	44.54262
MGC11689	7042	transforming growth factor, beta 2	3.11E-05	44.43867
VASP	7408	vasodilator-stimulated phosphoprotein	3.17E-05	44.33472
CD309	3791	vascular endothelial growth factor receptor 2	3.2E-05	44.23077
CACH2	775	voltage-gated calcium channel alpha subunit Cav1.2	3.23E-05	44.02287
CAB1	782	dihydropyridine-sensitive L-type, calcium channel beta-1	3.23E-05	44.02287
BKTM	3778	bA205K10.1	3.23E-05	44.02287
AD3	5663	presenilin 1	3.25E-05	43.81497
HMOX2	3163	heme oxygenase (decycling) 2	3.28E-05	43.65904
CACNB2	783	calcium channel, voltage-dependent, beta 2 subunit	3.28E-05	43.65904
FLT	2321	vascular endothelial growth factor/vascular permeability	3.39E-05	43.50312
PTP1B	5770	protein tyrosine phosphatase 1B	3.48E-05	43.39917
F11	2160	platelet coagulation factor XI	3.76E-05	43.24324
APOH	350	beta-2-glycoprotein I	3.76E-05	43.24324
CRC18	1630	deleted in colorectal cancer protein	3.8E-05	43.08732
BABL	8091	high-mobility group (nonhistone chromosomal)	3.86E-05	42.98337
ATF3	467	activating transcription factor 3	3.88E-05	42.87942
C20orf36	4661	myelin transcription factor 1	4.01E-05	42.77547
CALNB1	5534	protein phosphatase 3, regulatory subunit B, alpha isoform 1	4.05E-05	42.61954
PPP3R2	5535	protein phosphatase 3, regulatory subunit B (calcineurin B)	4.05E-05	42.61954
ADCAD2	4040	low density lipoprotein receptor-related protein 6	4.05E-05	42.46362
HHO.C1	3217	homeo box B7	4.05E-05	42.30769
GATA6	2627	GATA binding protein 6	4.05E-05	42.30769
FLJ11330	29110	TANK-binding kinase 1	4.18E-05	42.15177
FLJ43335	5792	leukocyte antigen-related (LAR) PTP receptor	4.29E-05	42.04782
CD142	2152	coagulation factor III (thromboplastin, tissue factor)	4.3E-05	41.94387
ALK-6	658	bone morphogenetic protein receptor, type IB	4.35E-05	41.83992
ARHGAP1	392	Rho GTPase activating protein 1	4.45E-05	41.73597
ARB1	408	arrestin beta 1	4.46E-05	41.63202

ALPS2	843	caspase 10	4.57E-05	41.52807
FLJ97193	10023	proto-oncogene FRAT1	4.59E-05	41.42412
AIS	8626	tumor protein p63	4.61E-05	41.32017
GEFT	115557	RAC/CDC42 exchange factor	4.71E-05	41.21622
DAP-150	1639	p150-glued	4.75E-05	41.11227
CLG1	4317	PMNL collagenase	4.8E-05	41.00832
FLJ12099	5602	mitogen-activated protein kinase 10	5.05E-05	40.90437
DKFZp686A	6789	dJ211D12.2 (serine/threonine kinase 4 (MST1, KRS2))	5.07E-05	40.80042
NR1I1	7421	vitamin D (1,25-dihydroxyvitamin D3) receptor	5.08E-05	40.69647
CD119	3459	AVP, type 2	5.1E-05	40.59252
NFE2L2	4780	nuclear factor (erythroid-derived 2)-like 2	5.26E-05	40.48857
G-ALPHA-h	7052	TGase-H	5.3E-05	40.38462
GCCR	2908	glucocorticoid receptor	5.48E-05	40.28067
ARHGAP14	9901	WAVE-associated Rac GTPase activating protein	5.62E-05	40.17672
PTP-PEST	5782	protein-tyrosine phosphatase G1	5.69E-05	40.07277
MGC10556	7205	zyxin related protein 1	5.72E-05	39.96881
ABT1	29777	activator of basal transcription 1	5.84E-05	39.86486
AF17q25	10801	MLL septin-like fusion	5.84E-05	39.76091
FLJ31482	8936	verprolin homology domain-containing protein 1	6.04E-05	39.65696
ALL	613	breakpoint cluster region	6.05E-05	39.55301
PFN1	5216	profilin 1	6.13E-05	39.44906
ATOD4	9021	suppressor of cytokine signaling 3	6.28E-05	39.34511
CDC25	5923	Ras-specific nucleotide exchange factor CDC25	6.36E-05	39.24116
GLUH1	2890	glutamate receptor, ionotropic, AMPA 1	6.47E-05	39.08524
DKFZp781H	5924	Ras protein-specific guanine nucleotide-releasing factor 2	6.47E-05	39.08524
RPS7	6201	ribosomal protein S7	6.5E-05	38.92931
DKFZp781D	6857	synaptotagmin I	6.75E-05	38.82536
BEK	2263	FGF receptor	6.87E-05	38.72141
IRF5	3663	interferon regulatory factor 5	7.11E-05	38.61746
60B8AG	6280	calgranulin B	7.43E-05	38.51351
HRMT1L3	10196	protein arginine methyltransferase 3	7.57E-05	38.40956
C20orf97	57761	p65-interacting inhibitor of NF-kappaB	7.6E-05	38.30561
RGS14	10636	regulator of G-protein signalling 14	7.6E-05	38.20166
C5FW	28951	tribbles homolog 2 (Drosophila)	7.64E-05	38.09771
EAR1	9572	thyroid hormone receptor, alpha-like	7.73E-05	37.99376
NCF1	653361	NADPH oxidase organizer 2	7.79E-05	37.88981
BAD	572	BCL2-binding protein	7.88E-05	37.78586
PRL	5617	prolactin	8.07E-05	37.68191
DNMT3A	1788	DNA (cytosine-5-)-methyltransferase 3 alpha	8.14E-05	37.57796
PRLR	5618	prolactin receptor	8.29E-05	37.47401
KRML	9935	transcription factor MAFB	8.37E-05	37.37006
DOCK1	1793	dedicator of cyto-kinesis 1	8.38E-05	37.26611
FLJ26625	4067	v-yes-1 Yamaguchi sarcoma viral related oncogene	8.48E-05	37.16216
KIAA1415	57580	phosphatidylinositol-3,4,5-trisphosphate-dependent Rac	8.67E-05	37.00624
DOCK2	1794	dedicator of cytokinesis 2	8.67E-05	37.00624
AFBP	3484	amniotic fluid binding protein	8.7E-05	36.85031
EAP1	9232	ESP1-associated protein 1	8.86E-05	36.74636

FACT	6749	chromatin-specific transcription elongation factor 80 kDa	9.02E-05	36.64241
BIP	3309	Heat-shock 70kD protein-5 (glucose-regulated protein)	9.04E-05	36.53846
DKFZp586I(	84231	TNF receptor-associated factor 7	9.07E-05	36.43451
MMP-7	4316	uterine matrilysin	9.1E-05	36.33056
ATR	545	MEC1, mitosis entry checkpoint 1, homolog	9.13E-05	36.22661
KOR	4986	opioid receptor, kappa 1	9.19E-05	36.12266
FCA/MR	83953	immunity related factor	9.27E-05	36.01871
FLJ43224	4738	neural precursor cell expressed, developmentally	9.54E-05	35.86279
BS4	51667	NY-REN-18 antigen	9.54E-05	35.86279
MGC13185	7334	ubiquitin carrier protein N	9.59E-05	35.70686
GRB1	5295	phosphoinositide-3-kinase, regulatory subunit, polypeptide	9.63E-05	35.60291
ADHR	554	arginine vasopressin receptor 2	9.69E-05	35.49896
BMP14	8200	cartilage-derived morphogenetic protein-1	9.71E-05	35.39501
SYN1	6853	brain protein 4.1	0.000102	35.29106
MGC70609	7422	vascular permeability factor	0.000103	35.18711
IBMPFD	7415	valosin-containing protein	0.000105	35.08316
GF1	6654	son of sevenless homolog 1	0.000106	34.97921
FLJ25596	6655	son of sevenless homolog 2	0.000108	34.87526
HPE2	6496	holoprosencephaly 2, alobar or semilobar	0.00011	34.77131
HUMSIAH	6477	seven in absentia homolog 1	0.000111	34.66736
KIAA1625	57154	Smad-specific E3 ubiquitin ligase 1	0.000115	34.56341
MAPKAPK2	9261	mitogen-activated protein kinase-activated protein kinase	0.000116	34.45946
AIK	6790	serine/threonine protein kinase 6	0.000118	34.35551
EFNB2	1948	ephrin B2	0.000121	34.25156
MGC15060	5586	protein kinase N2	0.000124	34.14761
ARB2	409	arrestin, beta 2	0.000126	34.04366
DKFZp686I(	5327	alteplase	0.000133	33.93971
PRKAR2	5577	protein kinase, cAMP-dependent, regulatory, type II, alpha	0.000134	33.83576
CED	7040	TGF-beta 1 protein	0.000136	33.73181
OT-R	5021	oxytocin receptor	0.000137	33.62786
JAK-3	3718	Janus kinase 3	0.000138	33.52391
CD49f	3655	integrin alpha6B	0.000144	33.41996
CLG3	4322	matrix metalloproteinase 13	0.000144	33.31601
CLR11.4	171389	NLR family, pyrin domain containing 6	0.000148	33.21206
HNRPE2	5094	alpha-CP2	0.000149	33.10811
CLG	4312	interstitial collagenase	0.000152	33.00416
ABRI	9445	BRICHOS domain containing 2B	0.000153	32.90021
EPHA4	2043	tyrosine-protein kinase receptor SEK	0.000155	32.79626
AAT3	7048	transforming growth factor, beta receptor II	0.000161	32.69231
APOA1	335	apolipoprotein A1	0.000166	32.58836
GHDX	6658	SRY (sex determining region Y)-box 3	0.000168	32.48441
ARHG	391	ras homolog gene family, member G (rho G)	0.000169	32.32848
DOCK4	9732	dedicator of cytokinesis 4	0.000169	32.32848
MEF2C	4208	MADS box transcription enhancer factor 2, polypeptide C	0.000172	32.01663
DKFZp686I:	4209	myocyte enhancer factor 2D	0.000172	32.01663
FLJ32599	4207	myocyte enhancer factor 2B	0.000172	32.01663
ADCAD1	4205	myocyte enhancer factor 2A	0.000172	32.01663

BP-53	3486	binding protein 53	0.000173	31.75676
ELF3	1999	E74-like factor 3 (ets domain transcription factor, epithelial)	0.000178	31.65281
BIRC7	79444	livin inhibitor of apoptosis	0.000182	31.54886
SCAP1	8631	src kinase associated phosphoprotein 1	0.000196	31.44491
ADAP	2533	FYN-binding protein (FYB-120/130)	0.000199	31.34096
ACC-4	1512	cathepsin H	0.0002	31.23701
CSNK1E	1454	casein kinase 1 epsilon	0.000202	31.13306
F-TCF	3082	hepatocyte growth factor	0.000203	31.02911
C6	5688	proteasome subunit alpha 4	0.00021	30.92516
EIF2AK1	5610	double stranded RNA activated protein kinase	0.000218	30.82121
DKFZp779(	8976	neural Wiskott-Aldrich syndrome protein	0.000219	30.71726
FER	2241	phosphoprotein NCP94	0.000221	30.61331
NOV	5361	plexin 1	0.000227	30.50936
BARS	1487	C-terminal binding protein 1	0.000228	30.40541
FLOT1	10211	flotillin 1	0.000228	30.30146
D3DR	1814	dopamine receptor D3	0.000231	30.19751
CORD7	22999	regulating synaptic membrane exocytosis 1	0.000231	30.04158
RAB10	10890	ras-related GTP-binding protein RAB10	0.000231	30.04158
CAK	780	neurotrophic tyrosine kinase, receptor, type 4	0.000233	29.88565
CLM	3912	laminin, beta 1	0.000235	29.67775
LAMB2	3915	formerly LAMB2	0.000235	29.67775
LAMA	284217	laminin, alpha 1	0.000235	29.67775
DDIT1	1647	DNA damage-inducible transcript 1	0.000236	29.46985
B2-1	9267	homolog of secretory protein SEC7	0.00024	29.3659
BAP-1	6045	ring finger protein 2	0.000245	29.26195
DKFZp586E	9477	Trf (TATA binding protein-related factor)-proximal homolog (Droso	0.000248	29.158
SFRS3	6428	splicing factor, arginine/serine-rich, 20-kD	0.00025	29.05405
C-MPL	4352	thrombopoietin receptor	0.000264	28.9501
CD120a	7132	tumor necrosis factor receptor type 1	0.000268	28.84615
MAPKAPK5	8550	mitogen-activated protein kinase-activated protein kinase 5	0.000268	28.7422
ERN1	2081	endoplasmic reticulum to nucleus signaling 1	0.000288	28.63825
DKFZp781H	9451	eukaryotic translation initiation factor 2-alpha kinase 3	0.000289	28.5343
Bos1	9570	golgi SNAP receptor complex member 2	0.00029	28.43035
AG2S	185	angiotensin receptor 1B	0.000293	28.3264
B2T	3918	laminin, gamma 2 (nicein (100kD), kalinin (105kD))	0.000299	28.22245
AMY-1	26292	associate of myc-1	0.000306	28.1185
GPRC1A	2911	glutamate receptor, metabotropic 1	0.000309	28.01455
CDCD1	4000	limb girdle muscular dystrophy 1B (autosomal dominant)	0.000312	27.9106
DKFZp686F	3611	integrin-linked kinase	0.00032	27.80665
FLJ34027	10657	KH domain containing, RNA binding	0.000327	27.7027
CCT-alpha	6950	T-complex protein 1, alpha subunit	0.000328	27.59875
KCIP-1	7534	phospholipase A2	0.000333	27.4948
AP3Aase	2272	dinucleosidetriphosphatase	0.000335	27.39085
KIAA0190	9100	ubiquitin carboxyl-terminal hydrolase 10	0.000346	27.2869
OS9	10956	amplified in osteosarcoma	0.00036	27.18295
Hs.2563	6863	tachykinin 1	0.000365	27.079
ANKRD28	23243	phosphatase interactor targeting K protein	0.000369	26.97505

HPK38	9833	pEg3 kinase	0.00038	26.8711
INrf2	9817	kelch-like ECH-associated protein 1	0.000388	26.76715
EDNRA	1909	endothelin receptor type A	0.000396	26.6632
GIF	4282	phenylpyruvate tautomerase	0.000402	26.55925
ELF5	2001	epithelium-specific Ets transcription factor 2	0.000402	26.4553
C2H2-171	10472	zinc finger protein 238	0.000404	26.35135
CD104	3691	GP150	0.00041	26.2474
LSP1	4046	F-actin binding and cytoskeleton associated protein	0.000429	26.14345
ELK4	2005	SRF accessory protein 1	0.00044	26.0395
DIF	7124	TNF, monocyte-derived	0.000442	25.93555
EDF	3624	inhibin, beta A (activin A, activin AB alpha polypeptide)	0.000444	25.72765
BMP-11	10220	growth differentiation factor 11	0.000444	25.72765
ACTRIIB	93	activin A type IIB receptor	0.000444	25.72765
DKFZp779N	5340	plasminogen	0.000452	25.51975
C21	79718	transducin (beta)-like 1X-linked receptor 1	0.000454	25.4158
CD18	3689	complement receptor C3 beta-subunit	0.000459	25.25988
ADB2	3681	leukointegrin alpha D	0.000459	25.25988
MGC10182	6591	slug homolog, zinc finger protein	0.000461	25.10395
HCRTR1	3061	orexin receptor 1	0.000465	25
APPS	1508	cysteine protease	0.000468	24.89605
FLJ21263	8729	golgi-specific brefeldin A resistance factor 1	0.000471	24.7921
CD11C	3687	myeloid membrane antigen, alpha subunit	0.000471	24.68815
CMTX	2705	connexin 32	0.000477	24.5842
ELN	2006	tropoelastin	0.000478	24.48025
C14orf18	57820	human enhancer of invasion 10	0.00048	24.3763
BOS3	6495	SIX homeobox 1	0.000496	24.27235
CLOCK	9575	clock homolog (mouse)	0.000497	24.1684
CCNE2	9134	cyclin E2	0.000506	24.06445
CD138	6382	syndecan proteoglycan 1	0.000515	23.9605
BM600-125	3914	laminin B1k chain	0.000529	23.85655
PAK2	5062	p21-activated kinase 2	0.000529	23.7526
GPRC1E	2915	glutamate receptor, metabotropic 5	0.00053	23.64865
ADH	551	arginine vasopressin-neurophysin II	0.000534	23.5447
2-5-3p	23265	exocyst complex component 7	0.000542	23.44075
AUF1	3184	heterogeneous nuclear ribonucleoprotein D (AU-rich)	0.000562	23.3368
AXIN	8312	axin 1	0.000571	23.18087
AXIL	8313	axin 2	0.000571	23.18087
MGC15877	5499	serine/threonine protein phosphatase PP1-alpha 1	0.000574	22.921
PPP1R7	5510	protein phosphatase-1 regulatory subunit 7 beta1	0.000574	22.921
PPP1CC	5501	protein phosphatase 1, catalytic subunit, gamma isoform	0.000574	22.921
F2RL3	9002	coagulation factor II (thrombin) receptor-like 3	0.000575	22.7131
EPHA3	2042	ephrin receptor EphA3	0.000601	22.60915
BCL3	602	B-cell leukemia/lymphoma 3	0.000612	22.5052
FJHN	6928	transcription factor 2, hepatic	0.000689	22.40125
API4	332	survivin variant 3 alpha	0.000723	22.2973
H731	27250	programmed cell death 4	0.000729	22.19335
AR-JP	5071	parkin	0.000739	22.0894

DKFZp547C	25970	SH2B adaptor protein 1	0.000754	21.98545
CD114	1441	colony stimulating factor 3 receptor	0.000755	21.8815
CAMK1	8536	calcium/calmodulin-dependent protein kinase I	0.000775	21.77755
ARF1GAP	55738	ADP-ribosylation factor GTPase activating protein 1	0.000795	21.6736
CD121A	3554	interleukin 1 receptor alpha, type I	0.000796	21.56965
CSA	1442	chorionic somatomammotropin A	0.000805	21.41372
CS-2	1443	placental lactogen	0.000805	21.41372
DBC-1	57805	deleted in breast cancer 1	0.000806	21.2578
APBB1	322	amyloid beta (A4) precursor protein-binding, family B	0.00081	21.15385
GH-V	2689	placental-specific growth hormone	0.000818	21.0499
CD11B	3684	integrin alpha M	0.000833	20.94595
NEF	6285	S100 calcium binding protein, beta (neural)	0.000846	20.842
CD295	3953	leptin receptor	0.000846	20.73805
BA554C12.	9978	ZYP protein	0.000862	20.47817
ELOB	6923	transcription elongation factor B (SIII), polypeptide 2 (18kD)	0.000862	20.47817
SIII	6921	transcription elongation factor B, polypeptide 1	0.000862	20.47817
CUL2	8453	cullin 2	0.000862	20.47817
CD262	8795	death receptor 5	0.000873	20.2183
ZIC	7545	Zic family member 1 (odd-paired Drosophila homolog)	0.000877	20.11435
RALBP1	10928	ralA-binding protein	0.000879	20.0104
ACAN	176	large aggregating proteoglycan	0.000887	19.90644
BN-1	1235	chemokine (C-C motif) receptor 6	0.000916	19.80249
GHBP	2690	somatotropin receptor	0.000929	19.69854
FABP6	2172	ileal bile acid binding protein	0.000932	19.59459
D2DR	1813	seven transmembrane helix receptor	0.000972	19.49064
RP11-3J10.	6461	Src homology 2 domain containing adaptor protein B	0.001033	19.38669
AFP	174	alpha-1-fetoprotein	0.001038	19.28274
CAR	846	extracellular calcium-sensing receptor	0.001052	19.02287
RAMP1	10267	calcitonin receptor-like receptor activity modifying protein 1	0.001052	19.02287
RAMP3	10268	receptor activity modifying protein 3	0.001052	19.02287
RAMP2	10266	receptor (calcitonin) activity modifying protein 2	0.001052	19.02287
GCKR	11183	kinase homologous to SPS1/STE20	0.00108	18.76299
CD2AP	23607	CD2-associated protein	0.001106	18.65904
CMT1F	4747	neurofilament, light polypeptide 68kDa	0.001122	18.55509
FGG	2266	fibrinogen, gamma polypeptide	0.001128	18.34719
FGB	2244	fibrinogen, beta chain	0.001128	18.34719
FGA	2243	fibrinogen, alpha polypeptide	0.001128	18.34719
YWHA1	7533	14-3-3 eta	0.001136	18.13929
DARPP-32	84152	protein phosphatase 1, regulatory (inhibitor) subunit 1B	0.001159	18.03534
LIS1	5048	platelet-activating factor acetylhydrolase, isoform Ib	0.001165	17.93139
CAP	10580	sorbin and SH3 domain containing 1	0.001176	17.82744
HUMORFA(	8826	IQ motif containing GTPase activating protein 1	0.001193	17.72349
APO2	8797	cytotoxic TRAIL receptor	0.001216	17.61954
HOP	10963	stress-induced-phosphoprotein 1	0.001256	17.51559
GNG2	54331	guanine nucleotide binding protein (G protein), gamma 2	0.001324	17.35967
GNB1	2782	transducin beta chain 1	0.001324	17.35967
IB1	9479	mitogen-activated protein kinase 8 interacting protein 1	0.001325	17.20374

MGC48949	5898	ras related v-ral simian leukemia viral oncogene homolog A	0.001343	17.09979
MGC:8389	9519	TBP-related factor 2	0.001344	16.99584
D3S1319E	5217	profilin 2	0.0014	16.89189
BTEB	687	basic transcription element binding protein 1	0.001416	16.78794
DKFZp586H	5908	RAS-related protein RAP1B	0.001467	16.68399
ABD-B	3205	homeo box A9	0.00154	16.58004
HNRPE1	5093	poly(rC)-binding protein 1	0.001574	16.47609
EDN2	1907	endothelin 2	0.001642	16.32017
EDN3	1908	truncated endothelin 3	0.001642	16.32017
LIMK	3984	LIM domain kinase 1	0.001665	16.16424
CD36	948	scavenger receptor class B, member 3	0.001707	16.06029
AA	7003	atrophia areata, peripapillary chorioretinal degeneration	0.001802	15.95634
FLJ16752	59269	human immunodeficiency virus type I enhancer	0.001824	15.85239
GSC	145258	goosecoid	0.001845	15.74844
CSCD	1634	small leucine-rich protein 1B	0.001848	15.64449
MBP	4155	myelin basic protein	0.001856	15.54054
BWCR	1028	cyclin-dependent kinase inhibitor 1C	0.001862	15.43659
DKFZp434N	51277	Ras-associated protein Rap1	0.001867	15.33264
GIG34	6135	60S ribosomal protein L11	0.001916	15.22869
CASP5	838	caspase 5, apoptosis-related cysteine protease	0.00192	15.12474
DKFZp686K	10625	NS1-binding protein	0.001921	14.96881
AIP	9049	aryl hydrocarbon receptor interacting protein	0.001921	14.96881
DKFZp686L	55929	DNMT1 associated protein 1	0.001937	14.76091
DKFZp313G	9628	regulator of G-protein signaling 6	0.001937	14.76091
CRTC2	200186	transducer of regulated cAMP response element	0.001965	14.60499
CD8B	926	CD8b molecule	0.001966	14.44906
CD3-DELTA	915	CD3D antigen, delta polypeptide	0.001966	14.44906
GDF8	2660	myostatin	0.002047	14.29314
OPRD	4985	opioid receptor, delta 1	0.002097	14.18919
ABCDS	1910	Hirschsprung disease 2	0.00214	14.08524
H1	6013	prorelaxin	0.002228	13.98129
CFL2	1073	cofilin 2	0.002294	13.82536
CFL	1072	cofilin 1 (non-muscle)	0.002294	13.82536
CUL1	8454	cullin 1	0.002296	13.66944
GS1	4644	myosin, heavy polypeptide kinase	0.002306	13.56549
CAPB	2048	elk-related tyrosine kinase	0.002315	13.46154
ATX	5168	autotaxin	0.00232	13.35759
ABR	29	active breakpoint cluster region-related protein	0.002331	13.25364
FLJ97602	4867	nephrocystin-1	0.002355	13.14969
DKFZp762F	55662	hypoxia-inducible factor 1, alpha subunit inhibitor	0.002397	13.04574
GIG4	5872	growth-inhibiting gene 4 protein	0.002418	12.94179
DBDR	1816	D1beta dopamine receptor	0.002444	12.83784
SCAR2	10163	suppressor of cyclic-AMP receptor (WASP-family)	0.002642	12.73389
DKFZp779K	5291	PI3-kinase p110 subunit beta	0.00267	12.62994
CD202B	7010	TEK tyrosine kinase, endothelial	0.002694	12.52599
ATF5	22809	activating transcription factor 5	0.002764	12.42204
IB2	23542	mitogen-activated protein kinase 8 interacting protein 2	0.002915	12.31809

CTS02	1513	cathepsin K	0.002917	12.21414
C2orf31	7855	seven-transmembrane receptor frizzled-5	0.002936	12.11019
ELK	2047	ephrin receptor EphB1	0.002959	12.00624
DKFZp566J	9039	UBA3, ubiquitin-activating enzyme E1 homolog	0.002965	11.85031
A-116A10.:	8883	NEDD8-activating enzyme E1 subunit	0.002965	11.85031
DCR1	23405	dicer 1, ribonuclease type III	0.002969	11.69439
EN-7	5880	small G protein	0.002992	11.59044
Hs.89862	8717	tumor necrosis factor receptor type 1 associated death	0.003022	11.48649
NGR	65078	Nogo-66 receptor	0.003026	11.38254
GS2	5873	RAB27A, member RAS oncogene family	0.003029	11.27859
CDK3	1018	cyclin-dependent kinase 3	0.003183	11.17464
EBI6	7185	TNF receptor-associated factor 1	0.003225	11.01871
FLJ14993	84957	receptor expressed in lymphoid tissues	0.003225	11.01871
HDLCQ12	3990	lipase C	0.003297	10.86279
CCNA1	8900	cyclin A1	0.003328	10.75884
CLEVER-1	23166	common lymphatic endothelial and vascular endothelial	0.003531	10.65489
FLJ11090	55775	tyrosyl-DNA phosphodiesterase 1	0.003711	10.55094
CPLX1	10815	complexin 1	0.003715	10.44699
NEC1	5122	proprotein convertase subtilisin/kexin type 1	0.003751	10.34304
53BP1	7158	tumor protein 53-binding protein, 1	0.00382	10.23909
DKFZp586C	8932	methyl-CpG binding domain protein 2	0.003849	10.13514
ABP1	28988	drebrin-like	0.003873	9.927235
BLK	640	BLK nonreceptor tyrosine kinase	0.003873	9.927235
HPK1	11184	hematopoietic progenitor kinase 1	0.003873	9.927235
CAS	1434	CSE1 chromosome segregation 1-like protein	0.004114	9.719335
MGC12657	8828	neuropilin-2a(22)	0.004477	9.615385
CRL	133396	gp130-like monocyte receptor	0.004797	9.511435
HDNF	4908	neurotrophin 3	0.005046	9.407484
HEPTP	5786	protein tyrosine phosphatase, receptor type, A	0.005127	9.303534
NK1R	6869	NK-1 receptor	0.005314	9.199584
KIAA0595	23082	peroxisome proliferator-activated receptor gamma	0.005342	9.095634
HEB	6938	transcription factor 12	0.005459	8.991684
APOER2	7804	apolipoprotein E receptor 2	0.005484	8.887734
CK1	1452	down-regulated in lung cancer	0.005515	8.783784
DKFZp686C	4703	nebulin	0.005671	8.679834
FLJ12859	23528	ZNP-99 transcription factor	0.005698	8.575884
C7orf16	10842	G-substrate	0.00617	8.471933
GEPH	10243	gephyrin	0.006473	8.367983
CALCOCO1	57658	coiled-coil transcriptional coactivator	0.00655	8.160083
CUL4B	8450	cullin-4B	0.00655	8.160083
DKFZp686C	171024	genethonin 2	0.00655	8.160083
AGS1	51655	dexamethasone-induced ras-related protein 1	0.006791	7.952183
IHPS1	4842	neuronal nitric oxide synthase	0.00687	7.796258
SSTR5	6755	somatostatin receptor subtype 5	0.00687	7.796258
AHF	2157	procoagulant component	0.007174	7.640333
EDG-4	9170	lysophosphatidic acid receptor 2	0.007337	7.536383
BSF3	23529	B-cell stimulating factor 3	0.007454	7.432432

EVI16	6659	SRY (sex determining region Y)-box 4	0.007925	7.328482
DLL4	54567	notch ligand DLL4	0.008003	7.224532
FES	2242	proto-oncogene c-fes variant 4	0.008012	7.120582
ERG1	3757	potassium channel HERG1	0.00831	7.016632
FLJ41337	8500	PTPRF interacting protein alpha 1	0.008365	6.912682
ATSV	547	kinesin, heavy chain, member 1A, homolog of mouse	0.008539	6.808732
ZBTB33	10009	zinc finger and BTB domain containing 33	0.008595	6.704782
HEC	10403	kinetochore associated 2	0.008783	6.600832
FX	7114	thymosin beta 4, X-linked	0.009461	6.444906
MGC26307	9087	thymosin beta-4, Y isoform	0.009461	6.444906
ARD1	8260	N-acetyltransferase ARD1	0.009484	6.288981
CABP2	51475	calcium binding protein 2	0.009537	6.185031
AHH	57491	aryl hydrocarbon receptor regulator	0.010417	6.081081
103AS, 15.:	3805	NK cell receptor	0.010804	5.977131
LIMS1	3987	LIM and senescent cell antigen-like domains 1	0.011092	5.873181
HTF9A	5902	RAN binding protein 1	0.011344	5.769231
ATBF1	463	AT motif-binding factor 1	0.011379	5.665281
B56A	5525	serine/threonine protein phosphatase 2A, 56 kDa regulatory subun	0.011453	5.561331
ADORA2	136	adenosine A2b receptor	0.01208	5.45738
CTSS	1520	cathepsin S	0.012228	5.35343
APG12	9140	Apg12 (autophagy, yeast) homolog	0.01277	5.24948
FLJ14040	84148	MYST histone acetyltransferase 1	0.012952	5.14553
MGC16699	5797	protein tyrosine phosphatase, receptor type, mu polypeptide	0.014228	4.989605
CD113	25945	poliovirus receptor-related 3	0.014228	4.989605
CAIN	8021	nucleoporin 214kDa	0.014459	4.781705
2PP2A	6418	template-activating factor I	0.014459	4.781705
ACVRIP1	9863	membrane associated guanylate kinase, WW and PDZ	0.014568	4.62578
CD158F	57292	killer cell immunoglobulin-like receptor, two domains	0.014578	4.52183
CCL24	6369	eotaxin-2	0.0146	4.417879
ARIX	401	arix homeodomain protein	0.015383	4.313929
AD2	348	apolipoprotein E3	0.015608	4.209979
BP	3240	binding peptide	0.01598	4.106029
NR1F2	6096	nuclear receptor RZR-beta	0.016275	4.002079
FLJ20819	7879	Ras-associated protein RAB7	0.017196	3.898129
MSI1	4440	Musashi (Drosophila) homolog 1	0.018013	3.794179
MGC39961	10762	nucleoporin 50kDa	0.018103	3.690229
FKHL8	2297	forkhead box D1	0.018662	3.586279
DKFZp781F	9788	missing in metastasis	0.019066	3.482328
JDP2	122953	Jun dimerization protein 2	0.019406	3.378378
CRSP150	9282	human homolog of yeast RGR1	0.019751	3.274428
CNTF	1270	ciliary neurotrophic factor	0.020607	3.170478
AIGF	2253	androgen-induced growth factor	0.020754	3.066528
KIAA0965	23012	serine/threonine kinase 38 like	0.021827	2.962578
MGC12360	10605	polyadenylate binding protein-interacting protein 1	0.021922	2.858628
DKFZp686F	5597	extracellular signal-regulated kinase, p97	0.023099	2.754678
CD72	971	CD72 molecule	0.023777	2.650728
BACH1	571	BTB and CNC homology 1, basic leucine zipper transcription	0.024841	2.546778

CRF2-9	58985	interleukin 22 receptor, alpha 1	0.025025	2.338877
DIRS1	53833	interleukin-20 receptor II	0.025025	2.338877
FLJ40993	53832	class II cytokine receptor ZCYTOR7	0.025025	2.338877
DKFZp686F	6876	SM22-alpha	0.025435	2.130977
HHR23B	5887	XP-C repair complementing complex 58 kDa	0.02789	1.715177
LPP	4026	LIM domain containing preferred translocation partner	0.02789	1.715177
PCTP	58488	phosphatidylcholine transfer protein	0.02789	1.715177
KARS	3735	lysyl-tRNA synthetase	0.02789	1.715177
FLJ00280	6256	retinoid X receptor, alpha	0.02789	1.715177
FLJ30414	3094	histidine triad nucleotide binding protein 1	0.02789	1.715177
CHA	10732	transcription factor-like 5 (basic helix-loop-helix)	0.02789	1.715177
CD3E	916	CD3e antigen, epsilon polypeptide (TiT3 complex)	0.029458	1.299376
PLS3	5358	T isoform	0.029943	1.195426
2310020H2	23479	nitrogen fixation cluster-like	0.030779	1.091476
CUL5	8065	Vasopressin-activated calcium-mobilizing receptor-1	0.032255	0.987526
CLCR	11330	elastase IV	0.033248	0.883576
GGA3	23163	Golgi-localized, gamma ear-containing, ARF-binding protein 3	0.033895	0.779626
GLK	8491	germinal center kinase-related protein kinase	0.034452	0.675676
ACT1	10758	TRAF3 interacting protein 2	0.036332	0.571726
DKFZp586I	8498	RAN-binding protein-3	0.039071	0.467775
CC10	7356	Uteroglobin (Clara-cell specific 10-kD protein)	0.040149	0.363825
MGC10268	6494	GTPase-activating protein Spa-1	0.041484	0.259875
PROP1	5626	prophet of Pit1, paired-like homeodomain transcription	0.045909	0.155925
DCOH	5092	Pterin-4a-carbinolamine dehydratase	0.047466	0.051975

Topologically significant genes determined from proteomics data

Symbol	Entrez_ID	Description	p value	percentile
MYC	4609	myc proto-oncogene protein	2.15E-08	99.82669
HIRS-1	3667	insulin receptor substrate 1	1.85E-07	99.74003
BCL2	596	B-cell CLL/lymphoma 2	4.21E-07	99.56672
INSRR	3645	IR-related receptor	4.34E-07	99.39341
MGC26306	9414	tight junction protein 2 (zona occludens 2)	4.38E-07	99.2201
GCCR	2908	glucocorticoid receptor	5.98E-07	99.04679
IRF-1	3659	interferon regulatory factor-1	1.01E-06	98.87348
SREBF1	6720	sterol regulatory element binding transcription factor 1	1.06E-06	98.70017
FLJ12859	23528	ZNP-99 transcription factor	1.1E-06	98.52686
ETV3	2117	ets variant gene 3, ETS family transcriptional repressor	1.12E-06	98.35355
ELK4	2005	SRF accessory protein 1	1.15E-06	98.18024
MGC11154	5879	migration-inducing gene 5	1.31E-06	98.00693
TCF-3	83439	transcription factor 7-like 1 (T-cell specific, HMG-box)	1.47E-06	97.487
FLJ36364	6932	transcription factor 7 (T-cell specific, HMG-box)	1.47E-06	97.487
TCF-4	6934	transcription factor 7-like 2 (T-cell specific, HMG-box)	1.47E-06	97.487
DKFZp686E	6772	signal transducer and activator of transcription-1	1.47E-06	97.487
DKFZp586H	51176	lymphoid enhancer binding factor-1	1.47E-06	97.487
E2F-5	1875	E2F transcription factor 5, p130-binding	1.56E-06	96.96707
HNRPE1	5093	poly(rC)-binding protein 1	1.68E-06	96.79376
FLJ36302	7074	T-cell lymphoma invasion and metastasis 1	1.78E-06	96.62045
AMPH2	274	bridging integrator 1	1.79E-06	96.44714
ARF	1029	multiple tumor suppressor 1	1.82E-06	96.27383
EPHEXIN	25791	ephexin	1.9E-06	96.10052
GSK3B	2932	glycogen synthase kinase 3 beta	1.94E-06	95.84055
DKFZp686D	2931	glycogen synthase kinase 3 alpha	1.94E-06	95.84055
NOR-90	7343	upstream binding transcription factor, RNA polymerase I	1.99E-06	95.58059
DKFZp779K	5291	PI3-kinase p110 subunit beta	2.21E-06	95.40728
GRIN1	2902	glutamate [NMDA] receptor subunit zeta 1	2.29E-06	95.23397
FLJ12099	5602	mitogen-activated protein kinase 10	2.35E-06	95.06066
CAP20	1026	CDK-interaction protein 1	2.36E-06	94.88735
MYD88	4615	myeloid differentiation primary response gene 88	2.46E-06	94.71404
LYT-10	4791	nuclear factor of kappa light chain gene enhancer in B-cells	3.26E-06	94.54073
AIS	367	androgen receptor	3.3E-06	94.36742
DKFZp781F	9788	missing in metastasis	3.31E-06	94.19411
PLZF	7704	zinc finger protein 145 (Kruppel-like)	3.49E-06	94.0208
DELTA	7528	YY1 transcription factor	3.75E-06	93.84749
FBL1	6502	CDK2/cyclin A-associated protein p45	3.84E-06	93.67418
BMK1	5598	mitogen-activated protein kinase 7	4.07E-06	93.50087
PIM	5292	Oncogene PIM1	4.31E-06	93.32756
CBF1	3516	recombining binding protein suppressor of hairless	4.44E-06	93.15425
MYL	5371	promyelocytic leukemia, inducer of	4.6E-06	92.98094
HsT17454	5607	MAP kinase kinase MEK5b	4.72E-06	92.80763
ARP1	5308	solurshin	4.98E-06	92.63432
CHEDG1	1901	sphingosine 1-phosphate receptor EDG1	5E-06	92.46101
DPC4	4089	mothers against decapentaplegic homolog 4	5.15E-06	92.28769

BHLHB2	8553	differentially expressed in chondrocytes 1	5.34E-06	92.11438
ETS2	2114	oncogene ETS-2	6.23E-06	91.94107
ENX-1	2146	enhancer of zeste homolog 2 (Drosophila)	6.28E-06	91.76776
ERYF1	2623	NF-E1 DNA-binding protein	6.31E-06	91.59445
NOTCH1	4851	neurogenic locus notch homolog protein 1	6.62E-06	91.42114
DKFZp686H	3065	histone deacetylase 1	6.65E-06	91.24783
HSF1	3297	heat shock transcription factor 1	6.67E-06	91.07452
ARF6	382	ADP-ribosylation factor 6	6.8E-06	90.90121
CEBP	64506	cytoplasmic polyadenylation element binding protein 1	6.85E-06	90.55459
AAA	351	amyloid beta A4 protein	6.85E-06	90.55459
NGR	65078	Nogo-66 receptor	6.85E-06	90.55459
CCNE	898	cyclin Es	7.18E-06	90.12132
FLJ10671	8295	350/400 kDa PCAF-associated factor	7.18E-06	90.12132
HA6116	9759	histone deacetylase 4	7.47E-06	89.86135
BSP1	4086	MAD, mothers against decapentaplegic homolog 1	7.6E-06	89.68804
CDC25	5923	Ras-specific nucleotide exchange factor CDC25	7.67E-06	89.51473
AHR	196	aromatic hydrocarbon receptor	7.84E-06	89.34142
CLOCK	9575	clock homolog (mouse)	7.91E-06	89.16811
G0S24	7538	zinc finger protein, C3H type, 36 homolog (mouse)	7.98E-06	88.9948
CDC18L	990	CDC18 (cell division cycle 18, S.pombe, homolog)-like	7.98E-06	88.82149
ARR3	407	cone arrestin	8.47E-06	88.64818
GNB1	2782	transducin beta chain 1	8.77E-06	88.2149
GNG11	2791	guanine nucleotide-binding protein G(I)/G(S)/G(O)	8.77E-06	88.2149
KIAA1415	57580	phosphatidylinositol-3,4,5-trisphosphate-dependent Rac	8.77E-06	88.2149
G(gamma)1	51764	guanine nucleotide binding protein (G protein), gamma 13	8.77E-06	88.2149
PP2CB	5516	serine/threonine protein phosphatase 2A, catalytic subunit	8.9E-06	87.69497
PP2Ac	5515	protein phosphatase 2, catalytic subunit, alpha isoform	8.9E-06	87.69497
MGC11121	4831	non-metastatic cells 2, protein (NM23) expressed in	1.02E-05	87.43501
E2F-4	1874	E2F transcription factor 4	1.02E-05	87.2617
FLJ25596	6655	son of sevenless homolog 2	1.06E-05	87.00173
GF1	6654	son of sevenless homolog 1	1.06E-05	87.00173
KIAA1515	57646	ubiquitin specific protease 28	1.07E-05	86.74177
PAK2	5062	p21-activated kinase 2	1.08E-05	86.56846
BAF170	6601	SWI3-like protein	1.09E-05	86.39515
C21	79718	transducin (beta)-like 1X-linked receptor 1	1.1E-05	86.22184
CRD-BP	10642	insulin-like growth factor 2 mRNA binding protein 1	1.12E-05	86.04853
IPOA1	3838	karyopherin alpha 2	1.13E-05	85.87522
Hs.54452	10320	Ikaros (zinc finger protein)	1.14E-05	85.70191
AGO	55294	F-box and WD-40 domain protein 7 (archipelago homolog)	1.17E-05	85.5286
DKFZp686C	4790	nuclear factor kappa-B, subunit 1	1.2E-05	85.35529
ASCL1	429	achaete-scute homolog 1	1.23E-05	85.18198
D6S182	3326	heat shock 90kDa protein 1, beta	1.25E-05	85.00867
BP-8	4904	nuclease sensitive element binding protein 1	1.3E-05	84.83536
ABD-B	3205	homeo box A9	1.31E-05	84.66205
MAX	4149	MAX protein	1.31E-05	84.48873
POMP100	6421	polypyrimidine tract-binding protein-associated splicing	1.33E-05	84.31542
CLGI	7076	fibroblast collagenase inhibitor	1.34E-05	84.14211

FOG	161882	friend of GATA-1	1.37E-05	83.9688
HNRNPU	3192	heterogeneous nuclear ribonucleoprotein U	1.38E-05	83.79549
ESA1	10524	cPLA2 interacting protein	1.44E-05	83.62218
FBP	8880	far upstream element-binding protein	1.47E-05	83.44887
AWD	4830	NDP kinase A	1.54E-05	83.27556
MSP23	5052	peroxiredoxin 1	1.58E-05	83.10225
HD3	8841	histone deacetylase 3	1.65E-05	82.92894
CASP8AP2	9994	human FLASH	1.67E-05	82.75563
EAP1	9232	ESP1-associated protein 1	1.68E-05	82.58232
ELK1	2002	ELK1 protein	1.75E-05	82.40901
ENO1	2023	phosphopyruvate hydratase	1.78E-05	82.2357
B1F	2494	liver receptor homolog-1	1.81E-05	82.06239
GLUH1	2890	glutamate receptor, ionotropic, AMPA 1	1.82E-05	81.80243
DKFZp781H	5924	Ras protein-specific guanine nucleotide-releasing factor 2	1.82E-05	81.80243
FLJ16691	5829	paxillin	1.85E-05	81.54246
C/EBP-beta	1051	transcription factor 5	1.92E-05	81.36915
KPD	5078	paired box gene 4	1.97E-05	81.19584
ACVRLK3	657	bone morphogenetic protein receptor, type IA	2.01E-05	81.02253
NR1I1	7421	vitamin D (1,25-dihydroxyvitamin D3) receptor	2.07E-05	80.84922
AT225	1958	zinc finger protein 225	2.11E-05	80.67591
C/EBP-alph	1050	CCAAT/enhancer binding protein alpha	2.12E-05	80.5026
DKFZp586N	4088	mad homolog JV15-2	2.21E-05	80.32929
CD126	3570	interleukin 6 receptor alpha subunit	2.22E-05	80.06932
CD221	3480	insulin-like growth factor 1 receptor	2.22E-05	80.06932
ARNTL	406	basic-helix-loop-helix-PAS orphan MOP3	2.26E-05	79.80936
HOX-2.6	3214	homeobox B4	2.29E-05	79.63605
HES5	388585	hairy and enhancer of split 5 (Drosophila)	2.3E-05	79.46274
IRF3	3661	interferon regulatory factor 3	2.43E-05	79.28943
AUF1	3184	heterogeneous nuclear ribonucleoprotein D (AU-rich)	2.59E-05	79.11612
CD49f	3655	integrin alpha6B	2.67E-05	78.85615
CD104	3691	GP150	2.67E-05	78.85615
DKFZp686F	3611	integrin-linked kinase	2.72E-05	78.59619
MAZ	4150	zinc-finger protein, 87 kilodaltons	2.83E-05	78.42288
I-REL	5971	v-rel avian reticuloendotheliosis viral oncogene homolog B	3E-05	78.24957
ARA24	5901	ras-related nuclear protein	3.16E-05	77.9896
CHC1	1104	regulator of chromosome condensation 1	3.16E-05	77.9896
GIG34	6135	60S ribosomal protein L11	3.22E-05	77.72964
MGC13844	4772	nuclear factor of activated T-cells, cytoplasmic	3.26E-05	77.55633
E2F-1	1869	retinoblastoma-associated protein 1	3.26E-05	77.38302
FLJ94509	2672	zinc finger protein 163	3.34E-05	77.20971
DTR	1839	heparin-binding epidermal growth factor	3.38E-05	77.0364
BAPX2	4824	NK3 homeobox 1	3.44E-05	76.86308
AAP1	23429	ring1 interactor RYBP	3.44E-05	76.68977
C1	3183	heterogeneous nuclear ribonucleoprotein C (C1/C2)	3.48E-05	76.51646
BLIMP1	639	beta-interferon gene positive-regulatory domain I binding	3.58E-05	76.34315
EMT	3702	tyrosine-protein kinase ITK/TSK	3.86E-05	76.16984
E2F-6	1876	E2F transcription factor 6, isoform 1	3.9E-05	75.99653

BRCA1	672	breast and ovarian cancer susceptibility protein 1	4.12E-05	75.82322
MGC88021	5187	circadian pacemaker protein RIGUI	4.14E-05	75.64991
BCL3	602	B-cell leukemia/lymphoma 3	4.15E-05	75.4766
MAD2	4601	Max-related transcription factor	4.33E-05	75.30329
DKFZp434K	25942	transcriptional regulator, SIN3A	4.78E-05	75.12998
MSI1	4440	Musashi (Drosophila) homolog 1	4.85E-05	74.95667
ADRBK1	156	beta adrenergic receptor kinase 1	4.91E-05	74.78336
BORIS	140690	CCCTC-binding factor-like protein	5.02E-05	74.61005
Cmyb	4602	c-myb13A_CDS	5.05E-05	74.43674
GIG8	3398	cell growth-inhibiting gene 8	5.1E-05	74.26343
MGC11098	6622	non A4 component of amyloid	5.16E-05	74.09012
GEFT	115557	RAC/CDC42 exchange factor	5.25E-05	73.91681
ARNO	9266	cytohesin 2	5.27E-05	73.7435
DFNB24	5962	deafness, autosomal recessive 24	5.38E-05	73.57019
VAV2	7410	Protein vav-2	5.4E-05	73.39688
SREBF2	6721	sterol regulatory element-binding protein 2	5.69E-05	73.22357
CTS02	1513	cathepsin K	5.77E-05	73.05026
FLJ44809	2475	FKBP12-rapamycin complex-associated protein 1	5.89E-05	72.87695
RRAS	6237	Oncogene RRAS	5.96E-05	72.70364
H-ICSBP	3394	interferon consensus sequence binding protein 1	5.99E-05	72.53033
CDK4I	1030	cyclin-dependent kinases 4 and 6 binding protein	6.14E-05	72.35702
MGC13000	5058	p21/Cdc42/Rac1-activated kinase 1 (STE20 homolog, yeast)	6.16E-05	72.18371
BAG-6	7917	scythe	6.24E-05	72.0104
BAF190	6595	SWI/SNF-related matrix-associated actin-dependent	6.33E-05	71.83709
GP145-TrkI	4915	neurotrophic tyrosine kinase, receptor, type 2	6.39E-05	71.66378
ARVD12	3728	catenin (cadherin-associated protein), gamma (80kD)	6.4E-05	71.49047
DKFZp547C	25970	SH2B adaptor protein 1	6.4E-05	71.31716
GRF-1	2909	glucocorticoid receptor DNA binding factor 1	6.56E-05	71.14385
CSBP	3190	transformation upregulated nuclear protein	6.57E-05	70.97054
GNA12	2768	guanine nucleotide binding protein (G protein) alpha 12	6.58E-05	70.79723
ARNO3	9265	general receptor of phosphoinositides 1	6.7E-05	70.62392
APLP	333	amyloid-like protein 1	6.8E-05	70.45061
APKD2	5311	polycystic kidney disease 2 (autosomal dominant)	6.94E-05	70.2773
ANF	8820	homeobox, ES cell expressed 1	7.61E-05	70.01733
KIAA1047	9611	nuclear receptor co-repressor 1	7.61E-05	70.01733
KCIP-1	7534	phospholipase A2	7.62E-05	69.75737
ELK3	2004	SRF accessory protein 2	7.82E-05	69.58406
MGC13177	5970	v-rel avian reticuloendotheliosis viral oncogene homolog A	8.09E-05	69.41075
CTCF	10664	CTCFL paralog	8.16E-05	69.23744
CD295	3953	leptin receptor	8.44E-05	69.06412
DBK	5585	serine-threonine kinase N	8.57E-05	68.89081
NOV	5361	plexin 1	9.41E-05	68.7175
ASCL2	430	achaete-scute complex-like 2	9.6E-05	68.54419
GNG1	2792	guanine nucleotide binding protein (G protein)	9.68E-05	68.37088
BOS3	6495	SIX homeobox 1	0.0001	68.19757
CINC-2a	2920	chemokine (C-X-C motif) ligand 2	0.000101	68.02426
60B8AG	6279	cystic fibrosis antigen	0.000102	67.85095

MGC33401	7073	TIA1 cytotoxic granule-associated RNA-binding protein-like	0.000109	67.67764
MIZ-1	7709	zinc finger protein 151 (pHZ-67)	0.000112	67.50433
AAT5	7046	transforming growth factor beta receptor I	0.000114	67.33102
APRF	6774	DNA-binding protein APRF	0.000116	67.15771
CKBBP2	90480	papillomavirus L2 interacting nuclear protein 1	0.000116	66.9844
ALK-6	658	bone morphogenetic protein receptor, type IB	0.000117	66.81109
ASIP	56288	atypical PKC isotype-specific interacting protein	0.000127	66.63778
EZF	9314	endothelial Kruppel-like zinc finger protein	0.000128	66.46447
DOCK1	1793	dedicator of cyto-kinesis 1	0.00013	66.11785
DOCK2	1794	dedicator of cytokinesis 2	0.00013	66.11785
CED-12	9844	engulfment and cell motility 1	0.00013	66.11785
MGC10556	7205	zyxin related protein 1	0.000142	65.77123
C1Q-C	714	complement component 1, q subcomponent, C chain	0.000149	65.42461
C1QB	713	complement component 1, q subcomponent	0.000149	65.42461
C1QA	712	complement component C1q, A chain	0.000149	65.42461
FLJ20819	7879	Ras-associated protein RAB7	0.000151	65.07799
MGC11118	5645	trypsin 2	0.000159	64.73137
MTG	5646	trypsin 3	0.000159	64.73137
MGC12017	5644	nonfunctional trypsin 1	0.000159	64.73137
ARF-BP1	10075	ARF binding protein 1	0.000163	64.38475
FKHL16	2305	Forkhead, drosophila, homolog-like 16	0.000165	64.21144
FLJ41865	26230	T-cell lymphoma invasion and metastasis 2	0.00017	63.95147
FARP2	9855	FGD1-related Cdc42-GEF	0.00017	63.95147
ERBB2IP	55914	ERBB2 interacting protein	0.000177	63.69151
HDAC2	3066	YY1-associated factor 1	0.000177	63.5182
MKNK1	8569	MAP kinase interacting serine/threonine kinase 1	0.000178	63.34489
DKFZp313L	2934	gelsolin (amyloidosis, Finnish type)	0.000181	63.17158
ANCR	7337	ubiquitin protein ligase E3A	0.000183	62.99827
PTP-PEST	5782	protein-tyrosine phosphatase G1	0.000184	62.82496
MTA1	9112	metastasis associated protein	0.000185	62.65165
MAP3K11	4296	mitogen-activated protein kinase kinase kinase 11	0.000187	62.47834
BBC3	27113	BCL2 binding component 3	0.000187	62.30503
JNK	5599	stress-activated protein kinase JNK1	0.000189	62.13172
BID	637	BID isoform Si6	0.00019	61.95841
BAM	10018	bcl-2 interacting protein Bim	0.000192	61.7851
ARNT	405	dioxin receptor, nuclear translocator	0.000195	61.61179
FKBP38	23770	FK506 binding protein 8, 38kDa	0.000195	61.43847
DKFZp686C	5148	rod cG-PDE G	0.000195	61.17851
CSNBAD3	2779	transducin alpha-1 chain	0.000195	61.17851
ARHGEF14	23263	MCF2 transforming sequence-like protein	0.000205	60.91854
FLJ16302	4855	Notch homolog 4 (Drosophila)	0.00021	60.74523
GCN5	2648	GCN5 general control of amino-acid synthesis 5-like 2	0.00021	60.57192
AG2S	185	angiotensin receptor 1B	0.000213	60.39861
CD281	7096	Toll/interleukin-1 receptor-like	0.000216	60.2253
ARHGEF2	9181	rho/rac guanine nucleotide exchange factor (GEF) 2	0.000218	60.05199
ATOD4	9021	suppressor of cytokine signaling 3	0.00022	59.87868
ARH6	388	oncogene RHO H6	0.000222	59.70537

AT-V1	4683	p95 protein of the MRE11/RAD50 complex	0.000233	59.44541
H2A.X	3014	H2AX histone	0.000233	59.44541
CALNB1	5534	protein phosphatase 3, regulatory subunit B, alpha isoform	0.00024	59.09879
PPP3R2	5535	protein phosphatase 3, regulatory subunit B (calcineurin B)	0.00024	59.09879
ACVRIP1	9863	membrane associated guanylate kinase	0.00025	58.83882
2-5-3p	23265	exocyst complex component 7	0.000256	58.66551
CRES	10047	cystatin-related epididymal spermatogenic protein	0.000259	58.40555
NEC2	5126	subtilisin-like prohormone convertases	0.000259	58.40555
AR1	6942	stromelysin-1 platelet-derived growth factor-responsive	0.00026	58.14558
CD124	3566	interleukin-4 receptor alpha chain	0.000264	57.88562
CD132	3561	common cytokine receptor gamma chain	0.000264	57.88562
KIAA0407	5364	plexin B1	0.000268	57.62565
HsT18964	22801	integrin, alpha 11	0.000275	57.36568
CD29	3688	integrin beta 1	0.000275	57.36568
CIS1	8651	STAT induced SH3 protein 1	0.000276	57.10572
DOCK7	85440	dedicator of cytokinesis 7	0.000277	56.93241
NF-E2	4778	nuclear factor (erythroid-derived 2), 45kDa	0.000281	56.7591
ACTRIB	91	serine(threonine) protein kinase receptor R2	0.000281	56.58579
DVL2	1856	dishevelled 2	0.000282	56.41248
DKFZp547J	1995	Hu antigen C	0.00029	56.23917
ANCCA	29028	ATPase family, AAA domain containing 2	0.000294	56.06586
DKFZp781I	4914	neurotrophic tyrosine kinase, receptor, type 1	0.000306	55.89255
CD120a	7132	tumor necrosis factor receptor type 1	0.000312	55.71924
CCND2	894	cyclin D2	0.000321	55.54593
MGC12953	6097	nuclear receptor ROR-gamma	0.000322	55.37262
FKLF	8462	Kruppel-like factor 11	0.000339	55.19931
BCGF-1	3565	B cell growth factor 1	0.000343	55.026
DKFZp781F	80314	enhancer of polycomb 1	0.000344	54.85269
ABC-1	19	ATP binding cassette transporter 1	0.000346	54.59272
GIF	4282	phenylpyruvate tautomerase	0.000346	54.59272
DBL	4168	Oncogene MCF2 (oncogene DBL)	0.000348	54.33276
CRS2	4488	msh homeobox 2	0.00035	54.15945
CK2A2	1459	casein kinase 2, alpha prime polypeptide	0.00035	53.98614
VASP	7408	vasodilator-stimulated phosphoprotein	0.000357	53.81282
CUL4B	8450	cullin-4B	0.000358	53.63951
CP107	5933	retinoblastoma-like 1 (p107)	0.000363	53.4662
ACTRIIB	93	activin A type IIB receptor	0.000382	53.29289
CHNG1	7253	thyroid stimulating hormone receptor	0.000386	53.11958
IRF4	3662	interferon regulatory factor 4	0.000387	52.94627
FLJ12850	57650	KIAA1524	0.000392	52.77296
MGC41878	5579	protein kinase C, beta 1	0.000394	52.59965
DUET	8997	serine/threonine kinase with Dbl- and pleckstrin homology	0.0004	52.42634
IRAK	3654	interleukin-1 receptor-associated kinase 1	0.000401	52.25303
ARHGEF7	8874	rho guanine nucleotide exchange factor 7	0.000407	52.07972
ERK	5594	protein tyrosine kinase ERK2	0.000413	51.81976
ERK1	5595	extracellular signal-regulated kinase 1	0.000413	51.81976
CRC18	1630	deleted in colorectal cancer protein	0.000432	51.55979

CARD3	8767	CARD-containing interleukin-1 beta-converting enzyme	0.000442	51.38648
ARHG	391	ras homolog gene family, member G (rho G)	0.000446	51.03986
GDS1	5910	RAP1, GTP-GDP dissociation stimulator 1	0.000446	51.03986
DOCK4	9732	dedicator of cytokinesis 4	0.000446	51.03986
BNSP	6696	SPP1/CALPHA1 fusion	0.000464	50.69324
60B8AG	6280	calgranulin B	0.000466	50.51993
DVL3	1857	dishevelled 3	0.000467	50.34662
DKFZp686C	8848	transforming growth factor beta-stimulated protein TSC-22	0.000487	50.17331
BCAR1	9564	Cas scaffolding protein family member 1	0.000488	50
B56A	5525	serine/threonine protein phosphatase 2A, 56 kDa regulatory	0.000488	49.82669
NMI	9111	N-myc-interactor	0.000512	49.65338
BM600	3909	epiligrin alpha 3 subunit	0.000529	49.39341
CD49C	3675	very late activation protein 3 receptor, alpha-3 subunit	0.000529	49.39341
CDCD1	4000	limb girdle muscular dystrophy 1B (autosomal dominant)	0.000529	49.13345
CSK	1445	c-src tyrosine kinase	0.000532	48.96014
FLJ25655	5885	nuclear matrix protein 1	0.000541	48.70017
ESP1	9700	extra spindle poles like 1	0.000541	48.70017
ARHGAP5	394	p100 RasGAP-associated p105 protein	0.000553	48.44021
ECK	1969	protein tyrosine kinase	0.000556	48.2669
GPRC1E	2915	glutamate receptor, metabotropic 5	0.000571	48.09359
GIOT-1	92283	zinc finger protein 461	0.000584	47.92028
MGC12565	5581	protein kinase C, epsilon	0.000595	47.74697
APBB1	322	amyloid beta (A4) precursor protein-binding, family B	0.000598	47.57366
ARID1A	8289	SWI/SNF related, matrix associated	0.000609	47.40035
KIAA1119	4645	myosin VB	0.000612	47.22704
APRO6	10140	transducer of ERBB2, 1	0.000615	47.05373
DKFZp686A	3146	high-mobility group (nonhistone chromosomal) protein 1	0.00062	46.79376
HMG2	3148	high-mobility group box 2	0.00062	46.79376
FLJ97193	10023	proto-oncogene FRAT1	0.000629	46.5338
ACVRL1	94	activin A receptor, type II-like kinase 1	0.000662	46.36049
156DAG	1605	dystrophin-associated glycoprotein-1	0.000671	46.18718
MAF	4094	Avian musculoaponeurotic fibrosarcoma (MAF)	0.000673	46.01386
2-Oct	5452	POU domain, class 2, transcription factor 2	0.000679	45.84055
GH	2688	pituitary growth hormone	0.000734	45.66724
ACTA	58	alpha skeletal muscle actin	0.000747	45.49393
CMD2A	7137	troponin I type 3 (cardiac)	0.000751	45.23397
ACTC	70	cardiac muscle alpha actin 1	0.000751	45.23397
CMD1G	7273	titin	0.000756	44.974
TSG10	7251	tumor susceptibility gene 101	0.000767	44.80069
CD49e	3678	integrin, alpha 5 (fibronectin receptor, alpha polypeptide)	0.000773	44.62738
ITSN	6453	intersectin short variant 12	0.000797	44.45407
LUN	10210	OTTHUMP00000045227	0.000801	44.28076
C-Rel	5966	v-rel reticuloendotheliosis viral oncogene homolog	0.000827	44.10745
DMP1	9988	cyclin D binding myb-like transcription factor 1	0.000831	43.93414
CDHS	5077	Waardenburg syndrome 1	0.00084	43.76083
AMY-1	26292	associate of myc-1	0.000866	43.58752
CDCA7L	55536	transcription factor RAM2	0.000871	43.32756

CDK8	1024	CDK8 protein kinase	0.000871	43.32756
CD63	967	CD63 antigen (melanoma 1 antigen)	0.000879	43.06759
DKFZp781F	5325	PLAG-like 1	0.000881	42.89428
B2T	3918	laminin, gamma 2 (nicein (100kD), kalinin (105kD))	0.000905	42.72097
CNBP	7555	zinc finger protein 9 (a cellular retroviral nucleic acid)	0.00091	42.54766
G13	10672	guanine nucleotide binding protein (G protein), alpha 13	0.000931	42.37435
CD18	3689	complement receptor C3 beta-subunit	0.000933	42.02773
FGA	2243	fibrinogen, alpha polypeptide	0.000933	42.02773
CD11C	3687	myeloid membrane antigen, alpha subunit	0.000933	42.02773
DKFZp686L	7342	upstream binding protein 1 (LBP-1a)	0.000949	41.68111
BSF3	23529	B-cell stimulating factor 3	0.000963	41.5078
TLR7	51284	toll-like receptor 7	0.000967	41.33449
Hs.89862	8717	tumor necrosis factor receptor type 1 associated death	0.000979	41.16118
KIAA1907	3911	laminin alpha-5 chain	0.000982	40.98787
ARFGEP10(	9922	IQ motif and Sec7 domain 1	0.001017	40.81456
AOF2	23028	lysine (K)-specific demethylase 1	0.001019	40.64125
ALK	238	anaplastic lymphoma kinase Ki-1	0.001046	40.46794
FLJ94114	3952	obesity factor	0.001048	40.29463
AMPK	5563	AMP-activated protein kinase alpha 2 catalytic subunit	0.001057	40.03466
AMPK	5562	AMPK alpha 1	0.001057	40.03466
CD51	3685	integrin alpha-V	0.001068	39.7747
SNAPAP	23557	SNARE associated protein snapin	0.001089	39.51473
DKFZp434K	7442	transient receptor potential vanilloid 1b	0.001089	39.51473
CBBM	5956	opsin 1 (cone pigments), long-wave-sensitive (protan)	0.001101	39.25477
KIAA0988	6904	tubulin-specific chaperone d	0.001102	39.08146
IRF5	3663	interferon regulatory factor 5	0.001118	38.90815
DELTA1	28514	delta-like 1	0.001135	38.73484
EDF	3624	inhibin, beta A (activin A, activin AB alpha polypeptide)	0.001136	38.47487
BMP-11	10220	growth differentiation factor 11	0.001136	38.47487
ACTN2	88	F-actin cross-linking protein	0.001192	38.2149
ACC-4	1512	cathepsin H	0.001203	38.04159
CCL11	6356	small inducible cytokine A11	0.001209	37.86828
CC-CKR-2	1231	monocyte chemoattractant protein 1 receptor	0.001221	37.69497
CCL24	6369	eotaxin-2	0.001237	37.52166
CADASIL	4854	Notch homolog 3 (Drosophila)	0.001267	37.34835
ACTRI	90	activin A receptor, type II-like kinase 2	0.001279	37.17504
GDF8	2660	myostatin	0.00129	37.00173
DIF	7124	TNF, monocyte-derived	0.001307	36.82842
ACVR1C	130399	activin receptor-like kinase 7	0.001325	36.65511
DDX9	1660	DEAD/H box-9 (nuclear DNA helicase II; RNA helicase A)	0.001328	36.4818
CFND	1947	ephrin-B1	0.001354	36.30849
ALPS1A	355	Fas (TNF receptor superfamily, member 6)	0.001371	36.13518
DKFZp313F	5791	protein tyrosine phosphatase epsilon	0.001399	35.96187
CD44	960	chondroitin sulfate proteoglycan 8	0.001405	35.78856
BMP2	650	bone morphogenetic protein 2	0.001453	35.61525
H2A.z	3015	H2AZ histone	0.001463	35.44194
ABRI	9445	BRICHOS domain containing 2B	0.001497	35.26863

CTSS	1520	cathepsin S	0.001528	35.09532
CCNB	891	G2/mitotic-specific cyclin B1	0.001554	34.92201
DAPK3	1613	ZIP kinase isoform	0.001557	34.7487
ECP54	8607	RuvB (E coli homolog)-like 1	0.001594	34.57539
MGC21659	7069	spot 14 protein	0.001618	34.40208
AIM	1786	DNA (cytosine-5-)-methyltransferase 1	0.001627	34.22877
MST155	51429	SH3 and PX domain-containing protein SH3PX1	0.001647	34.05546
MAD7	6945	transcription factor-like 4	0.001651	33.88215
ACPA	5657	proteinase 3 (serine proteinase, neutrophil)	0.001654	33.70884
DNMT3A	1788	DNA (cytosine-5-)-methyltransferase 3 alpha	0.001686	33.53553
MBP	4155	myelin basic protein	0.001738	33.36222
ADAM33	80332	a disintegrin and metalloprotease 33	0.001744	33.18891
IB1	9479	mitogen-activated protein kinase 8 interacting protein 1	0.001755	33.0156
ASEF2	221178	adenomatous polyposis coli stimulated exchange factor 2	0.001796	32.84229
ACTB	60	PS1TP5-binding protein 1	0.001811	32.66898
CLASPIN	63967	claspin	0.001812	32.32236
BETA-TRCP	8945	beta-TrCP1	0.001812	32.32236
ARHGEF4	50649	Rho guanine nucleotide exchange factor 4	0.001812	32.32236
CXCL12	6387	chemokine (C-X-C motif) ligand 12 (stromal cell-derived)	0.001845	31.97574
EMC19	6500	transcription elongation factor B (SIII), polypeptide 1-like	0.001851	31.80243
PI5	5268	serpin peptidase inhibitor, clade B (ovalbumin), member 5	0.001882	31.62912
ADAP1	11033	centaurin-alpha	0.001944	31.45581
AFP	174	alpha-1-fetoprotein	0.001965	31.2825
ICK	22858	MAK-related kinase	0.001969	31.10919
CCL7	6354	chemokine (C-C motif) ligand 7	0.002011	30.93588
CCL3	6348	small inducible cytokine A3 (homologous to mouse Mip-1a)	0.002096	30.5026
464.2	6349	small inducible cytokine A3-like 1	0.002096	30.5026
464.2	414062	small inducible cytokine A3-like 1	0.002096	30.5026
CCL2	6347	small inducible cytokine A2	0.002096	30.5026
APRIL	8741	tumor necrosis factor-related death ligand-1	0.002114	30.06932
CAPRIN1	4076	membrane component chromosome 11 surface marker 1	0.002129	29.89601
ACT	72	alpha-actin 3	0.002136	29.63605
AAT6	59	actin, alpha 2, smooth muscle, aorta	0.002136	29.63605
BMP2B	652	bone morphogenetic protein 4	0.002192	29.37608
ABI-1	10006	nap1 binding protein	0.002193	29.20277
NR2C2	7182	nuclear receptor subfamily 2, group C, member 2	0.002236	29.02946
AGER	177	advanced glycosylation end product-specific receptor	0.002292	28.85615
EHK3	2045	Eph homology kinase-3	0.002313	28.68284
ARH	26119	LDL receptor adaptor protein	0.002327	28.50953
FLJ22252	64321	SRY (sex determining region Y)-box 17	0.002345	28.33622
AR-JP	5071	parkin	0.002378	28.16291
COL1A1	1277	alpha 1 type I collagen	0.002445	27.9896
APOA1	335	apolipoprotein A1	0.00247	27.81629
DKFZp762F	55662	hypoxia-inducible factor 1, alpha subunit inhibitor	0.002484	27.64298
EFNB2	1948	ephrin B2	0.002486	27.46967
FLOT1	10211	flotillin 1	0.002615	27.29636
CD2AP	23607	CD2-associated protein	0.002658	27.12305

H1	6013	prorelaxin	0.00266	26.94974
B2-1	9267	homolog of secretory protein SEC7	0.002666	26.77643
BGP	634	biliary glycoprotein adhesion molecule	0.00274	26.60312
RNF81	6737	Sjogren syndrome antigen A1 (52kDa, ribonucleoprotein)	0.002743	26.42981
CD344	8322	WNT receptor frizzled-4	0.002922	26.2565
MGC5329	5204	prefoldin subunit 5	0.002923	26.08319
ATX1	6310	ataxin 1	0.002948	25.90988
ELF5	2001	epithelium-specific Ets transcription factor 2	0.002986	25.73657
C7orf16	10842	G-substrate	0.003075	25.56326
HOP	10963	stress-induced-phosphoprotein 1 (Hsp70/Hsp90-organizing)	0.0031	25.38995
DKFZp686J	57496	myocardin-related transcription factor B	0.003136	25.21664
CAP	10580	sorbin and SH3 domain containing 1	0.003198	25.04333
COL4A3	1285	alpha 3 type IV collagen	0.003207	24.87002
BSAC	57591	megakaryocytic acute leukemia	0.003223	24.69671
GHRF	2691	somatocrinin	0.003389	24.5234
AGS1	51655	dexamethasone-induced ras-related protein 1	0.003553	24.35009
CLEVER-1	23166	common lymphatic endothelial and vascular endothelial	0.003555	24.09012
ON	6678	secreted protein, acidic, cysteine-rich	0.003555	24.09012
IHPS1	4842	neuronal nitric oxide synthase	0.003574	23.7435
SSTR5	6755	somatostatin receptor subtype 5	0.003574	23.7435
MGC13823	4838	nodal, mouse, homolog	0.003729	23.48354
DKFZp586D	9477	Trf (TATA binding protein-related factor)-proximal homolog (Droso	0.00384	23.31023
KDP	65125	serine/threonine-protein kinase WNK1	0.003917	23.13692
LGMD1	9499	limb-girdle muscular dystrophy 1A (autosomal dominant)	0.004037	22.9636
PFM2	11107	PR domain containing 5	0.004052	22.79029
APT1LG1	356	tumor necrosis factor (ligand) superfamily, member 6	0.004175	22.61698
CCT-alpha	6950	T-complex protein 1, alpha subunit	0.004197	22.44367
ACAN	176	large aggregating proteoglycan	0.004198	22.27036
DLL4	54567	notch ligand DLL4	0.004266	22.09705
FLJ23903	4641	nuclear myosin I	0.004269	21.92374
GSC	145258	goosecoid	0.004346	21.75043
C15	51316	placenta-specific 8	0.004452	21.57712
A-FABP	2167	fatty acid binding protein 4, adipocyte	0.004471	21.40381
HsMAD1	8379	tumor protein p53 inducible protein 9	0.004734	21.2305
IB2	23542	mitogen-activated protein kinase 8 interacting protein 2	0.004736	21.05719
FCER1G	2207	Fc fragment of IgE, high affinity I, receptor	0.00476	20.88388
ALPS2	843	caspase 10	0.00478	20.71057
BMP7	655	osteogenic protein 1	0.005046	20.53726
HOX7	4487	msh homeobox 1	0.005049	20.36395
FLJ20922	5900	ral guanine nucleotide dissociation stimulator	0.005162	20.19064
CLPS	1208	pancreatic colipase preproprotein	0.005333	20.01733
BAZ1B	9031	Williams-Beuren syndrome chromosome region 10	0.005344	19.84402
APBA1	320	amyloid beta A4 precursor protein-binding, family A	0.005354	19.67071
KIAA1303	57521	regulatory associated protein of mTOR	0.005428	19.4974
FH	3949	low density lipoprotein receptor	0.005549	19.32409
AMPH	273	Stiff-Man syndrome with breast cancer 128kDa autoantigen	0.00572	19.15078
CSCD	1634	small leucine-rich protein 1B	0.005771	18.97747

FLJ21396	23132	RAD54-like 2	0.005876	18.80416
87U6	2773	guanine nucleotide binding protein (G protein)	0.005918	18.54419
GNAI1	2770	Gi1 protein alpha subunit	0.005918	18.54419
DKFZp686J	4763	neurofibromin	0.006012	18.28423
ERRL1	133522	peroxisome proliferator-activated receptor gamma	0.006018	18.11092
DKFZp779N	5340	plasminogen	0.006244	17.93761
CNR2	56142	KIAA0345-like 8	0.006278	17.7643
CNR1	56144	ortholog of mouse CNR1	0.006313	17.59099
CD350	11211	frizzled 10	0.006334	17.41768
CMT2A	9927	mitofusin-2	0.006396	17.24437
FLJ93058	4688	neutrophil cytosolic factor 2 (65kD, chronic granulomatous)	0.006525	17.07106
HSP70-1	3303	heat shock-induced protein	0.00674	16.81109
HSP70-1B	3304	heat shock 70kD protein 1B	0.00674	16.81109
COE3	253738	early B-cell factor 3	0.006794	16.55113
14-3-3	10971	14-3-3 protein T-cell	0.006857	16.37782
HRMT1L3	10196	protein arginine methyltransferase 3	0.006923	16.20451
ELF3	1999	E74-like factor 3 (ets domain transcription factor)	0.006941	15.77123
EST01027	10628	thioredoxin interacting protein	0.006941	15.77123
ERF	2077	Ets2 repressor factor	0.006941	15.77123
HHO.C10	3215	homeo box B5	0.006941	15.77123
CASP14	23581	caspase 14	0.007346	15.33795
CC10	7356	Uteroglobin (Clara-cell specific 10-kD protein)	0.00756	15.16464
CRFR2	1395	corticotropin releasing hormone receptor 2	0.00815	14.90468
CRF-R	1394	seven transmembrane helix receptor	0.00815	14.90468
BMP6	654	bone morphogenetic protein 6	0.008197	14.64471
NKD2	85409	Dvl-binding protein NKD2	0.008331	14.4714
OPMD	8106	poly(A) binding protein, nuclear 1	0.008797	14.29809
BM600-125	3914	laminin B1k chain	0.009055	14.12478
DKFZp564E	5861	RAB1, member RAS oncogene family	0.009073	13.95147
ASD3	4624	myosin heavy chain, cardiac muscle alpha isoform	0.009091	13.77816
E14	4863	nuclear protein, ataxia-telangiectasia locus	0.010133	13.60485
FLJ23079	6616	synaptosomal-associated protein 25	0.010299	13.43154
FLJ11460	6844	synaptobrevin 2	0.010355	13.25823
CAPB	832	capping protein (actin filament) muscle Z-line, beta	0.011159	13.08492
LSP1	4046	F-actin binding and cytoskeleton associated protein	0.011209	12.91161
MGC16699	5797	protein tyrosine phosphatase, receptor type	0.011433	12.65165
CD113	25945	poliovirus receptor-related 3	0.011433	12.65165
DKFZp686C	4703	nebulin	0.011442	12.39168
HGS	9146	hepatocyte growth factor-regulated tyrosine kinase	0.011562	12.21837
CACH2	775	voltage-gated calcium channel alpha subunit Cav1.2	0.011945	12.04506
CLTA	1211	clathrin, light chain (Lca)	0.012094	11.7851
CLTB	1212	clathrin, light polypeptide	0.012094	11.7851
CAB1	782	dihydropyridine-sensitive L-type, calcium channel beta-1	0.012667	11.52513
HMOX2	3163	heme oxygenase (decycling) 2	0.012731	11.26516
CACNB2	783	calcium channel, voltage-dependent, beta 2 subunit	0.012731	11.26516
BMP14	8200	cartilage-derived morphogenetic protein-1	0.013325	11.0052
CFD	200576	1-phosphatidylinositol-4-phosphate 5-kinase	0.013835	10.74523

HSF2	3298	heat shock transcription factor 2	0.013835	10.74523
AT3	4287	Machado-Joseph disease protein 1	0.013974	10.48527
CACNB3	784	calcium channel, voltage-dependent, beta 3 subunit	0.014161	10.31196
CHRD	8646	chordin	0.014237	10.13865
CD213A1	3597	interleukin 13 receptor, alpha 1	0.014717	9.965338
HDLCQ12	3990	lipase C	0.015226	9.792028
BCD541	6606	survival of motor neuron 1, telomeric	0.015544	9.445407
FBP2	8570	KH-type splicing regulatory protein (FUSE binding protein 2)	0.015544	9.445407
BCD541	6607	survival of motor neuron 2, centromeric	0.015544	9.445407
FGG	2266	fibrinogen, gamma polypeptide	0.015735	9.098787
BP	3240	binding peptide	0.016233	8.925477
SATB1	6304	special AT-rich sequence binding protein 1	0.01658	8.752166
Fug1	5905	Ran GTPase activating protein 1	0.017234	8.578856
MAFF	23764	transcription factor MAFF	0.018307	8.405546
MGC:8389	9519	TBP-related factor 2	0.018393	8.232236
ERK3	6300	stress-activated protein kinase 3	0.01855	8.058925
HPK38	9833	pEg3 kinase	0.018968	7.885615
ZABC1	7764	zinc finger protein 217	0.02033	7.712305
MESP1	55897	mesoderm posterior 1 homolog (mouse)	0.020681	7.45234
12CC4	27086	glutamine-rich factor 1	0.020681	7.45234
MEL-18	7703	zinc finger protein 144	0.020939	7.192374
ADAP	2533	FYN-binding protein (FYB-120/130)	0.021077	7.019064
FLJ27265	3150	high-mobility group (nonhistone chromosomal) protein 14	0.021171	6.845754
ADRA2	150	alpha2A adrenergic receptor	0.021562	6.672444
Bx42	22938	nuclear protein SkiP	0.022197	6.499133
ARNT2	9915	aryl-hydrocarbon receptor nuclear translocator 2	0.023026	6.325823
ARHGAP7	10395	START domain containing protein 12	0.023127	6.065858
RAB10	10890	ras-related GTP-binding protein RAB10	0.023127	6.065858
ARHGAP26	23092	GTPase regulator associated with the focal adhesion kinase	0.023237	5.805893
APOB	338	apolipoprotein B48	0.024973	5.632582
ERBA-BETA	7068	generalized resistance to thyroid hormone	0.026373	5.372617
AR7	7067	triiodothyronine receptor	0.026373	5.372617
MSK	150094	myocardial SNF1-like kinase	0.026568	5.112652
CYT4	27128	cytohesin-4	0.027428	4.939341
FLJ33906	80317	zinc finger with KRAB and SCAN domains 3	0.027481	4.766031
ATX	5168	autotaxin	0.027892	4.592721
CMD1P	5350	phospholamban	0.029516	4.332756
ATP2A2	488	sarcoplasmic/endoplasmic reticulum calcium ATPase 2	0.029516	4.332756
CARD12	58484	caspase recruitment domain family, member 12	0.033923	4.07279
INPP5D	3635	signaling inositol polyphosphate 5 phosphatase SIP-145	0.034119	3.89948
CDP2	23316	cut-like 2	0.034235	3.379549
CSF2	1437	granulocyte-macrophage colony stimulating factor	0.034235	3.379549
HACE1	57531	HECT domain and ankyrin repeat containing, E3 ubiquitin	0.034235	3.379549
GASP	9737	G protein-coupled receptor-associated sorting protein	0.034235	3.379549
IPOA4	3839	importin-alpha-Q2	0.034235	3.379549
K17	3872	keratin 17	0.03716	2.859619
CAP-3	5272	protease inhibitor 9 (ovalbumin type)	0.037266	2.686308

CD301	10462	macrophage C-type lectin	0.038195	2.512998
CCNE2	9134	cyclin E2	0.038288	2.339688
BS4	51667	NY-REN-18 antigen	0.038288	2.166378
FLJ12615	64398	MAGUK p55 subfamily member 5	0.038544	1.993068
DMP-1	1758	dentin matrix acidic phosphoprotein 1	0.040576	1.819757
GPR7	2831	opioid-somatostatin-like receptor 7	0.042203	1.559792
GPR8	2832	neuropeptides B/W receptor 2	0.042203	1.559792
N-syndecar	9672	syndecan proteoglycan 3	0.044542	1.299827
MZF-1	7593	zinc finger protein 42	0.045195	1.126516
DLG3	1741	synapse-associated protein 102	0.046249	0.953206
BGR	64581	C-type lectin	0.046873	0.779896
CD266	51330	type I transmembrane protein Fn14	0.046897	0.606586
HOX11L2	30012	T-cell leukemia, homeobox 3	0.047972	0.433276
EB3	22924	microtubule-associated protein, RP/EB family, member 3	0.048469	0.259965
FLH2	5551	lymphocyte pore forming protein	0.049863	0.086655
