# Supplementary material for: “Topological Significance” Analysis of Gene Expression and Proteomic Profiles from Prostate Cancer Cells Reveals Key Mechanisms of Androgen Response
Source: PLoS One. 2010 Jun 3;5(6):e10936. doi: 10.1371/journal.pone.0010936 (PMC2880599; doi:10.1371/journal.pone.0010936)
Supplement: Table S3 — Enrichment analysis of up-regulated genes and proteins. The list of pathway maps and GeneGo process networks significantly enriched in up-regulated genes and proteins. An FDR threshold of 0.05 was used for maps and 0.2 for networks. (0.07 MB PDF) [file pone.0010936.s003.pdf]

List of maps and process networks enriched in up-regulated genes

**Maps**		**Process networks**	
Unsaturated fatty acid biosynthesis	5.56E-07	Apoptosis_Endoplasmic reticulum stress pathway	0.00137
Glycine, serine, cysteine and threonine metabolism/ Rodent version	1.62E-05	Signal transduction_Leptin signalig	0.00215
Glycine, serine, cysteine and threonine metabolism	1.62E-05	Signal transduction_CREM pathway	0.00378
Aminoacyl-tRNA biosynthesis in cytoplasm/Roden	2.47E-05	Signal transduction_Insulin signaling	0.00591
Aminoacyl-tRNA biosynthesis in cytoplasm	2.47E-05	Proliferation_Positive regulation cell proliferation	0.00855
n-3 Polyunsaturated fatty acid biosynthesis	4.07E-05	Cytoskeleton_Intermediate filaments	0.00875
n-6 Polyunsaturated fatty acid biosynthesis	4.07E-05		
Urea cycle	6.37E-05		
Development_Growth hormone signaling via PI3K/AKT and	0.000472		
Arachidonic acid production	0.00159		
Proline metabolism	0.0019		
Transcription_Receptor-mediated HIF regulation	0.002		

List of maps and process networks enriched in up-regulated proteins

**Maps**		**Process networks**	
Aminoacyl-tRNA biosynthesis in cytoplasm/ Rodent version	4.51E-09	Translation_Elongation-Termination	2.61E-17
Aminoacyl-tRNA biosynthesis in cytoplasm	4.51E-09	Translation_Translation initiation	1.03E-10
Phenylalanine metabolism/ Rodent version	0.000122	Signal transduction_Insulin signaling	0.000344
Phenylalanine metabolism	0.000122	Response to hypoxia and	0.00501
Regulation of lipid metabolism_Insulin	0.000337	Protein folding_Folding in normal condition	0.00613
Regulation of lipid metabolism_Insulin regulation of fatty acid	0.000413	Signal transduction_Androgen receptor nuclear signaling	0.00737
Glycine, serine, cysteine and threonine metabolism	0.000441		
Glycine, serine, cysteine and threonine [illegible]	0.0005		
Apoptosis and survival_Anti-apoptotic action of nuclear ESR1 and	0.00317		
Oxidative stress_Role of ASK1 under oxidative	0.00206		
Aspartate and asparagine metabolism	0.00206		
Cell cycle_Role of 14-3-3 proteins in cell cycle regulation	0.00227		
Development_Lipoxin inhibitory action on PDGF, EGF and LTD4 signaling	0.0027		
Inhibitory action of Lipoxin A4 on PDGF, EGF and LTD4 signaling	0.0027		
