# Supplementary material for: “Topological Significance” Analysis of Gene Expression and Proteomic Profiles from Prostate Cancer Cells Reveals Key Mechanisms of Androgen Response
Source: PLoS One. 2010 Jun 3;5(6):e10936. doi: 10.1371/journal.pone.0010936 (PMC2880599; doi:10.1371/journal.pone.0010936)
Supplement: Table S4 — Enrichment analysis of randomly extended up-regulated genes and proteins. In order to investigate whether significant differences in sizes of the sets used in our analysis could have affected the results we randomly sampled the pool of genes and proteins and added them to the differentially expressed sets. (0.07 MB PDF) [file pone.0010936.s004.pdf]

Enrichment of extended up-regulated genes

Maps			Process networks	
	Average # of significant maps (FDR of 0.05)	# of new and original significant maps recovered*	Average # of significant processes (FDR of 0.2)	# of new and original significant processes recovered*
Original set (no random added)	12	0 and 12	6	0 and 6
50 random	9.58	1 and 11	3.2	4 and 6
100 random	9.7	0 and 11	3.1	4 and 6
200 random	8.8	1 and 11	2.1	0 and 4
300 random	8.8	0 and 8	1.4	0 and 3
400 random	3.7	0 and 9	0.9	0 and 2
500 random	2.3	0 and 7	0.9	0 and 1
1,000 random	0.3	0 and 0	0.5	0 and 0

100 randomizations were performed for each size of random gene set

*Only maps significant in at least 10% of randomizations were counted

Enrichment of extended up-regulated proteins

	Maps		Process networks	
	Average # of significant maps (FDR of 0.05)	# of new and original significant maps recovered*	Average # of significant processes (FDR of 0.2)	# of new and original significant processes recovered*
Original set (no random added)	14	1 and 14	6	0 and 6
50 random	7.28	0 and 8	4.9	1 and 6
100 random	3.55	0 and 2	5.1	4 and 6
200 random	3.2	0 and 2	3.4	0 and 6
300 random	2.7	0 and 2	3.9	0 and 4
400 random	2.7	0 and 2	3.5	0 and 3
500 random	1.42	0 and 2	2.8	0 and 2
1,000 random	0.26	0 and 0	2.3	0 and 2

100 randomizations were performed for each size of random gene set

*Only maps significant in at least 10% of randomizations were counted
