# Supplementary material for: “Topological Significance” Analysis of Gene Expression and Proteomic Profiles from Prostate Cancer Cells Reveals Key Mechanisms of Androgen Response
Source: PLoS One. 2010 Jun 3;5(6):e10936. doi: 10.1371/journal.pone.0010936 (PMC2880599; doi:10.1371/journal.pone.0010936)
Supplement: Table S5 — List of topologically significant genes and proteins determined from truncated sets of up-regulated genes and proteins. We removed the direct targets of androgen receptor from the list of up-regulated genes and proteins. Direct targets were determined as those proteins to which androgen receptor has a direct “transcription regulation” type of interaction. The resulting truncated sets were re-analyzed with the topological significance tool and 565 significant proteins and 668 significant genes identified (FDR<5%). (0.43 MB PDF) [file pone.0010936.s005.pdf]

Topologically significant genes from truncated set of gene expression

Symbol	Entrez_ID	Description	p value	percentile
PKC-MU	5587	protein kinase D1	1.56E-10	99.8503
CREB	1385	cAMP responsive element binding protein 1	3.36E-10	99.77545
ATF4	468	activating transcription factor 4	3.59E-09	99.62575
PS6K	6198	serine/threonine kinase 14 alpha	3.95E-09	99.47605
SREBF1	6720	sterol regulatory element binding transcription factor 1	1.28E-08	99.32635
HU-2	6196	ribosomal protein S6 kinase alpha 2	1.38E-08	99.17665
CLS	6197	ribosomal protein S6 kinase, 90kDa, polypeptide 3	1.44E-08	98.87725
FHL3	2275	LIM-only protein FHL3	1.44E-08	98.87725
HU-1	6195	dJ590P13.1 (ribosomal protein S6 kinase, 90kD)	1.44E-08	98.87725
MYC	4609	myc proto-oncogene protein	1.49E-08	98.57784
SGK	6446	serum/glucocorticoid regulated kinase 1	1.89E-08	98.42814
DKFZp686N	2099	estrogen receptor 1	4.08E-08	98.27844
AP-1	3725	v-jun avian sarcoma virus 17 oncogene homolog	4.89E-08	98.12874
IMB1	3837	importin beta-1 subunit	6.07E-08	97.97904
AGMX1	695	dominant-negative kinase-deficient Brutons	6.92E-08	97.82934
DPC4	4089	mothers against decapentaplegic homolog 4	1.22E-07	97.67964
1-May	5580	protein kinase C, delta	1.26E-07	97.52994
ACT	71	actin-like protein	1.35E-07	97.30539
ACTB	60	PS1TP5-binding protein 1	1.35E-07	97.30539
DKFZp686C	4790	nuclear factor kappa-B, subunit 1	1.8E-07	97.08084
HSPG	6383	heparan sulfate proteoglycan core protein	2.46E-07	96.93114
GFRP1	3164	hormone receptor	2.67E-07	96.78144
APRF	6774	DNA-binding protein APRF	2.83E-07	96.63174
GSK3B	2932	glycogen synthase kinase 3 beta	3.13E-07	96.48204
AMCBX1	8517	incontinentia pigmenti	3.21E-07	96.33234
CSEN	30818	calsenilin, presenilin-binding protein	3.35E-07	96.18263
FLJ23903	4641	nuclear myosin I	4.21E-07	96.03293
MGC13177	5970	v-rel avian reticuloendotheliosis viral oncogene	4.32E-07	95.88323
AMPK	5563	AMP-activated protein kinase alpha 2 catalytic subunit	5.12E-07	95.65868
AMPK	5562	AMPK alpha 1	5.12E-07	95.65868
HSF1	3297	heat shock transcription factor 1	5.17E-07	95.43413
ASH	2885	growth factor receptor-bound protein 3	8.13E-07	95.28443
CRTC1	23373	transducer of regulated cAMP	9.57E-07	95.13473
DKFZp686H	3065	histone deacetylase 1	1.12E-06	94.98503
FLJ20408	3280	transcription factor HES-1	1.24E-06	94.83533
Arc-1	999	cell-CAM 120/80	1.28E-06	94.68563
CF2R	2149	thrombin receptor	1.32E-06	94.53593
DKFZp586N	4088	mad homolog JV15-2	1.52E-06	94.38623
ARB1	408	arrestin beta 1	1.56E-06	94.23653
HGS	9146	hepatocyte growth factor-regulated tyrosine	1.64E-06	94.08683
G-ALPHA-q	2776	guanine nucleotide binding protein (G protein)	1.91E-06	93.93713
PC	5624	protein C	1.97E-06	93.78743
JNK	5599	stress-activated protein kinase JNK1	2.05E-06	93.63772
MAPKAPK2	9261	mitogen-activated protein	2.13E-06	93.48802
CDX-3	1045	caudal type homeo box transcription factor 2	2.27E-06	93.33832

KIAA1047	9611	nuclear receptor co-repressor 1	2.29E-06	93.18862
IKBA	4792	nuclear factor of kappa light polypeptide gene enhancer in B-(	2.36E-06	93.03892
AFBP	3484	amniotic fluid binding protein	2.42E-06	92.88922
BRCA1	672	breast and ovarian cancer susceptibility protein 1	2.45E-06	92.73952
Jnkk2	5609	dual specificity mitogen-activated protein kinase	2.5E-06	92.58982
GNB2L1	10399	proliferation-inducing gene 21	2.86E-06	92.44012
MAP3K7	6885	transforming growth factor-beta-activated kinase 1	2.97E-06	92.29042
KRML	9935	transcription factor MAFB	3.23E-06	92.14072
DKFZp434K	7442	transient receptor potential vanilloid 1b	3.44E-06	91.99102
KIAA0988	6904	tubulin-specific chaperone d	3.5E-06	91.84132
AIS	8626	tumor protein p63	3.6E-06	91.69162
ACH	2261	fibroblast growth factor receptor 3	3.8E-06	91.54192
EAP1	9232	ESP1-associated protein 1	4.04E-06	91.39222
BID	637	BID isoform Si6	4.07E-06	91.24251
BFGF	2247	prostatropin	4.15E-06	91.09281
NFE2L2	4780	nuclear factor (erythroid-derived 2)-like 2	4.31E-06	90.94311
MGC15877	5499	serine/threonine protein phosphatase PP1-alpha 1	4.41E-06	90.64371
PPP1CC	5501	protein phosphatase 1, catalytic subunit	4.41E-06	90.64371
MGC3672	5500	protein phosphatase 1, catalytic subunit	4.41E-06	90.64371
DKFZp781I	4914	neurotrophic tyrosine kinase, receptor, type 1	4.52E-06	90.34431
DKFZp686I(	5327	alteplase	4.65E-06	90.19461
DELTA	7528	YY1 transcription factor	5.31E-06	90.04491
CK2B	1460	casein kinase 2, beta polypeptide	5.36E-06	89.89521
C2TA	4261	class II transactivator	5.39E-06	89.74551
DTR	1839	heparin-binding epidermal growth factor	5.44E-06	89.59581
AIF4	83737	itchy homolog E3 ubiquitin protein ligase	5.54E-06	89.44611
CDC2	983	cell cycle controller CDC2	5.64E-06	89.29641
IRF-1	3659	interferon regulatory factor-1	5.79E-06	89.14671
ARP1	7026	transcription factor COUP 2	7.08E-06	88.99701
BEK	2263	FGF receptor	7.18E-06	88.84731
SNAPAP	23557	SNARE associated protein snapin	7.46E-06	88.6976
BCL2	596	B-cell CLL/lymphoma 2	7.75E-06	88.5479
HEB	6938	transcription factor 12	8.04E-06	88.3982
MKNK1	8569	MAP kinase interacting serine/threonine kinase 1	8.1E-06	88.2485
GCCR	2908	glucocorticoid receptor	8.22E-06	88.0988
CAMK2A	815	CaM kinase II alpha subunit	8.97E-06	87.9491
CSBP1	1432	stress-activated protein kinase 2A	9.12E-06	87.7994
NR1I1	7421	vitamin D (1,25-dihydroxyvitamin D3) receptor	9.19E-06	87.6497
ACLS	2737	GLI-Kruppel family member GLI3	9.82E-06	87.5
D12S1644	6778	signal transducer and activator of transcription 6	9.95E-06	87.3503
HSP75	10131	heat shock protein 75	1E-05	86.97605
D6S182	3326	heat shock 90kDa protein 1, beta	1E-05	86.97605
ECGP	7184	heat shock protein 90kDa beta, member 1	1E-05	86.97605
FLJ31884	3320	heat shock protein 90kDa alpha (cytosolic)	1E-05	86.97605
MYF4	4656	myogenin	1.08E-05	86.6018
IB1	9479	mitogen-activated protein kinase 8	1.12E-05	86.4521
PIK3CD	5293	phosphatidylinositol 3-kinase	1.22E-05	86.15269

DKFZp779K	5291	PI3-kinase p110 subunit beta	1.22E-05	86.15269
MGC14216	5290	phosphatidylinositol 3-kinase, catalytic	1.22E-05	86.15269
C-Rel	5966	v-rel reticuloendotheliosis viral oncogene homolog	1.28E-05	85.85329
HOX1	3202	homeobox A5	1.31E-05	85.70359
D2DR	1813	seven transmembrane helix receptor	1.32E-05	85.55389
AAG11	2274	aging-associated gene 11	1.32E-05	85.40419
A2MR	4035	type V tgf-beta receptor	1.36E-05	85.25449
AP-2	7020	transcription factor AP-2 alpha	1.45E-05	85.10479
BCL10	8915	CARD-like apoptotic protein	1.69E-05	84.95509
BETA-TRCP	8945	beta-TrCP1	1.8E-05	84.80539
GCN5	2648	GCN5 general control of amino-acid synthesis 5-like 2	1.99E-05	84.65569
KOR	4986	opioid receptor, kappa 1	2.23E-05	84.50599
AWD	4830	NDP kinase A	2.35E-05	84.35629
CAMK4	814	CAM kinase IV	2.42E-05	84.20659
CEBPZ	1649	growth arrest- and DNA damage-inducible	2.44E-05	84.05689
BAPX1	579	bagpipe homeobox 1	2.69E-05	83.90719
MGC:3310	7189	TNF receptor-associated factor 6	2.73E-05	83.75749
EDNRA	1909	endothelin receptor type A	2.79E-05	83.60778
MGC11154	5879	migration-inducing gene 5	2.83E-05	83.45808
C/EBP-beta	1051	transcription factor 5	2.87E-05	83.30838
PRKAR2	5577	protein kinase, cAMP-dependent	2.89E-05	83.15868
ALPS2B	841	cysteine protease	2.9E-05	83.00898
JAK-3	3718	Janus kinase 3	2.99E-05	82.85928
DAP-150	1639	p150-glued	3.1E-05	82.63473
DDPAC	4137	microtubule-associated protein tau, isoform 4	3.1E-05	82.63473
HIF-1alpha	3091	member of PAS superfamily 1	3.53E-05	82.41018
BSAP	5079	B-cell lineage specific activator	3.64E-05	82.26048
CIG	2335	fibronectin 1	3.82E-05	82.11078
NR1C3	5468	PPAR gamma	3.85E-05	81.96108
HIRS-1	3667	insulin receptor substrate 1	3.87E-05	81.81138
CP107	5933	retinoblastoma-like 1 (p107)	3.89E-05	81.66168
DKFZp686E	6772	signal transducer and activator of transcription-1	3.9E-05	81.51198
GNA-11	2767	guanine nucleotide-binding protein, Gq class, GNA11	3.97E-05	81.36228
IB2	23542	mitogen-activated protein kinase 8	4.04E-05	81.21257
INrf2	9817	kelch-like ECH-associated protein 1	4.06E-05	81.06287
MGC10283	5566	protein kinase A catalytic subunit	4.13E-05	80.91317
C/EBP-alph	1050	CCAAT/enhancer binding protein alpha	4.21E-05	80.76347
ABP-280	2316	filamin A, alpha	4.21E-05	80.61377
BLIMP1	639	beta-interferon gene positive-regulatory domain I	4.26E-05	80.46407
FKH1	2308	forkhead homolog in rhabdomyosarcoma	4.28E-05	80.31437
CAK1	1022	serine/threonine protein kinase MO15	4.31E-05	80.16467
ACUG	64127	caspase recruitment domain protein 15	4.39E-05	80.01497
CSK	1445	c-src tyrosine kinase	4.49E-05	79.86527
MAPK13	5603	mitogen-activated protein kinase p38 delta	4.54E-05	79.71557
CD217	23765	interleukin 17 receptor A	4.55E-05	79.49102
FLJ95963	84818	interleukin 17 receptor-like	4.55E-05	79.49102
G13	10672	guanine nucleotide binding protein (G protein)	4.73E-05	79.19162

GNA12	2768	guanine nucleotide binding protein (G protein)	4.73E-05	79.19162
CAM2	816	proline rich calmodulin-dependent protein kinase	5.03E-05	78.81737
CAMK	818	calcium/calmodulin-dependent protein kinase	5.03E-05	78.81737
CAMK2D	817	calcium/calmodulin-dependent protein kinase II delta	5.03E-05	78.81737
SFN	2810	stratifin	5.06E-05	78.51796
GNB1	2782	transducin beta chain 1	5.46E-05	78.21856
GNG1	2792	guanine nucleotide binding protein (G protein)	5.46E-05	78.21856
ADRBK1	156	beta adrenergic receptor kinase 1	5.46E-05	78.21856
CD309	3791	vascular endothelial growth factor receptor 2	5.62E-05	77.91916
C2orf31	7855	seven-transmembrane receptor frizzled-5	5.7E-05	77.76946
AIM	1786	DNA (cytosine-5-)-methyltransferase 1	5.71E-05	77.61976
DKFZp781H	9451	eukaryotic translation initiation factor 2-alpha kinase 3	5.72E-05	77.47006
CD44	960	chondroitin sulfate proteoglycan 8	5.95E-05	77.32036
PI5	5268	serpin peptidase inhibitor, clade B (ovalbumin)	5.99E-05	77.17066
AHR	196	aromatic hydrocarbon receptor	6.01E-05	77.02096
PPP1R7	5510	protein phosphatase-1 regulatory subunit 7 beta1	6.05E-05	76.87126
FYN	2534	src-like kinase	6.06E-05	76.72156
CAP20	1026	CDK-interaction protein 1	6.17E-05	76.57186
CK2A1	1457	casein kinase II alpha subunit	7.01E-05	76.34731
CK2A2	1459	casein kinase 2, alpha prime polypeptide	7.01E-05	76.34731
DKFZp781F	5325	PLAG-like 1	7.08E-05	76.12275
CANP	823	calpain 1, large subunit	7.14E-05	75.97305
GRIN2B	2904	glutamate receptor subunit epsilon-2	7.28E-05	75.82335
POMP100	6421	polypyrimidine tract-binding protein-associated	7.34E-05	75.67365
ARH12	387	oncogene RHO H12	7.45E-05	75.52395
IRF4	3662	interferon regulatory factor 4	7.47E-05	75.2994
DAPK3	1613	ZIP kinase isoform	7.47E-05	75.2994
EDF	3624	inhibin, beta A (activin A, activin AB alpha polypeptide)	7.75E-05	74.92515
BMP-11	10220	growth differentiation factor 11	7.75E-05	74.92515
ACTRIIB	93	activin A type IIB receptor	7.75E-05	74.92515
CAR	846	extracellular calcium-sensing receptor	7.83E-05	74.4012
RAMP1	10267	calcitonin receptor-like receptor activity	7.83E-05	74.4012
RAMP3	10268	receptor activity modifying protein 3	7.83E-05	74.4012
RAMP2	10266	receptor (calcitonin) activity modifying protein 2	7.83E-05	74.4012
CBX4	8535	chromobox homolog 4	7.92E-05	74.02695
FLJ43106	7249	tuberin	7.93E-05	73.87725
C20orf97	57761	p65-interacting inhibitor of NF-kappaB	8.17E-05	73.72754
CD36	948	scavenger receptor class B, member 3	8.39E-05	73.57784
MGC13185	7334	ubiquitin carrier protein N	8.42E-05	73.42814
ESA1	10524	cPLA2 interacting protein	8.62E-05	73.27844
ASK1	4217	MAPK/ERK kinase kinase 5	8.69E-05	73.12874
CALR	811	Sicca syndrome antigen A (autoantigen Ro; calreticulin)	8.78E-05	72.97904
BAF190	6595	SWI/SNF-related matrix-associated actin-dependent	8.79E-05	72.82934
COLEC1	4153	mannose-binding protein C	9.44E-05	72.67964
HD3	8841	histone deacetylase 3	9.6E-05	72.52994
PLK	5347	polo like kinase	9.63E-05	72.38024
PRO1280	51684	suppressor of fused homolog (Drosophila)	9.71E-05	72.23054

CD124	3566	interleukin-4 receptor alpha chain	0.000103	72.00599
CD132	3561	common cytokine receptor gamma chain	0.000103	72.00599
CD56	4684	neural cell adhesion molecule, NCAM	0.000103	71.78144
BCL3	602	B-cell leukemia/lymphoma 3	0.000103	71.63174
DLG4	1742	synapse-associated protein 90	0.000103	71.48204
APOER2	7804	apolipoprotein E receptor 2	0.000104	71.33234
ARMD10	7099	homolog of Drosophila toll	0.000104	71.18263
ELK4	2005	SRF accessory protein 1	0.000106	71.03293
CRM1	7514	exportin 1 (CRM1, yeast, homolog)	0.000108	70.88323
BSF3	23529	B-cell stimulating factor 3	0.00011	70.73353
DARPP-32	84152	protein phosphatase 1, regulatory (inhibitor)	0.000111	70.58383
EDN2	1907	endothelin 2	0.000114	70.35928
EDN3	1908	truncated endothelin 3	0.000114	70.35928
AT1	472	serine-protein kinase ATM	0.000116	70.13473
COUP-TFI	7025	transcription factor COUP 1	0.00012	69.98503
CRSP1	5469	vitamin D receptor-interacting protein complex	0.000121	69.83533
BSF2	3569	interleukin 6	0.000121	69.68563
53BP1	7158	tumor protein 53-binding protein, 1	0.000122	69.53593
CHNG1	7253	thyroid stimulating hormone receptor	0.000124	69.38623
C1Q-C	714	complement component 1, q subcomponent, C chain	0.000132	69.08683
C1QB	713	complement component 1, q subcomponent	0.000132	69.08683
C1QA	712	complement component C1q, A chain	0.000132	69.08683
ATOD4	9021	suppressor of cytokine signaling 3	0.000133	68.78743
FLJ25220	3679	integrin alpha 7	0.000134	68.56287
CD29	3688	integrin beta 1	0.000134	68.56287
FLJ26504	6464	SHC (Src homology 2 domain containing)	0.000138	68.33832
ADORA2	136	adenosine A2b receptor	0.00014	68.18862
CD49e	3678	integrin, alpha 5 (fibronectin receptor)	0.000156	68.03892
TREB5	7494	X-box binding protein 1	0.000157	67.88922
DIAPH3	81624	diaphanous homolog 3	0.00016	67.73952
TSG10	7251	tumor susceptibility gene 101	0.000163	67.58982
FOG	161882	friend of GATA-1	0.000164	67.44012
NRSF	5978	repressor binding to the X2 box	0.000164	67.29042
C2orf5	150684	MURR1	0.00018	67.14072
MIZ-1	7709	zinc finger protein 151 (pHZ-67)	0.000183	66.99102
GIP	2771	guanine nucleotide binding protein (G protein)	0.000184	66.84132
BTF2	2965	general transcription factor IIH, polypeptide 1, 62kDa	0.000184	66.69162
FLJ36302	7074	T-cell lymphoma invasion and metastasis 1	0.000187	66.54192
MGC8367	5111	proliferating cell nuclear antigen	0.000187	66.39222
F-SRC-1	8648	nuclear receptor coactivator 1	0.000193	66.24251
GPRC1A	2911	glutamate receptor, metabotropic 1	0.000199	66.09281
CLG4B	4318	gelatinase B	0.0002	65.94311
BCAR1	9564	Cas scaffolding protein family member 1	0.000201	65.79341
DKFZp686C	5148	rod cG-PDE G	0.000206	65.56886
CSNBAD3	2779	transducin alpha-1 chain	0.000206	65.56886
ERN1	2081	endoplasmic reticulum to nucleus signaling 1	0.000206	65.34431
CCR1	1230	chemokine (C-C motif) receptor 1	0.000212	65.19461

DELTA1	28514	delta-like 1	0.000212	65.04491
GIG8	3398	cell growth-inhibiting gene 8	0.000213	64.89521
OPRD	4985	opioid receptor, delta 1	0.00022	64.74551
CRTC2	200186	transducer of regulated cAMP response	0.000225	64.59581
BCL5	604	zinc finger protein 51	0.000228	64.44611
AOF2	23028	lysine (K)-specific demethylase 1	0.00023	64.29641
CAMK1	8536	calcium/calmodulin-dependent protein kinase I	0.000233	64.14671
IBP5	3488	insulin-like growth factor binding protein 5	0.000235	63.99701
CD130	3572	CD130 antigen	0.000238	63.84731
ACVR1C	130399	activin receptor-like kinase 7	0.00024	63.6976
ARR3	407	cone arrestin	0.000244	63.5479
ARHGAP1	392	Rho GTPase activating protein 1	0.000251	63.3982
CC-CKR-2	1231	monocyte chemoattractant protein 1 receptor	0.000251	63.2485
HDLCQ11	4023	lipoprotein lipase	0.000253	63.0988
CDHF12	5979	ret proto-oncogene (multiple endocrine neoplasia)	0.000254	62.9491
CCL3	6348	small inducible cytokine A3	0.000256	62.72455
CC-CKR-5	1234	chemokine receptor CCR5	0.000256	62.72455
HsT17432	4091	MAD, mothers against decapentaplegic homolog 6	0.000265	62.5
CEBP	64506	cytoplasmic polyadenylation element binding protein 1	0.000268	62.3503
PP2CB	5516	serine/threonine protein phosphatase 2A	0.000269	62.12575
PP2Ac	5515	protein phosphatase 2, catalytic subunit, alpha isoform	0.000269	62.12575
FLJ10671	8295	350/400 kDa PCAF-associated factor	0.000271	61.9012
AXIN	8312	axin 1	0.000273	61.67665
AXIL	8313	axin 2	0.000273	61.67665
CCL2	6347	small inducible cytokine A2	0.000282	61.4521
MAD7	6945	transcription factor-like 4	0.000283	61.3024
AKT2	208	v-akt murine thymoma viral oncogene homolog 2	0.000285	61.15269
GIG34	6135	60S ribosomal protein L11	0.000289	61.00299
G0S24	7538	zinc finger protein, C3H type, 36 homolog (mouse)	0.000289	60.85329
BXR	8856	pregnane X receptor	0.000294	60.70359
BNSP	6696	SPP1/CALPHA1 fusion	0.000304	60.55389
AAA	351	amyloid beta A4 protein	0.000308	60.40419
C4BP	722	C4b binding protein, alpha chain	0.000317	60.17964
C4BP	725	C4b binding protein, beta chain	0.000317	60.17964
CHC	1213	clathrin, heavy chain (Hc)	0.000319	59.88024
INPP5F	4952	oculocerebrorenal syndrome of Lowe	0.000319	59.88024
CCS-3	1915	translation elongation factor 1 alpha 1-like 14	0.000322	59.65569
CD126	3570	interleukin 6 receptor alpha subunit	0.000356	59.50599
CD221	3480	insulin-like growth factor 1 receptor	0.000365	59.35629
CD11B	3684	integrin alpha M	0.000368	59.13174
CD18	3689	complement receptor C3 beta-subunit	0.000368	59.13174
EMC19	6500	transcription elongation factor B (SIII), polypeptide 1	0.00038	58.90719
CD136	4486	macrophage stimulating 1 receptor	0.00038	58.75749
C7orf16	10842	G-substrate	0.000388	58.60778
ABD-B	3205	homeo box A9	0.00039	58.45808
FBP	8880	far upstream element-binding protein	0.000397	58.23353
CRD-BP	10642	insulin-like growth factor 2 mRNA binding protein 1	0.000397	58.23353

FJHN	6928	transcription factor 2, hepatic	0.000402	58.00898
ATF3	467	activating transcription factor 3	0.000412	57.85928
GDF8	2660	myostatin	0.00043	57.70958
CCK-B	887	CCK2 receptor	0.000434	57.55988
GRB1	5295	phosphoinositide-3-kinase, regulatory subunit	0.000455	57.26048
P85B	5296	phosphoinositide-3-kinase, regulatory subunit 2	0.000455	57.26048
DKFZp686F	8503	phosphoinositide-3-kinase, regulatory subunit	0.000455	57.26048
ACT2	6351	lymphocyte-activation gene 1	0.000459	56.96108
IPOA1	3838	karyopherin alpha 2	0.00046	56.81138
MGC12610	4314	matrix metalloproteinase 3	0.00047	56.66168
CLTA	1211	clathrin, light chain (Lca)	0.000474	56.43713
CLTB	1212	clathrin, light polypeptide	0.000474	56.43713
ABL	25	proto-oncogene tyrosine-protein kinase ABL1	0.000479	56.21257
ASC	29108	apoptosis-associated speck-like protein	0.000479	56.06287
CD55	1604	decay accelerating factor for complement	0.000496	55.91317
Hs.89862	8717	tumor necrosis factor receptor type 1 associated death	0.00051	55.76347
DKFZp686J	57496	myocardin-related transcription factor B	0.000512	55.61377
HNRPE1	5093	poly(rC)-binding protein 1	0.000526	55.46407
MGC21659	7069	spot 14 protein	0.000546	55.31437
DJ-1	11315	protein DJ-1	0.000555	55.08982
PI4K-BETA	5298	type III phosphatidylinositol 4-kinase beta	0.000555	55.08982
CABP2	51475	calcium binding protein 2	0.00056	54.86527
ENO1	2023	phosphopyruvate hydratase	0.000567	54.71557
KIAA1303	57521	regulatory associated protein of mTOR	0.000568	54.56587
CCL7	6354	chemokine (C-C motif) ligand 7	0.000571	54.41617
FLJ44809	2475	FKBP12-rapamycin complex-associated protein 1	0.000593	54.26647
FLT4	2324	fms-related tyrosine kinase 4	0.000598	54.11677
MYD88	4615	myeloid differentiation primary response gene 88	0.000608	53.96707
CNTFR	1271	CNTFR alpha	0.00062	53.74251
CD118	3977	CD118 antigen	0.00062	53.74251
ACTRIB	91	serine(threonine) protein kinase receptor R2	0.00063	53.51796
MGC41878	5579	protein kinase C, beta 1	0.000655	53.36826
DKFZp686K	10625	NS1-binding protein	0.00066	53.14371
AIP	9049	aryl hydrocarbon receptor interacting protein	0.00066	53.14371
DKFZp762F	55662	hypoxia-inducible factor 1, alpha subunit inhibitor	0.000665	52.91916
SREBF2	6721	sterol regulatory element-binding protein 2	0.000665	52.76946
103AS, 15.:	3805	NK cell receptor	0.000677	52.61976
MGC13823	4838	nodal, mouse, homolog	0.000687	52.47006
IKBKE	9641	inhibitor of kappa light polypeptide gene enhancer	0.00069	52.32036
NEC1	5122	proprotein convertase subtilisin/kexin type 1	0.000698	52.17066
NGR	65078	Nogo-66 receptor	0.0007	52.02096
EEK	2046	EPH- and ELK-related tyrosine kinase	0.000705	51.87126
CCL11	6356	small inducible cytokine A11	0.000714	51.72156
HOX-2.6	3214	homeobox B4	0.000724	51.57186
ACVRIP1	9863	membrane associated guanylate kinase, WW and PDZ	0.000727	51.42216
PFN1	5216	profilin 1	0.000735	51.1976
D3S1319E	5217	profilin 2	0.000735	51.1976

FLJ17670	7535	zeta-chain associated protein kinase 70kDa	0.000779	50.97305
MGC70609	7422	vascular permeability factor	0.000788	50.82335
MGMT	4255	O-6-methylguanine-DNA methyltransferase	0.00079	50.67365
CK1	1452	down-regulated in lung cancer	0.000794	50.52395
EGR3	1960	zinc finger protein pilot	0.00082	50.37425
NOR-90	7343	upstream binding transcription factor, RNA polymerase I	0.00082	50.22455
CLOCK	9575	clock homolog (mouse)	0.00083	50.07485
CSBP	3190	transformation upregulated nuclear protein	0.000854	49.92515
D16S432E	51741	WW domain-containing protein WWOX	0.000864	49.77545
LYT-10	4791	nuclear factor of kappa light chain gene enhancer	0.000867	49.62575
CCNA1	8900	cyclin A1	0.000868	49.47605
ARB2	409	arrestin, beta 2	0.000869	49.32635
FLJ43224	4738	neural precursor cell expressed, developmentally	0.000871	49.17665
RRAS	6237	Oncogene RRAS	0.000879	49.02695
MGC12689	5020	oxytocin-neurophysin I, preproprotein	0.000879	48.8024
OT-R	5021	oxytocin receptor	0.000879	48.8024
MGC11118	5645	trypsin 2	0.000898	48.42814
MTG	5646	trypsin 3	0.000898	48.42814
MGC12017	5644	nonfunctional trypsin 1	0.000898	48.42814
ARF	1029	multiple tumor suppressor 1	0.000919	48.12874
CDC25	5923	Ras-specific nucleotide exchange factor CDC25	0.000923	47.97904
MAD2	4601	Max-related transcription factor	0.000933	47.82934
PLCG2	5336	phospholipase C gamma 2	0.000968	47.60479
APKD2	5311	polycystic kidney disease 2 (autosomal dominant)	0.000968	47.60479
TCF-3	83439	transcription factor 7-like 1 (T-cell specific, HMG-box)	0.001037	47.38024
GEFT	115557	RAC/CDC42 exchange factor	0.001041	47.23054
ACTA	58	alpha skeletal muscle actin	0.001046	46.85629
ACT	72	alpha-actin 3	0.001046	46.85629
ACTC	70	cardiac muscle alpha actin 1	0.001046	46.85629
AAT6	59	actin, alpha 2, smooth muscle, aorta	0.001046	46.85629
FLJ26075	5605	mitogen-activated protein kinase kinase 2, p45	0.001054	46.48204
DKFZp762N	23162	homolog of Drosophila Sunday driver 2	0.001061	46.33234
CCNE2	9134	cyclin E2	0.001072	46.18263
FLJ12099	5602	mitogen-activated protein kinase 10	0.001078	46.03293
ABI-1	10006	nap1 binding protein	0.001104	45.88323
FLJ36364	6932	transcription factor 7 (T-cell specific, HMG-box)	0.001109	45.58383
TCF-4	6934	transcription factor 7-like 2 (T-cell specific, HMG-box)	0.001109	45.58383
DKFZp586H	51176	lymphoid enhancer binding factor-1	0.001109	45.58383
BA554C12.	9978	ZYP protein	0.001109	45.05988
ELOB	6923	transcription elongation factor B (SIII), polypeptide 2	0.001109	45.05988
SIII	6921	transcription elongation factor B, polypeptide 1	0.001109	45.05988
CUL2	8453	cullin 2	0.001109	45.05988
CD344	8322	WNT receptor frizzled-4	0.001123	44.68563
FLH21957	9448	hepatocyte progenitor kinase-like/germinal center	0.001127	44.53593
C23	4691	nucleolin	0.001132	44.38623
DLL4	54567	notch ligand DLL4	0.001132	44.23653
ADH	551	arginine vasopressin-neurophysin II	0.001148	44.08683

DKFZp686(	79048	selenocysteine insertion sequence binding protein 2	0.001161	43.78743
MGC14353	84817	thioredoxin domain containing 17	0.001161	43.78743
GRIM-12	7296	thioredoxin reductase 1	0.001161	43.78743
MGC10480	5757	prothymosin alpha protein	0.001179	43.48802
ARHGAP14	9901	WAVE-associated Rac GTPase activating protein	0.001188	43.33832
FLJ14314	57551	serine/threonine kinase TAO1	0.001217	43.11377
EMK1	2011	ELKL motif kinase 1	0.001217	43.11377
TGFBRAP1	9392	TGF beta receptor associated protein -1	0.001222	42.88922
FLJ31482	8936	verprolin homology domain-containing protein 1	0.001253	42.73952
FLJ97193	10023	proto-oncogene FRAT1	0.001266	42.58982
CDK4I	1030	cyclin-dependent kinases 4 and 6 binding protein	0.001267	42.44012
MTA1	9112	metastasis associated protein	0.001285	42.21557
MSP23	5052	peroxiredoxin 1	0.001285	42.21557
CD295	3953	leptin receptor	0.001293	41.99102
CALL	10752	cell adhesion molecule with homology to L1CAM	0.001325	41.84132
ACAP1	9744	centaurin beta1	0.001332	41.69162
HOX2	3211	homeobox B1	0.001336	41.54192
C6	5688	proteasome subunit alpha 4	0.00134	41.39222
APLP	333	amyloid-like protein 1	0.00136	41.24251
FLJ25596	6655	son of sevenless homolog 2	0.001365	41.01796
GF1	6654	son of sevenless homolog 1	0.001365	41.01796
IRS2	8660	insulin receptor substrate 2	0.001373	40.79341
FX	7114	thymosin beta 4, X-linked	0.001381	40.56886
COCA2	4292	MutL protein homolog 1	0.001381	40.56886
CDK8	1024	CDK8 protein kinase	0.0014	40.34431
CD49f	3655	integrin alpha6B	0.001429	40.11976
CD104	3691	GP150	0.001429	40.11976
APC	324	adenomatosis polyposis coli	0.001445	39.89521
DKFZp547(	25970	SH2B adaptor protein 1	0.001462	39.74551
C-FMS	1436	macrophage colony stimulating factor I receptor	0.001468	39.59581
CCBP2	1238	chemokine (C-C motif) receptor 9	0.001496	39.44611
CASP8AP2	9994	human FLASH	0.001506	39.29641
ANF	8820	homeobox, ES cell expressed 1	0.001513	39.14671
DKFZp686A	3146	high-mobility group (nonhistone chromosomal)	0.001516	38.92216
HMG2	3148	high-mobility group box 2	0.001516	38.92216
DKFZp779A	4134	microtubule-associated protein 4	0.001521	38.6976
GNG11	2791	guanine nucleotide-binding protein G(I)/G(S)/G(O)	0.001551	38.3982
KIAA1415	57580	phosphatidylinositol-3,4,5-trisphosphate-dependent	0.001551	38.3982
G(gamma)1	51764	guanine nucleotide binding protein (G protein)	0.001551	38.3982
CACNB3	784	calcium channel, voltage-dependent, beta 3 subunit	0.001562	38.0988
CKBBP2	90480	papillomavirus L2 interacting nuclear protein 1	0.001564	37.9491
GLUH1	2890	glutamate receptor, ionotropic, AMPA 1	0.001579	37.72455
DKFZp781H	5924	Ras protein-specific guanine nucleotide-releasing	0.001579	37.72455
VAV2	7410	Protein vav-2	0.001583	37.5
ARH	26119	LDL receptor adaptor protein	0.001621	37.3503
ANCR	7337	ubiquitin protein ligase E3A	0.001666	37.2006
G-ALPHA-h	7052	TGase-H	0.001741	37.0509

APBA1	320	amyloid beta A4 precursor protein-binding, family A	0.001755	36.9012
FKHL8	2297	forkhead box D1	0.001817	36.7515
ARNO	9266	cytohesin 2	0.001826	36.6018
ARF6	382	ADP-ribosylation factor 6	0.001883	36.37725
ARFGEP10(	9922	IQ motif and Sec7 domain 1	0.001883	36.37725
FLJ12859	23528	ZNP-99 transcription factor	0.001884	36.15269
DOCK1	1793	dedicator of cyto-kinesis 1	0.001909	36.00299
GPCR	117196	MAS-related GPR, member X4	0.00191	35.77844
GPCR	259249	MAS-related GPR, member X1	0.00191	35.77844
CNTF	1270	ciliary neurotrophic factor	0.001925	35.55389
DKFZp586D	9477	Trf (TATA binding protein-related factor)-proximal	0.00194	35.40419
VASP	7408	vasodilator-stimulated phosphoprotein	0.00194	35.25449
MEF2C	4208	MADS box transcription enhancer factor 2, polypeptide C (my	0.001953	34.88024
DKFZp686I	4209	myocyte enhancer factor 2D	0.001953	34.88024
FLJ32599	4207	myocyte enhancer factor 2B	0.001953	34.88024
ADCAD1	4205	myocyte enhancer factor 2A	0.001953	34.88024
DOCK2	1794	dedicator of cytokinesis 2	0.001958	34.50599
PAR-6, PAR	50855	partitioning-defective protein 6	0.001969	34.35629
CSNK1E	1454	casein kinase 1 epsilon	0.002066	34.20659
KLK7	5650	stratum corneum chymotryptic enzyme	0.002086	34.05689
DKFZp686C	171024	genethonin 2	0.002157	33.90719
AAD10	8085	trinucleotide repeat containing 21	0.002197	33.75749
ARHGEF14	23263	MCF2 transforming sequence-like protein	0.002222	33.60778
IGIF	3606	interleukin-18	0.00224	33.38323
CD218a	8809	IL1 receptor-related protein	0.00224	33.38323
RPS6	6194	phosphoprotein NP33	0.002263	33.15868
AGER	177	advanced glycosylation end product-specific receptor	0.002315	33.00898
DKFZp566J	9039	UBA3, ubiquitin-activating enzyme E1 homolog	0.002362	32.78443
A-116A10.1	8883	NEDD8-activating enzyme E1 subunit	0.002362	32.78443
CHEDG1	1901	sphingosine 1-phosphate receptor EDG1	0.002365	32.55988
CHN	8013	chondrosarcoma, extraskeletal myxoid, fused to EWS	0.002432	32.26048
H1	6013	prorelaxin	0.002432	32.26048
1200006M	55827	androgen receptor complex-associated protein	0.002432	32.26048
OS9	10956	amplified in osteosarcoma	0.002472	31.96108
DKFZp781F	9788	missing in metastasis	0.002498	31.81138
KIAA0339	9739	SET domain containing 1A	0.002518	31.66168
CARD12	58484	caspase recruitment domain family, member 12	0.002534	31.51198
BAF155	6599	chromatin remodeling complex BAF155 subunit	0.002547	31.36228
NK1R	6869	NK-1 receptor	0.002575	31.21257
AMPH2	274	bridging integrator 1	0.002587	31.06287
GABATHG	6529	solute carrier family 6	0.002591	30.76347
HPC-1	6804	syntaxin 1A (brain)	0.002591	30.76347
FLJ12615	64398	MAGUK p55 subfamily member 5	0.002591	30.76347
ARNO3	9265	general receptor of phosphoinositides 1	0.002622	30.46407
CHIP	10273	serologically defined colon cancer antigen 7	0.00271	30.23952
ARA70	8031	nuclear receptor coactivator 4	0.00271	30.23952
ASCL1	429	achaete-scute homolog 1	0.002862	30.01497

KIAA0595	23082	peroxisome proliferator-activated receptor gamma	0.002874	29.86527
APOA1	335	apolipoprotein A1	0.002973	29.71557
CALCOCO1	57658	coiled-coil transcriptional coactivator	0.003009	29.41617
CUL4B	8450	cullin-4B	0.003009	29.41617
HZF-3	4929	transcriptionally inducible nuclear receptor related 1	0.003009	29.41617
FLOT1	10211	flotillin 1	0.003087	29.11677
FLJ00280	6256	retinoid X receptor, alpha	0.00312	28.81737
MGC12657	8828	neuropilin-2a(22)	0.00312	28.81737
HAP	5915	retinoic acid receptor, beta polypeptide	0.00312	28.81737
B2-1	9267	homolog of secretory protein SEC7	0.003205	28.51796
LIMK	3984	LIM domain kinase 1	0.003333	28.21856
C4orf1	10463	chromosome 4 open reading frame 1	0.003333	28.21856
AIRE	326	autoimmune regulator	0.003333	28.21856
BITH	83658	cytoplasmic dynein light chain 2A	0.003395	27.91916
Flt4-L	7424	vascular endothelial growth factor-related protein	0.003403	27.76946
APCA	773	brain calcium channel 1	0.003429	27.61976
ARHG	391	ras homolog gene family, member G (rho G)	0.00346	27.39521
DOCK4	9732	dedicator of cytokinesis 4	0.00346	27.39521
MSK2	8986	mitogen- and stress-activated protein kinase 2	0.003464	27.09581
MGC1911	9252	mitogen- and stress-activated protein kinase 1	0.003464	27.09581
FIGF	2277	vascular endothelial growth factor D	0.003485	26.87126
CAS	1434	CSE1 chromosome segregation 1-like protein	0.003486	26.72156
ACTN1	87	F-actin cross-linking protein	0.0035	26.57186
MSI1	4440	Musashi (Drosophila) homolog 1	0.003509	26.42216
AC068139.	6435	surfactant, pulmonary-associated protein A1B	0.003537	26.27246
CRARF	5648	manan-binding lectin serine protease-1	0.003632	26.12275
CACH2	775	voltage-gated calcium channel alpha subunit Cav1.2	0.003709	25.8982
CAB1	782	dihydropyridine-sensitive L-type, calcium channel	0.003709	25.8982
EPHA4	2043	tyrosine-protein kinase receptor SEK	0.003731	25.67365
PAX2	5076	paired box protein 2	0.003739	25.52395
HMOX2	3163	heme oxygenase (decycling) 2	0.00375	25.2994
CACNB2	783	calcium channel, voltage-dependent, beta 2 subunit	0.00375	25.2994
B56A	5525	serine/threonine protein phosphatase 2A, 56 kDa	0.003813	25.07485
DBS	4036	Heymann nephritis antigen homolog	0.003926	24.92515
KOR-3	4987	kappa3-related opioid receptor	0.00394	24.7006
GPR74	10886	neuropeptide FF 2	0.00394	24.7006
HOP	10963	stress-induced-phosphoprotein 1	0.004052	24.32635
FLJ14581	10527	RAN binding protein 7	0.004052	24.32635
H2	6019	prorelaxin H2	0.004052	24.32635
EPHA3	2042	ephrin receptor EphA3	0.004082	24.02695
CNR2	56142	KIAA0345-like 8	0.004159	23.87725
CNR1	56144	ortholog of mouse CNR1	0.004204	23.72754
DKFZp586I	8498	RAN-binding protein-3	0.004238	23.57784
AVPR1B	553	antidiuretic hormone receptor 1B	0.004254	23.42814
EAT2	117157	SH2 domain-containing molecule EAT2	0.004294	23.27844
BTEB	687	basic transcription element binding protein 1	0.004299	23.12874
C8FW	10221	G-protein-coupled receptor induced protein	0.004418	22.97904

ALXR	2358	lipoxin A4 receptor (formyl peptide receptor related)	0.004422	22.82934
GP6	51206	glycoprotein VI (platelet)	0.004458	22.67964
CD2AP	23607	CD2-associated protein	0.004622	22.52994
FLJ14040	84148	MYST histone acetyltransferase 1	0.004663	22.38024
DAZ	1617	deleted in azoospermia	0.004763	22.00599
DAZ2	57055	deleted in azoospermia 2	0.004763	22.00599
DAZ3	57054	deleted in azoospermia 3	0.004763	22.00599
DAZ	57135	deleted in azoospermia 4	0.004763	22.00599
SCAP1	8631	src kinase associated phosphoprotein 1	0.004809	21.63174
AHH	57491	aryl hydrocarbon receptor regulator	0.004877	21.48204
IFI-4	4938	2-5A synthetase 1	0.004962	21.33234
PTP-PEST	5782	protein-tyrosine phosphatase G1	0.005033	21.18263
DUET	8997	serine/threonine kinase with Dbl- and pleckstrin	0.005266	21.03293
CSCD	1634	small leucine-rich protein 1B	0.005296	20.88323
HTF9A	5902	RAN binding protein 1	0.005331	20.73353
APT1LG1	356	tumor necrosis factor (ligand) superfamily, member 6	0.005389	20.58383
FLJ20819	7879	Ras-associated protein RAB7	0.005432	20.43413
ATBF1	463	AT motif-binding factor 1	0.005459	20.28443
SSTR2	6752	somatostatin receptor 2	0.005624	20.13473
ACPA	5657	proteinase 3 (serine proteinase, neutrophil, Wegener)	0.005639	19.98503
HES5	388585	hairy and enhancer of split 5 (Drosophila)	0.005766	19.83533
MMP11	4320	matrix metalloproteinase 11	0.005795	19.68563
FMOD	2331	fibromodulin proteoglycan	0.005814	19.53593
EMT	3702	tyrosine-protein kinase ITK/TSK	0.005843	19.38623
FLJ23079	6616	synaptosomal-associated protein 25	0.005867	19.23653
TBXA2R	6915	prostanoid TP receptor	0.005927	19.08683
FLJ11460	6844	synaptobrevin 2	0.005929	18.93713
COLEC7	6441	pulmonary surfactant-associated protein D	0.006327	18.78743
BKLF3	11279	Kruppel-like factor 8	0.006534	18.63772
ADRA2	150	alpha2A adrenergic receptor	0.006748	18.48802
AD2	348	apolipoprotein E3	0.006815	18.26347
APOB	338	apolipoprotein B48	0.006815	18.26347
CAIN	8021	nucleoporin 214kDa	0.006872	17.96407
2PP2A	6418	template-activating factor I	0.006872	17.96407
ATP1A1	476	Na+/K+ ATPase 1	0.006923	17.73952
BACH1	571	BTB and CNC homology 1, basic leucine zipper	0.007038	17.58982
CBBM	5956	opsin 1 (cone pigments), long-wave-sensitive	0.00707	17.44012
DKFZp686E	6184	dolichyl-diphosphooligosaccharide-protein	0.007106	17.29042
BM600	3909	epiligrin alpha 3 subunit	0.007137	17.14072
ANX1	301	annexin I	0.007337	16.91617
CHAK	54822	transient receptor potential cation channel	0.007337	16.91617
ASIP	56288	atypical PKC isotype-specific interacting protein	0.007373	16.69162
APC3	996	cell division cycle protein 27	0.007431	16.54192
BS4	51667	NY-REN-18 antigen	0.007562	16.39222
KAT6A	7994	MYST histone acetyltransferase (monocytic leukemia)	0.007693	16.24251
TCL1	8115	T-cell leukemia/lymphoma 1A	0.007871	16.09281
ERBA-BETA	7068	generalized resistance to thyroid hormone	0.007931	15.94311

CPLX1	10815	complexin 1	0.008057	15.79341
AFP	174	alpha-1-fetoprotein	0.008084	15.64371
CFND	1947	ephrin-B1	0.008178	15.49401
CFL	1072	cofilin 1 (non-muscle)	0.008296	15.34431
MGC39961	10762	nucleoporin 50kDa	0.0087	15.19461
ABRI	9445	BRICHOS domain containing 2B	0.009075	15.04491
DKFZp686E	4131	microtubule-associated protein 1B	0.009162	14.89521
BM600-125	3914	laminin B1k chain	0.009506	14.67066
B2T	3918	laminin, gamma 2 (nicein (100kD), kalinin (105kD)	0.009506	14.67066
F9	2158	factor IX	0.009574	14.44611
C11orf43	3481	putative insulin-like growth factor II associated protein	0.009704	14.29641
C4	720	acidic C4	0.010354	13.84731
GPR24	2847	G-protein coupled receptor 24	0.010354	13.84731
C3B-INA	3426	light chain of factor I	0.010354	13.84731
C4A	721	basic C4	0.010354	13.84731
C5L2	27202	G protein-coupled receptor 77	0.010354	13.84731
DKFZp667C	10482	nuclear RNA export factor 1	0.01037	13.3982
A-152E5.1	6367	stimulated T cell chemotactic protein 1	0.010435	13.2485
LRP	9961	major vault protein	0.010449	12.9491
FLJ92613	2550	GABA-B receptor	0.010449	12.9491
FLJ36928	9568	G protein-coupled receptor 51	0.010449	12.9491
ARMD9	718	acylation-stimulating protein cleavage product	0.010545	12.6497
CMT1F	4747	neurofilament, light polypeptide 68kDa	0.010655	12.5
ADAP	2533	FYN-binding protein (FYB-120/130)	0.011037	12.3503
5-HT1A	3350	G protein coupled receptor	0.011549	12.2006
DKFZp781F	80314	enhancer of polycomb 1	0.011562	12.0509
LUN	10210	nucleoporin 50kDa	0.011658	11.9012
SCAR2	10163	suppressor of cyclic-AMP receptor (WASP-family)	0.011962	11.7515
CUL5	8065	Vasopressin-activated calcium-mobilizing receptor-1	0.012088	11.6018
ACTDP	11034	destrin	0.012746	11.4521
KIAA0965	23012	serine/threonine kinase 38 like	0.012749	11.3024
HRMT1L3	10196	protein arginine methyltransferase 3	0.012994	11.15269
BMP6	654	bone morphogenetic protein 6	0.013028	11.00299
CD3E	916	CD3e antigen, epsilon polypeptide (TiT3 complex)	0.013155	10.85329
RAB10	10890	ras-related GTP-binding protein RAB10	0.013201	10.70359
CBX	10951	heterochromatin protein p25 beta	0.013441	10.55389
ASBABP2	5069	pregnancy-associated plasma protein A, pappalysin 1	0.013695	10.40419
JDP2	122953	Jun dimerization protein 2	0.013695	10.25449
MGC33401	7073	TIA1 cytotoxic granule-associated RNA-binding	0.013714	10.10479
BAF170	6601	SWI3-like protein	0.014002	9.95509
DKFZp686L	55929	DNMT1 associated protein 1	0.015385	9.730539
DKFZp313C	9628	H_DJ1108A12.1	0.015385	9.730539
CLG1	4317	PMNL collagenase	0.015572	9.505988
GLK	8491	germinal center kinase-related protein kinase	0.0156	9.356287
C19orf3	10755	regulator of G-protein signalling 19 interacting protein 1	0.015797	9.206587
NEK6	10783	putative serine-threonine protein kinase	0.015825	8.982036
NEK7	140609	NIMA (never in mitosis gene a)-related kinase 7	0.015825	8.982036

KARS	3735	lysyl-tRNA synthetase	0.016024	8.757485
CD7	924	CD7 antigen	0.016248	8.607784
TLR7	51284	toll-like receptor 7	0.016251	8.458084
BMP14	8200	cartilage-derived morphogenetic protein-1	0.017	8.308383
ELA1	1990	pancreatic elastase I	0.017171	8.158683
KIAA1119	4645	myosin VB	0.018064	8.008982
K17	3872	keratin 17	0.018536	7.859281
HNRNPA1	3178	heterogeneous nuclear ribonucleoprotein core protein A1	0.02055	7.709581
CRSP150	9282	human homolog of yeast RGR1	0.022698	7.55988
GS2	5873	RAB27A, member RAS oncogene family	0.023049	7.41018
AGTAVPRL	114548	cryopyrin	0.023195	7.260479
HHR23B	5887	XP-C repair complementing complex 58 kDa	0.023221	6.511976
LPP	4026	LIM domain containing preferred translocation partner	0.023221	6.511976
PCTP	58488	phosphatidylcholine transfer protein	0.023221	6.511976
CREB3	10488	cyclic AMP response element (CRE)-binding protein	0.023221	6.511976
AIO	22806	aiolos	0.023221	6.511976
FLJ30414	3094	histidine triad nucleotide binding protein 1	0.023221	6.511976
CHA	10732	transcription factor-like 5 (basic helix-loop-helix)	0.023221	6.511976
DKFZp761M	83667	sestrin 2	0.023221	6.511976
SSTR5	6755	somatostatin receptor subtype 5	0.023221	6.511976
UNC5B	219699	transmembrane receptor Unc5H2	0.023589	5.763473
HsT17454	5607	MAP kinase kinase MEK5b	0.023976	5.613772
CD204	4481	macrophage scavenger receptor type III	0.02472	5.464072
DKFZp547M	23567	zinc finger protein 346	0.027892	5.314371
CAT	847	catalase	0.028643	5.164671
HDNF	4908	neurotrophin 3	0.028774	5.01497
PROP1	5626	prophet of Pit1, paired-like homeodomain	0.028904	4.865269
CD27	939	T cell antivation antigen S152	0.030161	4.715569
HNRPE2	5094	alpha-CP2	0.030375	4.416168
FLJ45792	23236	inositoltrisphosphohydrolase	0.030375	4.416168
SFRS3	6428	splicing factor, arginine/serine-rich, 20-kD	0.030375	4.416168
CYT4	27128	cytohesin-4	0.031011	4.116766
FLJ41337	8500	PTPRF interacting protein alpha 1	0.032555	3.967066
DLG3	1741	synapse-associated protein 102	0.032587	3.817365
CRTR1	29842	transcription factor CP2-like 1	0.03262	3.667665
CD281	7096	Toll/interleukin-1 receptor-like	0.032763	3.517964
ATSV	547	kinesin, heavy chain, member 1A, homolog of mouse	0.033071	3.368263
NKD2	85409	Dvl-binding protein NKD2	0.033372	3.218563
ERG1	3757	potassium channel HERG1	0.035442	3.068862
CRL	133396	gp130-like monocyte receptor	0.036669	2.919162
FLJ11090	55775	tyrosyl-DNA phosphodiesterase 1	0.036767	2.769461
DKFZp547M	79364	ZXD family zinc finger C	0.040633	2.54491
CHO1	9493	mitotic kinesin-like 1	0.040633	2.54491
CAP-3	5272	protease inhibitor 9 (ovalbumin type)	0.040718	2.320359
MGC16699	5797	protein tyrosine phosphatase, receptor type	0.041542	2.095808
CD113	25945	poliovirus receptor-related 3	0.041542	2.095808
DKFZp547K	5936	RNA binding motif protein 4	0.045361	1.871257

AMPH	273	Stiff-Man syndrome with breast cancer 128kDa	0.045807	1.721557
C3G	2889	guanine nucleotide-releasing factor 2	0.045905	1.422156
PPP2R3	5523	Serine/threonine protein phosphatase 2A	0.045905	1.422156
CDC2L2	728642	PITSLRE protein kinase beta	0.045905	1.422156
ACLP	165	aortic carboxypeptidase-like protein	0.046182	1.122754
EIF3-P42	8666	eukaryotic translation initiation factor 3 subunit p42	0.047311	0.973054
DKK-2	27123	dickkopf homolog 2	0.048118	0.823353
FLJ16302	4855	Notch homolog 4 (Drosophila)	0.048968	0.449102
CADASIL	4854	Notch homolog 3 (Drosophila)	0.048968	0.449102
AGS2	4853	Notch homolog 2 (Drosophila)	0.048968	0.449102
NOTCH1	4851	neurogenic locus notch homolog protein 1	0.048968	0.449102
CRES	10047	cystatin-related epididymal spermatogenic protein	0.049313	0.07485

Topologically significant genes from truncated set of proteomics data

Symbol	Entrez_ID	Description	p value	percentile
MYC	4609	myc proto-oncogene protein	2.27E-07	99.9115
DKFZp586H	51176	lymphoid enhancer binding factor-1	3.11E-07	99.46903
FLJ36364	6932	transcription factor 7 (T-cell specific, HMG-box)	3.11E-07	99.46903
TCF-3	83439	transcription factor 7-like 1 (T-cell specific, HMG-box)	3.11E-07	99.46903
TCF-4	6934	transcription factor 7-like 2 (T-cell specific, HMG-box)	3.11E-07	99.46903
EPHEXIN	25791	ephexin	5.53E-07	98.93805
MGC11154	5879	migration-inducing gene 5	5.53E-07	98.93805
NOR-90	7343	upstream binding transcription factor, RNA polymerase	5.79E-07	98.67257
FLJ12099	5602	mitogen-activated protein kinase 10	9.03E-07	98.49558
IRF-1	3659	interferon regulatory factor-1	1.12E-06	98.31858
GCCR	2908	glucocorticoid receptor	1.16E-06	98.14159
BCL2	596	B-cell CLL/lymphoma 2	1.19E-06	97.9646
HIRS-1	3667	insulin receptor substrate 1	1.33E-06	97.78761
BHLHB2	8553	differentially expressed in chondrocytes 1	1.8E-06	97.61062
MGC26306	9414	tight junction protein 2 (zona occludens 2)	1.81E-06	97.43363
CAP20	1026	CDK-interaction protein 1	2.03E-06	97.25664
ARF	1029	multiple tumor suppressor 1	2.27E-06	97.07965
DKFZp686H	3065	histone deacetylase 1	2.6E-06	96.90265
BP-8	4904	nuclease sensitive element binding protein 1	3.25E-06	96.72566
NOTCH1	4851	neurogenic locus notch homolog protein 1	3.43E-06	96.54867
LYT-10	4791	nuclear factor of kappa light chain gene enhancer	3.65E-06	96.37168
CEBP	64506	cytoplasmic polyadenylation element binding protein 1	3.77E-06	96.19469
AAA	351	amyloid beta A4 protein	3.83E-06	96.0177
DELTA	7528	YY1 transcription factor	4.16E-06	95.84071
IPOA1	3838	karyopherin alpha 2	4.39E-06	95.66372
G(gamma)1	51764	guanine nucleotide binding protein (G protein), gamma 13	4.97E-06	95.22124
GNB1	2782	transducin beta chain 1	4.97E-06	95.22124
GNG11	2791	guanine nucleotide-binding protein G(I)/G(S)/G(O)	4.97E-06	95.22124
KIAA1415	57580	phosphatidylinositol-3,4,5-trisphosphate	4.97E-06	95.22124
BMK1	5598	mitogen-activated protein kinase 7	5.3E-06	94.77876
HNRPE1	5093	poly(rC)-binding protein 1	5.37E-06	94.60177
HA6116	9759	histone deacetylase 4	5.63E-06	94.42478
MGC88021	5187	circadian pacemaker protein RIGUI	5.88E-06	94.24779
ETV3	2117	ets variant gene 3, ETS family transcriptional repressor	5.98E-06	94.0708
ENX-1	2146	enhancer of zeste homolog 2 (Drosophila)	6.07E-06	93.89381
AGO	55294	F-box and WD-40 domain protein 7	6.15E-06	93.71681
ASCL1	429	achaete-scute homolog 1	6.45E-06	93.53982
CASP8AP2	9994	human FLASH	6.47E-06	93.36283
DKFZp781F	9788	missing in metastasis	6.55E-06	93.18584
Hs.54452	10320	Ikaros (zinc finger protein)	6.58E-06	93.00885
FLJ36302	7074	T-cell lymphoma invasion and metastasis 1	7.1E-06	92.83186
MAZ	4150	zinc-finger protein, 87 kilodaltons	7.13E-06	92.65487
E2F-5	1875	E2F transcription factor 5, p130-binding	7.75E-06	92.47788
ENO1	2023	phosphopyruvate hydratase	7.97E-06	92.30088
CHEDG1	1901	sphingosine 1-phosphate receptor EDG1	8.09E-06	92.12389

KPD	5078	paired box gene 4	8.52E-06	91.9469
CBF1	3516	recombining binding protein suppressor of hairless	8.96E-06	91.76991
HsT17454	5607	MAP kinase kinase MEK5b	1.07E-05	91.59292
ARVD12	3728	catenin (cadherin-associated protein), gamma (80kD)	1.07E-05	91.41593
DKFZp779K	5291	PI3-kinase p110 subunit beta	1.08E-05	91.23894
AMPH2	274	bridging integrator 1	1.08E-05	91.06195
ELK4	2005	SRF accessory protein 1	1.1E-05	90.88496
BRCA1	672	breast and ovarian cancer susceptibility protein 1	1.1E-05	90.70796
ETS2	2114	oncogene ETS-2	1.17E-05	90.53097
CRD-BP	10642	insulin-like growth factor 2 mRNA binding protein 1	1.17E-05	90.26549
FBP	8880	far upstream element-binding protein	1.17E-05	90.26549
HES5	388585	hairy and enhancer of split 5 (Drosophila)	1.19E-05	90
DKFZp686C	4790	nuclear factor kappa-B, subunit 1	1.24E-05	89.82301
MGC13844	4772	nuclear factor of activated T-cells, cytoplasmic	1.27E-05	89.64602
BORIS	140690	CCCTC-binding factor-like protein	1.27E-05	89.46903
EMT	3702	tyrosine-protein kinase ITK/TSK	1.29E-05	89.29204
DKFZp686D	2931	glycogen synthase kinase 3 alpha	1.3E-05	89.02655
GSK3B	2932	glycogen synthase kinase 3 beta	1.3E-05	89.02655
GRIN1	2902	glutamate [NMDA] receptor subunit zeta 1	1.34E-05	88.76106
E2F-1	1869	retinoblastoma-associated protein 1	1.35E-05	88.58407
HSF1	3297	heat shock transcription factor 1	1.39E-05	88.40708
PP2Ac	5515	protein phosphatase 2, catalytic subunit, alpha isoform	1.4E-05	88.14159
PP2CB	5516	serine/threonine protein phosphatase 2A	1.4E-05	88.14159
DKFZp686B	6772	signal transducer and activator of transcription-1	1.46E-05	87.87611
I-REL	5971	v-rel avian reticuloendotheliosis viral oncogene	1.54E-05	87.69912
G0S24	7538	zinc finger protein, C3H type, 36 homolog (mouse)	1.79E-05	87.52212
CD104	3691	GP150	1.8E-05	87.25664
CD49f	3655	integrin alpha6B	1.8E-05	87.25664
ARP1	5308	solurshin	1.85E-05	86.99115
B1F	2494	liver receptor homolog-1	1.91E-05	86.81416
HOX-2.6	3214	homeobox B4	1.95E-05	86.63717
MSP23	5052	peroxiredoxin 1	1.96E-05	86.46018
FBL1	6502	CDK2/cyclin A-associated protein p45	2.09E-05	86.28319
KIAA1047	9611	nuclear receptor co-repressor 1	2.12E-05	86.10619
AAP1	23429	ring1 interactor RYBP	2.13E-05	85.9292
GRF-1	2909	glucocorticoid receptor DNA binding factor 1	2.2E-05	85.75221
AHR	196	aromatic hydrocarbon receptor	2.31E-05	85.57522
FLJ12859	23528	ZNP-99 transcription factor	2.43E-05	85.39823
ESA1	10524	cPLA2 interacting protein	2.44E-05	85.22124
PLZF	7704	zinc finger protein 145 (Kruppel-like)	2.74E-05	85.04425
CLOCK	9575	clock homolog (mouse)	2.77E-05	84.86726
DKFZp547C	25970	SH2B adaptor protein 1	2.82E-05	84.69027
E2F-4	1874	E2F transcription factor 4	2.91E-05	84.51327
MYD88	4615	myeloid differentiation primary response gene 88	2.97E-05	84.33628
FLJ10671	8295	350/400 kDa PCAF-associated factor	3.01E-05	84.15929
CDC25	5923	Ras-specific nucleotide exchange factor CDC25	3.19E-05	83.9823
MGC11098	6622	non A4 component of amyloid	3.21E-05	83.80531

POMP100	6421	polypyrimidine tract-binding protein-associated splicing factor	3.26E-05	83.62832
EGFR	1956	cell growth inhibiting protein 40	3.26E-05	83.45133
MGC13177	5970	v-rel avian reticuloendotheliosis viral oncogene	3.4E-05	83.27434
FLJ20819	7879	Ras-associated protein RAB7	3.43E-05	83.09735
MGC11121	4831	non-metastatic cells 2, protein (NM23) expressed in	3.45E-05	82.92035
CSBP	3190	transformation upregulated nuclear protein	3.8E-05	82.74336
CDC18L	990	CDC18 (cell division cycle 18, S.pombe, homolog)-like	3.92E-05	82.56637
NGR	65078	Nogo-66 receptor	4.11E-05	82.38938
RRAS	6237	Oncogene RRAS	4.18E-05	82.21239
FLJ25596	6655	son of sevenless homolog 2	4.23E-05	81.9469
GF1	6654	son of sevenless homolog 1	4.23E-05	81.9469
BAF170	6601	SWI3-like protein	4.32E-05	81.68142
BSP1	4086	MAD, mothers against decapentaplegic homolog 1	4.34E-05	81.50442
BCL3	602	B-cell leukemia/lymphoma 3	4.37E-05	81.32743
C/EBP-alph	1050	CCAAT/enhancer binding protein alpha	4.41E-05	81.15044
ELK3	2004	SRF accessory protein 2	4.53E-05	80.97345
VAV2	7410	Protein vav-2	4.65E-05	80.79646
ASCL2	430	achaete-scute complex-like 2	4.82E-05	80.61947
ARNTL	406	basic-helix-loop-helix-PAS orphan MOP3	4.84E-05	80.44248
MYL	5371	promyelocytic leukemia, inducer of	4.99E-05	80.26549
NR1I1	7421	vitamin D (1,25-dihydroxyvitamin D3) receptor	5.16E-05	80.0885
H-ICSBP	3394	interferon consensus sequence binding protein 1	5.39E-05	79.9115
PAK2	5062	p21-activated kinase 2	5.47E-05	79.73451
EZF	9314	endothelial Kruppel-like zinc finger protein	5.56E-05	79.55752
MSI1	4440	Musashi (Drosophila) homolog 1	5.61E-05	79.38053
PIM	5292	Oncogene PIM1	5.7E-05	79.20354
DPC4	4089	mothers against decapentaplegic homolog 4	5.81E-05	79.02655
CD227	4582	polymorphic epithelial mucin	5.82E-05	78.84956
ERYF1	2623	NF-E1 DNA-binding protein	5.87E-05	78.67257
DFNB24	5962	deafness, autosomal recessive 24	5.87E-05	78.49558
FLJ44809	2475	FKBP12-rapamycin complex-associated protein 1	5.97E-05	78.31858
HD3	8841	histone deacetylase 3	6.06E-05	78.14159
SREBF1	6720	sterol regulatory element binding transcription factor 1	6.12E-05	77.9646
ANCR	7337	ubiquitin protein ligase E3A	6.2E-05	77.78761
CD281	7096	Toll/interleukin-1 receptor-like	6.25E-05	77.61062
HNRNPU	3192	heterogeneous nuclear ribonucleoprotein U	6.28E-05	77.43363
AUF1	3184	heterogeneous nuclear ribonucleoprotein D	6.35E-05	77.25664
C1QA	712	complement component C1q, A chain	6.41E-05	76.90265
C1QB	713	complement component 1, q subcomponent,	6.41E-05	76.90265
C1Q-C	714	complement component 1, q subcomponent, C chain	6.41E-05	76.90265
FLJ16691	5829	paxillin	6.53E-05	76.54867
FARP2	9855	FGD1-related Cdc42-GEF	6.59E-05	76.28319
FLJ41865	26230	T-cell lymphoma invasion and metastasis 2	6.59E-05	76.28319
HDAC2	3066	YY1-associated factor 1	6.68E-05	76.0177
GEFT	115557	RAC/CDC42 exchange factor	6.72E-05	75.84071
DKFZp781H	5924	Ras protein-specific guanine nucleotide-releasing factor	6.75E-05	75.57522
GLUH1	2890	glutamate receptor, ionotropic, AMPA 1	6.75E-05	75.57522

ERBB4	2066	v-erb-a erythroblastic leukemia viral oncogene	6.86E-05	75.30973
AWD	4830	NDP kinase A	6.94E-05	75.13274
AT-V1	4683	p95 protein of the MRE11/RAD50 complex	6.96E-05	74.86726
H2A.X	3014	H2AX histone	6.96E-05	74.86726
ARF-BP1	10075	ARF binding protein 1	7.13E-05	74.60177
KIAA1515	57646	ubiquitin specific protease 28	7.21E-05	74.42478
AKT2	208	v-akt murine thymoma viral oncogene homolog 2	7.32E-05	74.24779
C21	79718	transducin (beta)-like 1X-linked receptor 1	7.38E-05	74.0708
DVL2	1856	dishevelled 2	7.43E-05	73.89381
DKFZp586M	4088	mad homolog JV15-2	7.48E-05	73.71681
AFBP	3484	amniotic fluid binding protein	7.5E-05	73.53982
FKBP38	23770	FK506 binding protein 8, 38kDa	7.5E-05	73.36283
E2F-6	1876	E2F transcription factor 6, isoform 1	7.56E-05	73.18584
DKFZp547J	1995	Hu antigen C	7.65E-05	73.00885
CK2A1	1457	casein kinase II alpha subunit	7.8E-05	72.74336
CK2A2	1459	casein kinase 2, alpha prime polypeptide	7.8E-05	72.74336
Cmyb	4602	c-myb13A_CDS	7.84E-05	72.47788
GIG34	6135	60S ribosomal protein L11	8.34E-05	72.30088
MIZ-1	7709	zinc finger protein 151 (pHZ-67)	8.54E-05	72.12389
BAPX2	4824	NK3 homeobox 1	8.59E-05	71.9469
ARHGEF14	23263	MCF2 transforming sequence-like protein	8.97E-05	71.76991
ELK1	2002	ELK1 protein	9.05E-05	71.59292
EAP1	9232	ESP1-associated protein 1	9.71E-05	71.41593
APLP	333	amyloid-like protein 1	9.82E-05	71.23894
CDK4I	1030	cyclin-dependent kinases 4 and 6 binding protein	9.86E-05	70.97345
MAD2	4601	Max-related transcription factor	9.86E-05	70.97345
APRF	6774	DNA-binding protein APRF	0.000103	70.70796
DDX9	1660	DEAD/H box-9 (nuclear DNA helicase II; RNA helicase A)	0.000106	70.53097
BAG-6	7917	scythe	0.000106	70.35398
ERBB2IP	55914	ERBB2 interacting protein	0.000115	70.17699
ARR3	407	cone arrestin	0.00012	70
BBC3	27113	BCL2 binding component 3	0.000122	69.82301
BID	637	BID isoform Si6	0.000123	69.64602
FKHL16	2305	Forkhead, drosophila, homolog-like 16	0.000124	69.46903
FASPS	8864	period circadian protein 2	0.000124	69.29204
BAM	10018	bcl-2 interacting protein Bim	0.000125	69.11504
ARF6	382	ADP-ribosylation factor 6	0.000131	68.93805
CHNG1	7253	thyroid stimulating hormone receptor	0.000132	68.76106
NOV	5361	plexin 1	0.000138	68.58407
C1	3183	heterogeneous nuclear ribonucleoprotein C (C1/C2)	0.000139	68.40708
CLGI	7076	fibroblast collagenase inhibitor	0.00014	68.23009
DTR	1839	heparin-binding epidermal growth factor	0.000143	68.0531
ERK	5594	protein tyrosine kinase ERK2	0.000149	67.78761
ERK1	5595	extracellular signal-regulated kinase 1	0.000149	67.78761
DUET	8997	serine/threonine kinase with Dbl- and pleckstrin	0.000154	67.52212
GIG8	3398	cell growth-inhibiting gene 8	0.000158	67.34513
CKBBP2	90480	papillomavirus L2 interacting nuclear protein 1	0.000163	67.16814

MGC12953	6097	nuclear receptor ROR-gamma	0.000163	66.99115
CINC-2a	2920	chemokine (C-X-C motif) ligand 2	0.000164	66.81416
CD309	3791	vascular endothelial growth factor receptor 2	0.000171	66.63717
60B8AG	6279	cystic fibrosis antigen	0.000171	66.46018
IRF3	3661	interferon regulatory factor 3	0.000172	66.28319
ADRBK1	156	beta adrenergic receptor kinase 1	0.000179	66.10619
CD221	3480	insulin-like growth factor 1 receptor	0.000183	65.84071
FOG	161882	friend of GATA-1	0.000183	65.84071
BLIMP1	639	beta-interferon gene positive-regulatory domain I	0.000184	65.57522
ACVRLK3	657	bone morphogenetic protein receptor, type IA	0.000187	65.39823
CRES	10047	cystatin-related epididymal spermatogenic protein	0.000191	65.13274
NEC2	5126	subtilisin-like prohormone convertases	0.000191	65.13274
ALK-6	658	bone morphogenetic protein receptor, type IB	0.000194	64.86726
MGC11118	5645	trypsin 2	0.000195	64.51327
MGC12017	5644	nonfunctional trypsin 1	0.000195	64.51327
MTG	5646	trypsin 3	0.000195	64.51327
KCIP-1	7534	phospholipase A2	0.000197	64.15929
ARHGEF2	9181	rho/rac guanine nucleotide exchange factor (GEF) 2	0.000198	63.9823
CD130	3572	CD130 antigen	0.0002	63.80531
INSRR	3645	IR-related receptor	0.000203	63.62832
GP145-TrkI	4915	neurotrophic tyrosine kinase, receptor, type 2	0.000203	63.45133
DOCK7	85440	dedicator of cytokinesis 7	0.000204	63.27434
MGC33401	7073	TIA1 cytotoxic granule-associated RNA-binding protein	0.000209	63.09735
ABD-B	3205	homeo box A9	0.000211	62.92035
AG2S	185	angiotensin receptor 1B	0.000212	62.74336
NF-E2	4778	nuclear factor (erythroid-derived 2), 45kDa	0.000214	62.56637
MTA1	9112	metastasis associated protein	0.000219	62.38938
DBL	4168	Oncogene MCF2 (oncogene DBL)	0.000223	62.21239
VASP	7408	vasodilator-stimulated phosphoprotein	0.000226	62.0354
CDC42	998	cell division cycle 42	0.000242	61.85841
GNG1	2792	guanine nucleotide binding protein (G protein), gamma transdu	0.000243	61.68142
JNK-55	5601	c-Jun kinase 2	0.000248	61.50442
BOS3	6495	SIX homeobox 1	0.000258	61.32743
FLT	2321	vascular endothelial growth factor	0.000258	61.15044
CD29	3688	integrin beta 1	0.00026	60.97345
GNA12	2768	guanine nucleotide binding protein (G protein) alpha 12	0.000262	60.79646
CP107	5933	retinoblastoma-like 1 (p107)	0.000264	60.61947
CD120a	7132	tumor necrosis factor receptor type 1	0.000264	60.44248
MGC70609	7422	vascular permeability factor	0.000268	60.26549
ARHGAP5	394	p100 RasGAP-associated p105 protein	0.000273	60.0885
AMPK	5563	AMP-activated protein kinase alpha 2 catalytic subunit	0.00028	59.82301
AMPK	5562	AMPK alpha 1	0.00028	59.82301
JNK	5599	stress-activated protein kinase JNK1	0.000282	59.55752
CED-12	9844	engulfment and cell motility 1	0.000282	59.20354
DOCK1	1793	dedicator of cyto-kinesis 1	0.000282	59.20354
DOCK2	1794	dedicator of cytokinesis 2	0.000282	59.20354
BETA-TRCP	8945	beta-TrCP1	0.000284	58.84956

FKLF	8462	Kruppel-like factor 11	0.000284	58.67257
FLJ16302	4855	Notch homolog 4 (Drosophila)	0.000295	58.49558
DVL3	1857	dishevelled 3	0.000296	58.31858
MAX	4149	MAX protein	0.000307	58.14159
C-Rel	5966	v-rel reticuloendotheliosis viral oncogene homolog	0.000322	57.9646
ARHG	391	ras homolog gene family, member G (rho G)	0.000323	57.61062
DOCK4	9732	dedicator of cytokinesis 4	0.000323	57.61062
GDS1	5910	RAP1, GTP-GDP dissociation stimulator 1	0.000323	57.61062
MKNK1	8569	MAP kinase interacting serine/threonine kinase 1	0.000324	57.25664
ARHGEF7	8874	rho guanine nucleotide exchange factor 7	0.000338	57.07965
ALPS1A	355	Fas (TNF receptor superfamily, member 6)	0.00034	56.90265
KIAA1907	3911	laminin alpha-5 chain	0.000341	56.72566
APRO6	10140	transducer of ERBB2, 1	0.000343	56.54867
CTS02	1513	cathepsin K	0.00035	56.37168
DELTA1	28514	delta-like 1	0.000357	56.19469
GPRC1E	2915	glutamate receptor, metabotropic 5	0.000361	56.0177
CRS2	4488	msh homeobox 2	0.000388	55.84071
HIF-1alpha	3091	member of PAS superfamily 1	0.000388	55.66372
D6S182	3326	heat shock 90kDa protein 1, beta	0.000388	55.48673
ANF	8820	homeobox, ES cell expressed 1	0.000405	55.30973
MODED	4613	neuroblastoma-derived v-myc avian myelocytomatosis	0.000408	55.13274
CALNB1	5534	protein phosphatase 3, regulatory subunit B	0.000408	54.86726
PPP3R2	5535	protein phosphatase 3, regulatory subunit B	0.000408	54.86726
CD295	3953	leptin receptor	0.00041	54.51327
FLJ94114	3952	obesity factor	0.00041	54.51327
ARNO	9266	cytohesin 2	0.000414	54.24779
156DAG	1605	dystrophin-associated glycoprotein-1	0.000423	54.0708
CTCF	10664	CTCFL paralog	0.000425	53.89381
2-Oct	5452	POU domain, class 2, transcription factor 2	0.000427	53.71681
ITSN	6453	intersectin short variant 12	0.000443	53.53982
ESP1	9700	extra spindle poles like 1	0.000444	53.27434
FLJ25655	5885	nuclear matrix protein 1	0.000444	53.27434
HsCYK-4	29127	GTPase activating protein	0.000451	53.00885
CSNBAD3	2779	transducin alpha-1 chain	0.000451	52.74336
DKFZp686C	5148	rod cG-PDE G	0.000451	52.74336
CADASIL	4854	Notch homolog 3 (Drosophila)	0.000454	52.47788
ECK	1969	protein tyrosine kinase	0.000456	52.30088
AR1	6942	stromelysin-1 platelet-derived growth factor-responsive element	0.000456	52.12389
CLG4B	4318	gelatinase B	0.000457	51.9469
CD126	3570	interleukin 6 receptor alpha subunit	0.000471	51.76991
APKD2	5311	polycystic kidney disease 2 (autosomal dominant)	0.000496	51.59292
ARID1A	8289	SWI/SNF related, matrix associated	0.000498	51.41593
ARNO3	9265	general receptor of phosphoinositides 1	0.000505	51.23894
CIG	2335	fibronectin 1	0.000506	51.06195
CD11C	3687	myeloid membrane antigen, alpha subunit	0.000518	50.70796
CD18	3689	complement receptor C3 beta-subunit	0.000518	50.70796
FGA	2243	fibrinogen, alpha polypeptide	0.000518	50.70796

DMP1	9988	cyclin D binding myb-like transcription factor 1	0.000521	50.35398
BSF2	3569	interleukin 6	0.000524	50.17699
KIAA1119	4645	myosin VB	0.000525	50
CD344	8322	WNT receptor frizzled-4	0.000536	49.82301
APRIL	8741	tumor necrosis factor-related death ligand-1	0.000537	49.64602
BCL10	8915	CARD-like apoptotic protein	0.00054	49.46903
60B8AG	6280	calgranulin B	0.00055	49.29204
GCN5	2648	GCN5 general control of amino-acid synthesis 5-like 2	0.000556	49.11504
CCL7	6354	chemokine (C-C motif) ligand 7	0.00057	48.93805
CCR1	1230	chemokine (C-C motif) receptor 1	0.00058	48.76106
MST155	51429	SH3 and PX domain-containing protein SH3PX1	0.00058	48.58407
ELN	2006	tropoelastin	0.000581	48.40708
CSK	1445	c-src tyrosine kinase	0.000595	48.23009
ACTA	58	alpha skeletal muscle actin	0.0006	48.0531
ABC-1	19	ATP binding cassette transporter 1	0.000602	47.78761
GIF	4282	phenylpyruvate tautomerase	0.000602	47.78761
ACTC	70	cardiac muscle alpha actin 1	0.000604	47.34513
CMD2A	7137	troponin I type 3 (cardiac)	0.000604	47.34513
MGC12565	5581	protein kinase C, epsilon	0.000604	47.34513
CMD1G	7273	titin	0.000607	46.99115
CC-CKR-2	1231	monocyte chemoattractant protein 1 receptor	0.000624	46.81416
PTPN2	5771	T-cell protein tyrosine phosphatase	0.000625	46.63717
GIOT-1	92283	zinc finger protein 461	0.000632	46.46018
BOB1	5450	POU class 2 associating factor 1	0.000647	46.28319
NMI	9111	N-myc-interactor	0.000652	46.10619
ACTRIIB	93	activin A type IIB receptor	0.000663	45.9292
ANCCA	29028	ATPase family, AAA domain containing 2	0.000665	45.75221
GRB1	5295	phosphoinositide-3-kinase, regulatory subunit	0.000669	45.48673
P85B	5296	phosphoinositide-3-kinase, regulatory subunit 2	0.000669	45.48673
ASP2	23621	memapsin-2	0.000681	45.22124
SREBF2	6721	sterol regulatory element-binding protein 2	0.000682	45.04425
CD51	3685	integrin alpha-V	0.000766	44.86726
FLJ94509	2672	zinc finger protein 163	0.000815	44.69027
CCNB	891	G2/mitotic-specific cyclin B1	0.000834	44.51327
CRK	1398	v-crk sarcoma virus CT10 oncogene homolog	0.000846	44.33628
ARNT	405	dioxin receptor, nuclear translocator	0.000848	44.15929
BAF190	6595	SWI/SNF-related matrix-associated actin-dependent	0.000862	43.9823
ASEF2	221178	adenomatous polyposis coli stimulated exchange factor 2	0.000885	43.80531
KIAA0407	5364	plexin B1	0.000888	43.62832
ARHGEF4	50649	Rho guanine nucleotide exchange factor 4	0.000894	43.45133
BCAR1	9564	Cas scaffolding protein family member 1	0.000901	43.27434
B2T	3918	laminin, gamma 2 (nicein (100kD), kalinin (105kD), BM600 (100	0.000983	43.09735
CUL4B	8450	cullin-4B	0.001034	42.92035
PTP-PEST	5782	protein-tyrosine phosphatase G1	0.001036	42.74336
CD124	3566	interleukin-4 receptor alpha chain	0.00106	42.47788
CD132	3561	common cytokine receptor gamma chain	0.00106	42.47788
ASIP	56288	atypical PKC isotype-specific interacting protein	0.001086	42.21239

CXCL12	6387	chemokine (C-X-C motif) ligand 12	0.001141	42.0354
FLJ22252	64321	SRY (sex determining region Y)-box 17	0.001158	41.85841
ERBB3	2065	v-erb-b2 avian erythroblastic leukemia viral oncogene	0.001191	41.68142
CFND	1947	ephrin-B1	0.001219	41.50442
B56A	5525	serine/threonine protein phosphatase 2A	0.001227	41.32743
CDCD1	4000	limb girdle muscular dystrophy 1B	0.001245	41.15044
ECP54	8607	RuvB (E coli homolog)-like 1	0.001254	40.97345
ACVRL1	94	activin A receptor, type II-like kinase 1	0.001265	40.79646
FLJ97193	10023	proto-oncogene FRAT1	0.001268	40.61947
GSC	145258	goosecoid	0.001285	40.44248
CCL2	6347	small inducible cytokine A2	0.001294	40
CCL3	6348	small inducible cytokine A3 (homologous to mouse)	0.001294	40
CCL3L1	6349	chemokine (C-C motif) ligand 3-like 1	0.001294	40
CCL3L3	414062	small inducible cytokine A3-like 1	0.001294	40
CBBM	5956	opsin 1 (cone pigments), long-wave-sensitive	0.0013	39.55752
H2A.z	3015	H2AZ histone	0.001311	39.38053
BCGF-1	3565	B cell growth factor 1	0.00132	39.20354
DNMT3A	1788	DNA (cytosine-5-)-methyltransferase 3 alpha	0.001324	39.02655
ADAM33	80332	a disintegrin and metalloprotease 33	0.001368	38.84956
AXIL	8313	axin 2	0.001394	38.58407
AXIN	8312	axin 1	0.001394	38.58407
LUN	10210	topoisomerase I binding, arginine/serine-rich	0.001419	38.31858
HXB	3371	tenascin C	0.001446	38.14159
TGFBRAP1	9392	TGF beta receptor associated protein -1	0.001497	37.9646
BMP2	650	bone morphogenetic protein 2	0.001506	37.69912
COL1A1	1277	alpha 1 type I collagen	0.001506	37.69912
14-3-3	10971	14-3-3 protein T-cell	0.001512	37.43363
2-5-3p	23265	exocyst complex component 7	0.001529	37.25664
ICK	22858	MAK-related kinase	0.001538	37.07965
DKFZp781F	80314	enhancer of polycomb 1	0.001558	36.90265
FCER1G	2207	Fc fragment of IgE, high affinity I, receptor	0.00157	36.72566
TLR7	51284	toll-like receptor 7	0.00168	36.54867
ACVRIP1	9863	membrane associated guanylate kinase, WW and PDZ	0.001745	36.37168
AGER	177	advanced glycosylation end product-specific receptor	0.001781	36.19469
ACC-4	1512	cathepsin H	0.001785	36.0177
GNA-11	2767	guanine nucleotide-binding protein, Gq class, GNA11	0.001796	35.84071
IBP5	3488	insulin-like growth factor binding protein 5	0.001862	35.66372
ACVR1C	130399	activin receptor-like kinase 7	0.001865	35.48673
ACAN	176	large aggregating proteoglycan	0.001878	35.30973
CAPRIN1	4076	membrane component chromosome 11 surface marker 1	0.001926	35.13274
ALPS2	843	caspase 10	0.001937	34.95575
CTSS	1520	cathepsin S	0.001958	34.77876
HGS	9146	hepatocyte growth factor-regulated tyrosine kinase	0.001958	34.60177
MBP	4155	myelin basic protein	0.002017	34.42478
CCL5	6352	T-cell specific RANTES protein	0.002112	34.24779
ON	6678	secreted protein, acidic, cysteine-rich	0.002114	34.0708
AMY-1	26292	associate of myc-1	0.002151	33.89381

ACTN2	88	F-actin cross-linking protein	0.002227	33.71681
ADAP1	11033	centaurin-alpha	0.002243	33.53982
AGS2	4853	Notch homolog 2 (Drosophila)	0.002307	33.36283
FLJ12850	57650	KIAA1524	0.002327	33.18584
AR-JP	5071	parkin	0.002365	33.00885
RNF81	6737	Sjogren syndrome antigen A1 (52kDa, ribonucleoprotein autoa	0.002388	32.83186
ATOD4	9021	suppressor of cytokine signaling 3	0.002553	32.65487
FLJ20922	5900	ral guanine nucleotide dissociation stimulator	0.00257	32.47788
CLASPIN	63967	claspin	0.002587	32.30088
ACPA	5657	proteinase 3 (serine proteinase, neutrophil)	0.002691	32.12389
CSNK1E	1454	casein kinase 1 epsilon	0.002906	31.9469
CASP14	23581	caspase 14	0.003023	31.76991
APAF-3	842	caspase 9, apoptosis-related cysteine protease	0.003027	31.59292
COL4A3	1285	alpha 3 type IV collagen	0.003088	31.41593
EMC19	6500	transcription elongation factor B (SIII), polypeptide 1-like	0.003094	31.23894
DKFZp779M	5340	plasminogen	0.003118	31.06195
GDF8	2660	myostatin	0.00317	30.88496
BAPX1	579	bagpipe homeobox 1	0.003201	30.70796
CDCA7L	55536	transcription factor RAM2	0.003277	30.44248
CDK8	1024	CDK8 protein kinase	0.003277	30.44248
ASD3	4624	myosin heavy chain, cardiac muscle alpha isoform	0.003309	30.17699
BM600	3909	epiligrin alpha 3 subunit	0.003385	30
APBB1	322	amyloid beta (A4) precursor protein-binding, family B	0.003392	29.82301
CNBP	7555	zinc finger protein 9 (a cellular retroviral nucleic acid)	0.003395	29.64602
DKFZp781F	5325	PLAG-like 1	0.003403	29.46903
DKFZp686L	7342	upstream binding protein 1 (LBP-1a)	0.003475	29.29204
ARA24	5901	ras-related nuclear protein	0.003544	29.02655
CHC1	1104	regulator of chromosome condensation 1	0.003544	29.02655
DKFZp686J	4763	neurofibromin	0.003675	28.76106
CD36	948	scavenger receptor class B, member 3	0.003681	28.58407
CD63	967	CD63 antigen (melanoma 1 antigen)	0.00378	28.40708
HOX7	4487	msh homeobox 1	0.003829	28.23009
ARFGEP10(	9922	IQ motif and Sec7 domain 1	0.003837	28.0531
CARD12	58484	caspase recruitment domain family, member 12	0.003867	27.87611
DKFZp762F	55662	hypoxia-inducible factor 1, alpha subunit inhibitor	0.00389	27.69912
BAZ1B	9031	Williams-Beuren syndrome chromosome region 10	0.004055	27.52212
CLPS	1208	pancreatic colipase preproprotein	0.004085	27.34513
CD2AP	23607	CD2-associated protein	0.004142	27.16814
DLL4	54567	notch ligand DLL4	0.004146	26.99115
BSF3	23529	B-cell stimulating factor 3	0.004211	26.81416
PI5	5268	serpin peptidase inhibitor, clade B (ovalbumin)	0.004265	26.63717
A-FABP	2167	fatty acid binding protein 4, adipocyte	0.004294	26.46018
BMP-11	10220	growth differentiation factor 11	0.004297	26.19469
EDF	3624	inhibin, beta A (activin A, activin AB alpha polypeptide)	0.004297	26.19469
DKFZp686J	57496	myocardin-related transcription factor B	0.004334	25.9292
GAB2	9846	GRB2-associated binding protein 2	0.004352	25.75221
CAP	10580	sorbin and SH3 domain containing 1	0.004353	25.57522

AVPR1	552	arginine vasopressin receptor 1A	0.004402	25.39823
FADD	8772	Fas-associating death domain-containing protein	0.004573	25.22124
ERRL1	133522	peroxisome proliferator-activated receptor gamma	0.004612	25.04425
MGC:3978(	7188	RING finger protein 84	0.00482	24.86726
ATX1	6310	ataxin 1	0.005131	24.69027
BAT8	10919	euchromatic histone-lysine N-methyltransferase 2	0.005168	24.33628
HSP70-1	3303	heat shock-induced protein	0.005168	24.33628
HSP70-1B	3304	heat shock 70kD protein 1B	0.005168	24.33628
CSCD	1634	small leucine-rich protein 1B	0.005412	23.9823
APT1LG1	356	tumor necrosis factor (ligand) superfamily, member 6	0.005515	23.80531
MGC21659	7069	spot 14 protein	0.005659	23.62832
KIAA1303	57521	regulatory associated protein of mTOR	0.005698	23.45133
CCL24	6369	eotaxin-2	0.005706	23.27434
ARVD	7043	transforming growth factor, beta 3	0.005729	23.09735
CD113	25945	poliovirus receptor-related 3	0.005733	22.83186
MGC16699	5797	protein tyrosine phosphatase, receptor type	0.005733	22.83186
MAD7	6945	transcription factor-like 4	0.005754	22.56637
AFP	174	alpha-1-fetoprotein	0.005931	22.38938
DKFZp686C	8848	transforming growth factor beta-stimulated protein	0.00598	22.21239
CDX1	1044	caudal type homeobox transcription factor 1	0.006058	21.76991
ERF	2077	Ets2 repressor factor	0.006058	21.76991
EST01027	10628	thioredoxin interacting protein	0.006058	21.76991
HHO.C10	3215	homeo box B5	0.006058	21.76991
CCT-alpha	6950	T-complex protein 1, alpha subunit	0.006185	21.32743
CRC18	1630	deleted in colorectal cancer protein	0.006232	21.15044
KIAA0283	11122	protein tyrosine phosphatase, receptor type, T	0.006238	20.97345
FLJ40431	10451	vav 3 oncogene	0.006317	20.79646
C7orf16	10842	G-substrate	0.006421	20.61947
HSSOX6	55553	SRY-box containing gene 6	0.006423	20.44248
AMPH	273	Stiff-Man syndrome with breast cancer 128kDa	0.006979	20.26549
COE3	253738	early B-cell factor 3	0.006998	20.0885
ACTB	60	PS1TP5-binding protein 1	0.007052	19.9115
AAT6	59	actin, alpha 2, smooth muscle, aorta	0.007135	19.64602
ACT	72	alpha-actin 3	0.007135	19.64602
DKFZp434K	7442	transient receptor potential vanilloid 1b	0.007192	19.29204
SNAPAP	23557	SNARE associated protein snapin	0.007192	19.29204
KIAA0988	6904	tubulin-specific chaperone d	0.007262	19.02655
ABI-1	10006	nap1 binding protein	0.007293	18.84956
NR2C2	7182	nuclear receptor subfamily 2, group C, member 2	0.007413	18.67257
EFNB2	1948	ephrin B2	0.007515	18.49558
BMP6	654	bone morphogenetic protein 6	0.007556	18.31858
MEP1B	4225	endopeptidase-2	0.007683	18.14159
NKD2	85409	Dvl-binding protein NKD2	0.007748	17.9646
MAF	4094	Avian musculoaponeurotic fibrosarcoma (MAF)	0.007833	17.78761
KDP	65125	serine/threonine-protein kinase WNK1	0.007949	17.61062
LGMD1	9499	limb-girdle muscular dystrophy 1A	0.008247	17.43363
GP6	51206	glycoprotein VI (platelet)	0.00832	17.25664

FLOT1	10211	flotillin 1	0.008447	17.07965
B2-1	9267	homolog of secretory protein SEC7	0.008585	16.90265
EHK3	2045	Eph homology kinase-3	0.008588	16.72566
CHRD	8646	chordin	0.008711	16.54867
GHRF	2691	somatocrinin	0.008962	16.37168
87U6	2773	guanine nucleotide binding protein (G protein)	0.009199	16.10619
GNAI1	2770	Gi1 protein alpha subunit	0.009199	16.10619
AT3	4287	Machado-Joseph disease protein 1	0.009644	15.84071
ADRA2	150	alpha2A adrenergic receptor	0.0102	15.66372
E14	4863	nuclear protein, ataxia-telangiectasia locus	0.010458	15.48673
FLJ27265	3150	high-mobility group (nonhistone chromosomal)	0.010481	15.30973
IGIF	3606	interleukin-18	0.010487	15.13274
EAT2	117157	SH2 domain-containing molecule EAT2	0.010781	14.95575
DKFZp686A	3146	high-mobility group (nonhistone chromosomal)	0.010987	14.69027
HMG2	3148	high-mobility group box 2	0.010987	14.69027
FLJ93058	4688	neutrophil cytosolic factor 2 (65kD)	0.011107	14.42478
Fug1	5905	Ran GTPase activating protein 1	0.011577	14.24779
PFM2	11107	PR domain containing 5	0.011671	14.0708
MSK	150094	myocardial SNF1-like kinase	0.012007	13.89381
CFD	200576	1-phosphatidylinositol-4-phosphate 5-kinase	0.012079	13.62832
HSF2	3298	heat shock transcription factor 2	0.012079	13.62832
AOF2	23028	lysine (K)-specific demethylase 1	0.012149	13.36283
CD301	10462	macrophage C-type lectin	0.012385	13.18584
AD3L	5664	presenilin 2	0.013128	13.00885
CLR11.4	171389	NLR family, pyrin domain containing 6	0.013165	12.83186
AR7	7067	triiodothyronine receptor	0.013818	12.56637
ERBA-BETA	7068	generalized resistance to thyroid hormone	0.013818	12.56637
ARHGAP7	10395	START domain containing protein 12	0.013845	12.30088
ARHGAP2(	23092	GTPase regulator	0.013911	12.12389
CCBP2	1238	chemokine (C-C motif) receptor 9	0.014034	11.9469
CD350	11211	frizzled 10	0.014138	11.76991
IHPS1	4842	neuronal nitric oxide synthase	0.01438	11.50442
SSTR5	6755	somatostatin receptor subtype 5	0.01438	11.50442
C15	51316	placenta-specific 8	0.014465	11.23894
MEL-18	7703	zinc finger protein 144	0.014561	11.06195
FZD7	8324	frizzled homolog 7 (Drosophila)	0.014677	10.88496
ATP1A1	476	Na+/K+ ATPase 1	0.014814	10.70796
ZABC1	7764	zinc finger protein 217	0.015093	10.53097
LSP1	4046	F-actin binding and cytoskeleton associated protein	0.016518	10.35398
ERK3	6300	stress-activated protein kinase 3	0.016622	10.17699
APC3	996	cell division cycle protein 27	0.016837	10
CCK-B	887	CCK2 receptor	0.017404	9.823009
CCK	885	cholecystokinin	0.017706	9.646018
ARNT2	9915	aryl-hydrocarbon receptor nuclear translocator 2	0.01782	9.469027
MESP1	55897	mesoderm posterior 1 homolog (mouse)	0.018065	9.20354
TRP32	9352	thioredoxin-related 32 kDa protein	0.018065	9.20354
DKFZp686E	6184	dolichyl-diphosphooligosaccharide-protein	0.018484	8.938053

FGG	2266	fibrinogen, gamma polypeptide	0.019054	8.761062
CFL	1072	cofilin 1 (non-muscle)	0.019366	8.584071
CAP-3	5272	protease inhibitor 9 (ovalbumin type)	0.01941	8.40708
BP	3240	binding peptide	0.0196	8.230088
CRF-R	1394	seven transmembrane helix receptor	0.019693	7.964602
CRFR2	1395	corticotropin releasing hormone receptor 2	0.019693	7.964602
FLJ23903	4641	nuclear myosin I	0.020847	7.699115
CC10	7356	Uteroglobin (Clara-cell specific 10-kD protein)	0.02251	7.522124
CAPB	832	capping protein (actin filament) muscle Z-line, beta	0.022943	7.345133
APBA1	320	amyloid beta A4 precursor protein-binding, family A, member 1	0.023111	7.168142
FLJ33906	80317	zinc finger with KRAB and SCAN domains 3	0.024015	6.99115
CISK	23678	cytokine-independent survival kinase	0.024214	6.814159
GPRK1	6011	G protein-coupled receptor kinase 1	0.024755	6.637168
INPP5D	3635	signaling inositol polyphosphate 5 phosphatase SIP-145	0.026525	6.460177
CD266	51330	type I transmembrane protein Fn14	0.026735	6.283186
DLG3	1741	synapse-associated protein 102	0.027802	6.106195
HRMT1L3	10196	protein arginine methyltransferase 3	0.028235	5.929204
DADR	1812	dopamine receptor D1	0.028856	5.752212
K17	3872	keratin 17	0.028922	5.575221
BWCR	1028	cyclin-dependent kinase inhibitor 1C	0.02993	4.955752
CDP2	23316	cut-like 2	0.02993	4.955752
CSF2	1437	granulocyte-macrophage colony stimulating factor	0.02993	4.955752
GASP	9737	G protein-coupled receptor-associated sorting protein	0.02993	4.955752
HACE1	57531	HECT domain and ankyrin repeat containing, E3 ubiquitin prote	0.02993	4.955752
IPOA4	3839	importin-alpha-Q2	0.02993	4.955752
MAFF	23764	transcription factor MAFF	0.03051	4.336283
ART1	417	ADP-ribosyltransferase 2	0.031904	4.159292
OS9	10956	amplified in osteosarcoma	0.032109	3.982301
MZF-1	7593	zinc finger protein 42	0.035275	3.80531
C3B-INA	3426	light chain of factor I	0.035673	3.628319
FLJ36928	9568	G protein-coupled receptor 51	0.03581	3.362832
FLJ92613	2550	GABA-B receptor	0.03581	3.362832
E2(17)KB1	7321	ubiquitin-conjugating enzyme E2D 1	0.03581	3.00885
PNOC	5368	prepronociceptin	0.03581	3.00885
BGR	64581	C-type (calcium dependent	0.036606	2.743363
ADAP	2533	FYN-binding protein (FYB-120/130)	0.036644	2.566372
5-HT1A	3350	G protein coupled receptor	0.03735	2.389381
HDLCQ12	3990	lipase C	0.039504	2.212389
IFI-4	4938	2-5A synthetase 1	0.041655	2.035398
DKFZp686C	4703	nebulin	0.042107	1.858407
BCD541	6606	survival of motor neuron 1, telomeric	0.043264	1.504425
BCD541	6607	survival of motor neuron 2, centromeric	0.043264	1.504425
FBP2	8570	KH-type splicing regulatory protein	0.043264	1.504425
IKBB	4793	nuclear factor of kappa light polypeptide gene enhancer in B-ce	0.044939	1.150442
CAB1	782	dihydropyridine-sensitive L-type, calcium channel beta-1 subur	0.04509	0.973451
CACNB2	783	calcium channel, voltage-dependent, beta 2 subunit	0.045268	0.707965
HMOX2	3163	heme oxygenase (decycling) 2	0.045268	0.707965

CDGAP	57514	CDGAP	0.047466	0.442478
CACNB3	784	calcium channel, voltage-dependent, beta 3 subunit	0.049138	0.265487
Bx42	22938	nuclear protein SkiP	0.049704	0.088496
